# Competitive tuning: Competition's role in setting the frequency-dependence of Ca^2+^-dependent proteins

**DOI:** 10.1371/journal.pcbi.1005820

**Published:** 2017-11-06

**Authors:** Daniel R. Romano, Matthew C. Pharris, Neal M. Patel, Tamara L. Kinzer-Ursem

**Affiliations:** Weldon School of Biomedical Engineering, Purdue University, West Lafayette, IN, United States of America; University of Virginia, UNITED STATES

## Abstract

A number of neurological disorders arise from perturbations in biochemical signaling and protein complex formation within neurons. Normally, proteins form networks that when activated produce persistent changes in a synapse’s molecular composition. In hippocampal neurons, calcium ion (Ca^2+^) flux through N-methyl-D-aspartate (NMDA) receptors activates Ca^2+^/calmodulin signal transduction networks that either increase or decrease the strength of the neuronal synapse, phenomena known as long-term potentiation (LTP) or long-term depression (LTD), respectively. The calcium-sensor calmodulin (CaM) acts as a common activator of the networks responsible for both LTP and LTD. This is possible, in part, because CaM binding proteins are “tuned” to different Ca^2+^ flux signals by their unique binding and activation dynamics. Computational modeling is used to describe the binding and activation dynamics of Ca^2+^/CaM signal transduction and can be used to guide focused experimental studies. Although CaM binds over 100 proteins, practical limitations cause many models to include only one or two CaM-activated proteins. In this work, we view Ca^2+^/CaM as a limiting resource in the signal transduction pathway owing to its low abundance relative to its binding partners. With this view, we investigate the effect of competitive binding on the dynamics of CaM binding partner activation. Using an explicit model of Ca^2+^, CaM, and seven highly-expressed hippocampal CaM binding proteins, we find that competition for CaM binding serves as a tuning mechanism: the presence of competitors shifts and sharpens the Ca^2+^ frequency-dependence of CaM binding proteins. Notably, we find that simulated competition may be sufficient to recreate the *in vivo* frequency dependence of the CaM-dependent phosphatase calcineurin. Additionally, competition alone (without feedback mechanisms or spatial parameters) could replicate counter-intuitive experimental observations of decreased activation of Ca^2+^/CaM-dependent protein kinase II in knockout models of neurogranin. We conclude that competitive tuning could be an important dynamic process underlying synaptic plasticity.

## Author summary

Learning and memory formation are likely associated with dynamic fluctuations in the connective strength of neuronal synapses. These fluctuations, called synaptic plasticity, are regulated by calcium ion (Ca^2+^) influx through ion channels localized to the post-synaptic membrane. Within the post-synapse, the dominant Ca^2+^ sensor protein, calmodulin (CaM), may activate a variety of downstream binding partners, each contributing to synaptic plasticity outcomes. The conditions at which certain binding partners most strongly activate are increasingly studied using computational models. Nearly all computational studies describe these binding partners in combinations of only one or two CaM binding proteins. In contrast, we combine seven well-studied CaM binding partners into a single model wherein they simultaneously compete for access to CaM. Our dynamic model suggests that competition narrows the window of conditions for optimal activation of some binding partners, mimicking the Ca^2+^-frequency dependence of some proteins *in vivo*. Further characterization of CaM-dependent signaling dynamics in neuronal synapses may benefit our understanding of learning and memory formation. Furthermore, we propose that competitive binding may be another framework, alongside feedback and feed-forward loops, signaling motifs, and spatial localization, that can be applied to other signal transduction networks, particularly second messenger cascades, to explain the dynamical behavior of protein activation.

## Introduction

**Fig 1:**
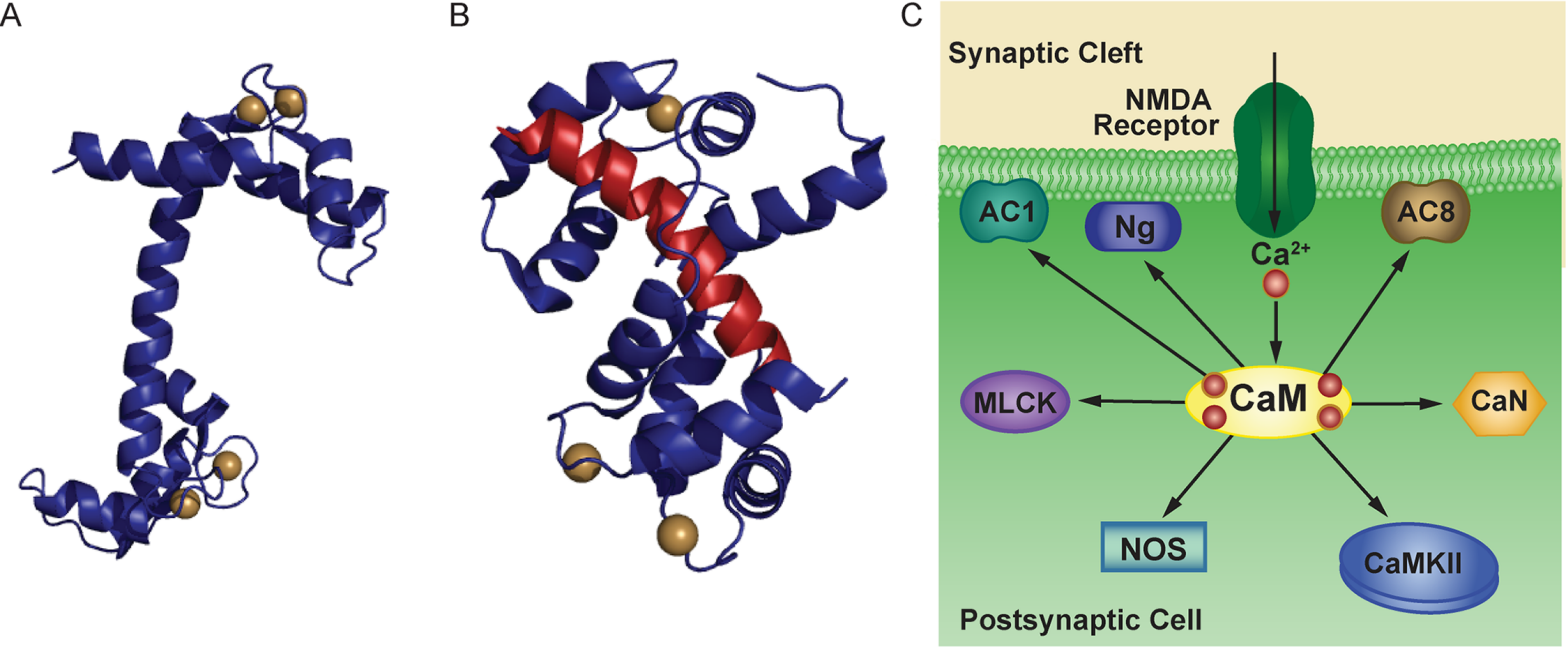
Schematic of CaM binding. (A) Structure of CaM (PDB 1CLL), shown in blue, with two Ca^2+^ ions (gold) at each terminus. (B) Structure of Ca^2+^/CaM (PDB 2JZI) bound to a calcineurin (CaN) peptide (red). (C) Schematic of CaM interactions with downstream binding partners. CaM may bind Ng in the absence of Ca^2+^. In the presence of Ca^2+^, CaM binds to CaN, CaMKII, NOS, MLCK, and AC1 and AC8.

Calcium (Ca^2+^) is well-recognized as an important second messenger in cellular signaling. One of the most widely expressed Ca^2+^ binding proteins, calmodulin (CaM), is a highly conserved protein in the EF-hand family [1] (Fig 1A). CaM has over 100 reported downstream binding proteins, including enzymes that regulate a variety of cellular functions, such as neurotransmitter release in presynaptic neuronal axons[2], insulin secretion in the pancreas [3], and contractility in muscle [4]. Ca^2+^-dependent signaling in postsynaptic dendrites of excitatory neurons has been the frequent subject of computational studies (see a recent review [5]). Indeed, it comprises an ideal system for mathematical modeling. Its parameters (molecular concentrations and kinetic rate constants) have been measured using controlled experiments, and experimental interest has produced an abundance of published values for model parameterization [4, 6–21]. Two highly-studied functions of synaptic Ca^2+^ signaling are the induction and maintenance of long-term potentiation (LTP) and long-term depression (LTD) [22], which are correlated to learning processes and memory storage in various brain regions [23–26]. Both LTP and LTD are accompanied by persistent changes in postsynaptic gene transcription [27], actin polymerization [28], and AMPA receptor trafficking [29] that adjust cellular excitability and, in turn, synaptic strength. Among the best-studied forms of LTP and LTD are those initiated by transient, localized increases in intracellular Ca^2+^ through postsynaptic N-methyl-D-aspartate receptors (NMDARs). CaM translates Ca^2+^ signals into either LTP or LTD by forming Ca^2+^/CaM complexes that bind and thereby activate downstream proteins (Fig 1C) [30]. Upon activation, these CaM-dependent proteins, which include a variety of enzymes—kinases, phosphatases, cyclases, and synthases—initiate protein signaling cascades that differentially modulate gene transcription, actin polymerization, and AMPA receptor trafficking.

The frequency [31], amplitude, duration, and location [32] of Ca^2+^ fluxes determine the pattern of activation of CaM-dependent enzymes and, in turn, the fate of the synapse. For example, 1 Hz stimulation for 10–15 minutes both increases activation of the CaM-dependent phosphatase calcineurin (CaN, or PP3) [33] and induces NMDAR-dependent LTD [34]. On the other hand, 100 Hz stimulation for 1 second increases Ca^2+^/CaM-dependent protein kinase II (CaMKII) activation and induces NMDAR-dependent LTP [35]. These and similar observations have led to the consensus that kinase cascades induce LTP, while phosphatase cascades induce LTD [36]. But more recent studies have found that CaN may also contribute to LTP induction [37], and that activated CaMKII can promote LTD [38]. These results suggest that normal initiation and maintenance of LTP and LTD do not simply depend on the Boolean activation of kinases or phosphatases in response to a given Ca^2+^ signal, but rather on the precise activation of a variety of often-counteracting proteins. Therefore, elucidation of the mechanisms that regulate NMDAR-dependent long-term plasticity depends on a complete understanding of the endogenous tuning mechanisms that pair precise patterns of enzyme activation to certain Ca^2+^ signals.

Computational studies have demonstrated the role of binding dynamics [39], feedback loops [40], and spatial effects [41] in regulating enzyme activation during synaptic Ca^2+^ signaling. In this work, we hypothesize that competition among CaM binding proteins for access to CaM may serve as an additional tuning mechanism. The concentration of CaM binding partners in the cell far exceeds that of CaM itself [42], and *in vitro* studies have demonstrated competitive inhibition among neuronal CaM binding partners [43–45]. But, despite the implicit presence of competition in many computational models of Ca^2+^/CaM signaling in neurons [41, 46–51] and cardiac myocytes [52–56], just one study [46] has had the explicit aim of investigating competition among CaM binding partners as a regulator of enzyme activation. Antunes *et al*. use such a model to investigate competitive binding as a potential facilitator of the frequency-dependence of CaM binding partners at low frequency Ca^2+^ fluxes (5 mHz to 5 Hz) for generalized sets of CaM binding partners. However, it is worth noting that both He et. al. and Slavov et. al. both mention competition for CaM as a part of their broader studies on the frequency dependent behavior of networks of generalized CaM targets [51] and relative activation of kinase versus phosphatase signaling [50].

In this work we develop models of Ca^2+^ binding to CaM that explicitly includes Ca^2+^-binding to each of the two termini (N- and C-termini, Fig 1). Previous experimental work has shown that CaM is able to activate downstream binding proteins at sub-saturating levels of Ca^2+^[57]. Moreover, a previous computational study explicitly including Ca^2+^-binding to each of the two binding sites (N- and C-termini) of CaM has shown that Ca^2+^ bound at the C-terminus likely significantly contributes to activation of downstream binding partners [39]. Our models also include seven experimentally-characterized postsynaptic CaM binding proteins expressed in CA1 hippocampal neurons. These mathematical models are used to investigate competition’s potential role as a regulator of Ca^2+^-dependent protein activation across a range of Ca^2+^ flux frequencies (0.1 Hz to 1000 Hz) that spans those found *in vivo* and oft employed experimentally *in vitro*. Specifically, we first develop a set of “isolated” models simulating CaM binding to Ca^2+^ and just one binding protein. We then combine the isolated models into a “competitive” model that simulates Ca^2+^ binding to CaM and CaM binding to its binding partners. The CaM binding proteins in this study have been chosen because they are known neuronal proteins with relatively well-characterized CaM-binding kinetics: adenylyl cyclase type I (AC1), the adenylyl cyclase type VIII N-terminus (AC8-Nt), the adenylyl cyclase type VIII C_2b_ domain (AC8-Ct), calcineurin (CaN, also known as PP2B and PP3), CaMKII, myosin light chain kinase (MLCK), neurogranin (Ng), and nitric oxide synthase (NOS) (Fig 1C). Because our model is devoid of feedback loops and spatial localization, the differences in CaM-binding between the competitive and isolated models are solely due to competitive effects. We demonstrate the ability of competition to “tune” the binding and activation profiles of CaM-binding proteins at various Ca^2+^ flux frequencies and use the model to explain the counterintuitive role of neurogranin in CaMKII activation and LTP induction.

## Results

### Model development

#### Model structure

**Fig 2:**
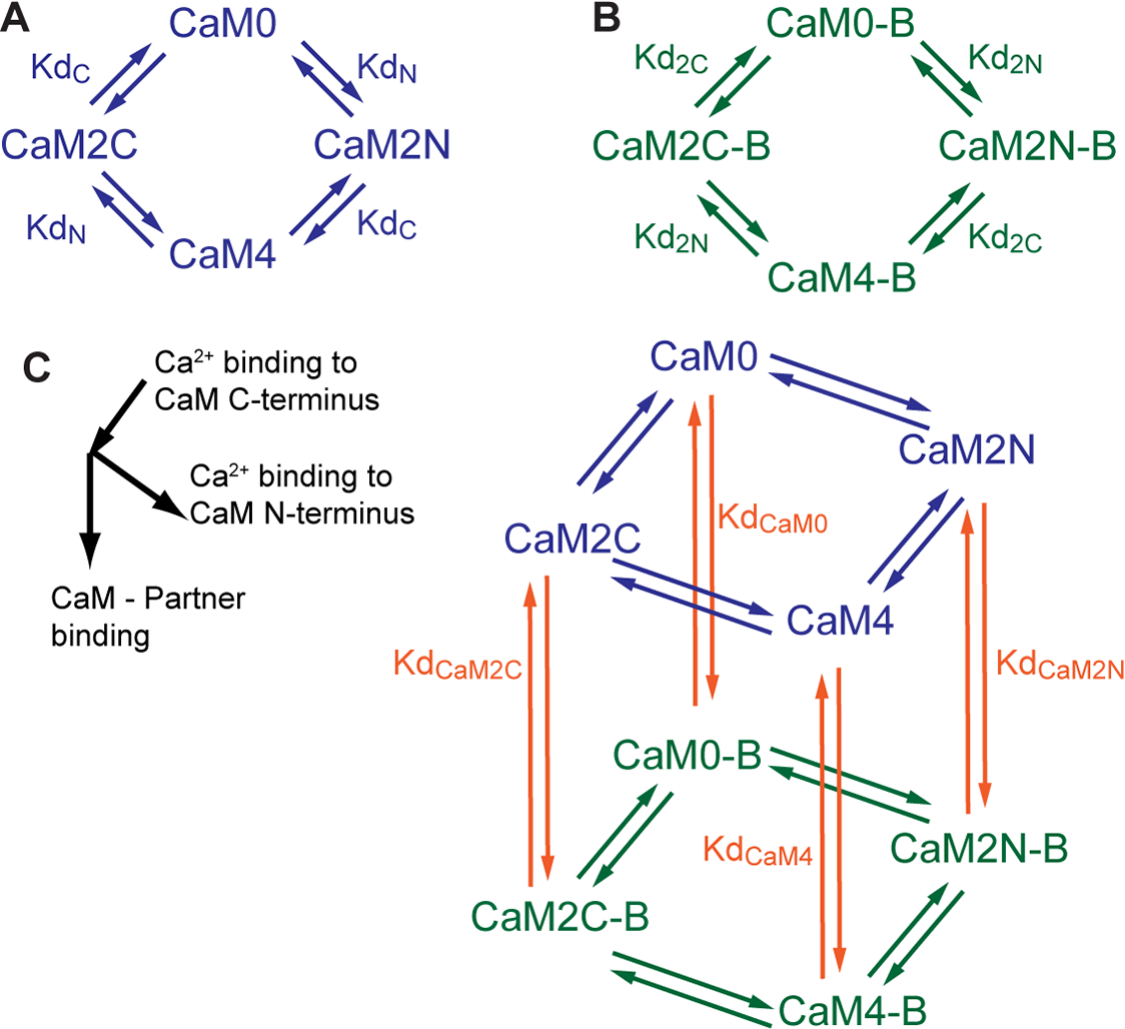
Model of Ca^2+^/CaM interactions. (A) Reversible binding of Ca^2+^ binding to CaM (blue). (B) Reversible binding of Ca^2+^ to CaM bound to a given binding partner, denoted with ‘B’ (green). (C) Reversible binding of a given binding partner to any state of Ca^2+^/CaM (orange).

The interactions of Ca^2+^, CaM, and CaM binding partners are quite complex. CaM binds a total of four Ca^2+^ ions, one pair at each of two EF-hand domains located at its N- and C-termini, respectively (Fig 1A and schematically in Fig 2A) [1]. Ca^2+^-binding at each terminus is highly cooperative [58], but the Ca^2+^-binding kinetics between these termini are distinct [21]. Moreover, the binding of Ca^2+^ to CaM changes its affinity for downstream binding partners. Similarly, the binding of CaM to its binding partners changes its affinity for Ca^2+^ (Fig 2B) [30]. We develop a mathematical model based on mass action kinetics that uses ordinary differential equations to simulate the dynamics of: Ca^2+^ ions binding reversibly to CaM, the dynamics of CaM binding reversibly to its binding partners, and the dynamics of Ca^2+^ ions binding reversibly to CaM when CaM is bound to a binding partner [39, 59] (Fig 2).

A previous study by Pepke *et al*. offers two models for describing Ca^2+^-CaM binding. First, they describe a four-state model in which it is assumed that binding of two Ca^2+^ at each CaM terminus can be treated as a single event due to the highly cooperative binding of Ca^2+^ at each terminus. Alternatively, a nine-state model is presented that explicitly accounts for each Ca^2+^ binding event, for which further details are discussed in S1 Appendix. In the present study, we construct both model types and simulate CaM-binding of seven proteins implicated in hippocampal-dependent memory and long-term plasticity [35–37, 60–74]. We find that differences in the output of the four- and nine-state models are negligible for the purposes of this work (Fig S1 and discussion in S1 Appendix). To reduce computational complexity, all model results are based on a four-state model of Ca^2+^-CaM binding.

#### Model parameterization

Initial concentrations of all proteins were either obtained directly from literature or calculated from published values. Equilibrium dissociation (K_D_) constants not available in the literature are calculated (see [39, 75]) using the thermodynamic principle of microscopic reversibility. From the dissociation constants, any unmeasured kinetic rates are calculated using the equality: K_D_ = k_off_/k_on,_ and a pair of experimentally-supported assumptions regarding Ca^2+^, CaM, and binding partner interactions.

First, it is well known that the affinity of Ca^2+^ for CaM is increased when CaM is bound to a binding partner (CaM-B) [14, 30]. The change in affinity could be represented by either an increase in the association rate constant of Ca^2+^ for CaM-B or a decrease in the dissociation rate constant of Ca^2+^ for CaM-B. Experimental work by Peersen *et al*. showed that the increased affinity of Ca^2+^ to target peptide-bound CaM was best explained by a reduction in Ca^2+^ dissociation rate constant [14]. More recent work has shown in the case of Ng binding to CaM, that the change in affinity for Ca^2+^ results primarily from a change in the dissociation rate constant [76]. Thus, we assume that the increase in affinity of Ca^2+^ for CaM when CaM is bound to a binding partner comes from a change in the dissociation, but not association, rate constant of Ca^2+^ from CaM-B.

Second, the binding of Ca^2+^ to CaM increases the affinity of CaM for most of its binding proteins [30, 77, 78], with the notable exception of Ng. Like others ([39, 41]), we note experimental observations showing that Ca^2+^ dissociation from CaM typically precedes CaM dissociation from binding proteins [79], indicating that the increase in affinity of CaM for most of its binding proteins in the presence of Ca^2+^ may be due to an increase the association (and not the dissociation) rate constant of CaM binding to target proteins. Biophysically, Ca^2+^ binding to CaM induces a conformational change that exposes hydrophobic patches that then facilitate binding to hydrophobic residues on the target proteins [1, 80, 81]. These Ca^2+^-binding induced structural changes on CaM could be thought of as increasing the probability of successful binding to a target protein, which would translate to an increased association rate. Thus, we implement an assumption that Ca^2+^-binding changes the association, but not dissociation, rate of CaM to most of its binding partners (with the exception of Ng). It should be noted that it is likely that the increases in affinity discussed above come from changes in both the association and dissociation rate constant parameters. Current experimental techniques are unable to measure the kinetic rate constants of apo-CaM binding to target proteins (again, with the exception of Ng), and so the exact quantitative values or even relative changes in affinity and dissociate rate constants are unknowable at this time. The assumptions implemented here are our best-educated interpretation of current biophysical understanding of Ca^2+^, CaM and CaM-target binding.

Published rate constant values that were obtained using full-length proteins are used preferentially over those for oligopeptides, but oligopeptide values are included in setting the physiological ranges for sensitivity analyses. Values for simulations are the geometric means of published values, or derived values (listed in S1 Table). Geometric means were chosen as opposed to arithmetic means so that outlier values less significantly biased the parameter values in the simulations.

#### Adenylyl Cyclase Type I and VIII (AC1 and AC8)

AC1 and AC8 are 123 kDa and 135 kDa [82] membrane-spanning enzymes expressed in the CA1 pyramidal cells of mammalian hippocampus [83, 84]. The primary function of both AC1 and AC8 is the formation of the second messenger cyclic-AMP from ATP [85]. CaM activation of AC1 is dependent upon the binding of Ca^2+^/CaM [86] to a single site in its C_1b_ domain [87]. Work by D. Cooper and colleagues has shown that CaM binding to the C_1b_ domain on AC1 requires participation from both the N- and C-lobes of CaM [6, 88]. CaM activation of AC8 is also dependent upon Ca^2+^/CaM-binding [84], but unlike AC1, each AC8 enzyme contains binding sites at both its N-terminus and C_2b_ domain [89]. C_2b_-binding is the major contributor to CaM-dependent activation of AC8 and can be substantially activated by binding of the Ca^2+^-bound N-lobe of CaM [6, 88]. A peptide derived from AC8-C_2b_ was able to pull down a CaM with mutations that prohibited Ca^2+^-binding at the C-lobe at similar levels to WT CaM [6]; indicating that the N-lobe of CaM mediates most of the binding interaction between CaM and the C-terminus of AC8 (AC8-Ct). In contrast, similar pulldown experiments indicate that CaM binding to the N-terminus of AC8 (AC8-Nt) is mediated by the C-lobe of CaM [6]. CaM binding to AC8-Nt does not activate AC8’s enzymatic activity [6], but has been suggested but CaM-binding at the N-terminus may support activation by increasing the local CaM concentration in a “CaM trapping” mechanism [89]. Each of these binding sites associates to Ca^2+^/CaM in a 1:1 stoichiometry [90]. Therefore, we model AC8 as a pair of distinct targets, AC8-Nt and AC8-Ct. The concentrations of AC1 and AC8 in CA1 pyramidal cells have been estimated at 42.2 and 41.9 μM, respectively [41]. For all simulations, a concentration of 42 μM is used for AC1, AC8-Nt, and AC8-Ct. All kinetic parameters are either obtained from literature [6, 41] or calculated using previously-described assumptions.

#### Calcineurin (CaN)

CaN is a 78 kDa [7], PSD-associated [91] enzyme expressed in the CA1 pyramidal cells of mammalian hippocampus [92]. As a heterodimer [93], CaN activation is dependent upon both the association of the catalytic subunit CaNA to the regulatory subunit CaNB as well as the binding of CaNA to Ca^2+^/CaM [10] in a 1:1 stoichiometry [94]. Although CaNB is a Ca^2+^-binding protein, Ca^2+^ binding to CaNB does not affect the affinity of CaNA for either CaNB [93] or Ca^2+^/CaM [10]. For this reason, the binding of both CaNA and Ca^2+^ to CaNB are neglected in our model. CaN dephosphorylates the residues of many cellular proteins, including AMPA receptors, NMDARs, protein kinase A, and inhibitor-1 [95]. The concentration of CaN in the hippocampus is 36.4 mg of protein per kg of tissue [96]. Assuming an average protein concentration of 100 mg/mL, or 10% by mass [39], the density of CaN in hippocampus was calculated at 36.4 μg/mL, corresponding to a concentration of 0.47 μM. Here, a concentration of 0.5 μM is used. All kinetic parameters are either obtained from literature [7, 8, 10] or calculated based on previously-described assumptions.

#### Ca2+/CaM-dependent protein kinase II (CaMKII)

CaMKII is a PSD-associated [97] enzyme expressed in CA1 pyramidal cells of the mammalian hippocampus [98]. As a 650 kDa dodecamer, CaMKII is composed of twelve catalytic subunits [99]. In the hippocampus, the alpha isoform of CaMKII comprises approximately two-thirds of these subunits, while the beta isoform constitutes the remaining one-third [99]. The activation of each of these subunits is dependent upon the binding of Ca^2+^/CaM [100] in a 1:1 stoichiometry [101], such that the full dodecamer binds Ca^2+^/CaM in a 1:12 ratio [102]. CaMKII phosphorylates the residues of many cellular proteins, including synapsin I, pyruvate kinase, phenylalanine hydroxylase, tyrosine hydroxylase, phospholamban, MLCK, and MAP-2 [103]. CaMKII monomers can also phosphorylate intramolecular neighbors in an autophosphorylation process [104]. The resulting autophosphorylated CaMKII, termed autonomous CaMKII, remains partially active even after dissociating from Ca^2+^/CaM [105]. Because our model is non-spatial and generally ignores catalytic processes, CaMKII is modeled in its monomeric form (i.e., as separate, independent subunits). The local concentration of catalytic CaMKII subunits in the dendritic spines of CA1 pyramidal cells has been previously estimated at 74 μM [39]. All kinetic parameters are obtained from literature [39].

#### Myosin light chain kinase (MLCK)

MLCK is a 146 kDa [106] enzyme expressed in CA1 hippocampal dendrites [69]. Its activation is dependent upon the binding of Ca^2+^/CaM [107] in a 1:1 stoichiometry [108]. MLCK phosphorylates the regulatory light chain of the molecular motor myosin II [109]. The concentration of MLCK in the hippocampus has not been measured, but it has been observed to be much less than that in smooth muscle [110], where its concentration is about 50 μM [111]. Therefore, its concentration in CA1 pyramidal cells is estimated as one order-of-magnitude less, or 5 μM. Because the amino acid sequence of neuronal MLCK is almost identical to that of smooth muscle MLCK [110], we used the kinetic parameters of smooth muscle MLCK in our model. All kinetic parameters are either obtained from literature [4, 11, 13, 14] or calculated based on previously-described assumptions.

#### Neurogranin (Ng)

Ng is a 7.8 kDa [112], membrane-associated [113] protein expressed in high quantities in the dendritic spines of CA1 pyramidal cells in the mammalian hippocampus [114]. Ng binds apo-CaM in a 1:1 stoichiometry [115]. It has no enzymatic function [116] but has been found to localize CaM to the cell membrane [117], theoretically resulting in the spatial coupling of CaM to both Ca^2+^ channels and CaM-dependent enzymes. The concentration of Ng in hippocampus has been estimated at 65 μM [73]. All kinetic parameters are obtained from literature [59].

#### Nitric oxide synthase (NOS)

NOS is a 155 kDa [118], PSD-associated [119] enzyme expressed in CA1 pyramidal cells of the mammalian hippocampus [120]. Its activation is dependent upon the binding of Ca^2+^/CaM [121] in a 1:1 stoichiometry[19]. NOS catalyzes the formation of nitric oxide and citrulline from arginine [121]. The active form of NOS is a homodimeric complex [122]. However, because our model is non-spatial and generally ignores catalytic processes, NOS is modeled in its monomeric form. NOS is found in 100x diluted, homogenized rat striatum at a density of 0.7 μg/mL [123], corresponding to a concentration of 0.45 μM. Because the density of NOS is 1.5 times greater in the CA1 region of hippocampus than in striatum [124], and because NOS is localized to dendritic spines, a concentration of 1 μM is used. All kinetic parameters are either obtained from literature [15, 18], or calculated based on previously described assumptions.

#### Calcium (Ca2+)

In response to a single presynaptic action potential, the transient opening of postsynaptic NMDARs in hippocampal dendritic spines generates a single spike in free Ca^2+^ concentration that peaks at 12 μM and, as Ca^2+^ is rapidly buffered, decays with a time constant of 12 milliseconds [125]. Therefore, free Ca^2+^ fluxes into the system by the equation, [Ca](t) = 12e^-t/0.012^. In our model, this function is a fixed boundary condition, meaning that the total Ca^2+^ concentration in the system is not conserved over the course of the simulation. Free Ca^2+^ is introduced into simulations at frequencies ranging from 0.1 Hz to 1 kHz, which spans one order-of-magnitude past the range of frequencies used in LTD- and LTP-inducing experimental protocols [33, 34]. Before the introduction of free Ca^2+^ into the system, all simulations are run to steady state for 600 seconds to equilibrate Ca^2+^-independent binding events.

### Model analysis

We use the total concentration of CaM-bound protein as a primary output parameter. This is contrary to most published computational models, which investigate the concentration of Ca^2+^-saturated CaM (CaM_4_) bound to each protein. This approach is preferred for three main reasons. First, although most CaM-dependent enzymes are maximally activated by binding CaM_4_, sub-saturated forms of CaM have also been found to activate these enzymes, albeit at a lower catalytic rate [57]. Therefore, the concentration of CaM_4_-bound enzyme does not represent the total concentration of active enzyme. Second, not all binding sites in our model increase in catalytic activity upon CaM binding. For these proteins (Ng and AC8-Nt) the CaM_4_-bound concentration is no more relevant than the concentration bound to apo-CaM or, for that matter, any other sub-saturated form. Third, CaM-binding to non-catalytic sites has been found to influence CaM availability to CaM-dependent enzymes [89, 117], suggesting an important physiological role for minimally-active, yet still CaM-bound, enzymes. Therefore, although the total concentration of CaM bound to each binding site is not a direct measure of its activation, it provides important information about patterns of enzyme activation that cannot be inferred from the concentration bound to CaM_4_ alone. To obtain a representative measure of total CaM-binding during Ca^2+^ spiking at a particular frequency, the average value (henceforth designated the average bound concentration, C_b_) is calculated by Eq 1:

$$C_{b}=\frac{1}{t_{f}-t_{0}}\int_{{t=t}_{0}}^{t_{f}}\overset{2}{\underset{i=0}{\sum }}\overset{2}{\underset{j=0}{\sum }}\left[T_{b}CaMN_{i}C_{j}\right]dt$$ (1)

$$T_{b}=\{AC1\ldots NOS\}$$

Where the subscript b indexes the binding partners, so the average bound concentration for a given binding partner (C_b_) is found by integrating the total concentration of that binding partner (T_b_) bound to each CaM state (CaMN_i_C_j_, i and j = 0, 1, or 2) over the stimulation period (t_o_ until t_f_) and dividing by the stimulus duration (t_f_—t_o_). To measure relative levels of CaM-binding across various proteins and experimental conditions, for each binding partner we normalize C_b_ by its peak value from among all the Ca^2+^ frequencies simulated.

We observe that for competitive models, the frequency range at which C_b_ peaks may shift or narrow relative to the isolated case. To quantify this tuning, we define a metric of frequency specificity (S_b_), where the subscript b indexes the binding partners. A binding partner with high frequency specificity is one that most significantly binds CaM over a narrow range of frequencies; correspondingly, this binding partner’s frequency-dependence curve would have a tall, narrow peak. First, the frequency-dependence curve is integrated and then normalized by the maximum C_b_ (Eq 2). We also divide by the total simulated frequency range and subtract from 1 to report S_b_ as a metric that identifies the most strongly tuned binding partners. In Eq 2, f denotes Ca^2+^ frequency.

$$S_{b}=1-\frac{1}{\left(\log{(f}_{f})-\log{(f}_{0}))\;max[C_{b}\right]}\;\int_{f_{0}}^{f_{f}}C_{b}(f)\;df$$ (2)

#### Sensitivity analysis

To determine which parameters most greatly impacted our models’ outputs and, therefore, which may benefit most from further characterization in future experiments, we conducted two sets of global sensitivity analyses using Latin Hypercube sampling (LHS) to efficiently sample the input parameter space and partial rank correlation coefficients (PRCC) to quantify the results [75]. In one set, we fixed the kinetic rate constants and investigated the impact of variations in initial concentrations on the average bound concentrations (C_b_) of the eight CaM binding partners. In the second, we fixed the initial concentrations and investigated the impact of variations in kinetic rate constants. Each of these analyses was performed at low (1 Hz), moderate (10 Hz), and high (100 Hz) frequency Ca^2+^ oscillations, allowing us to observe how the impacts of parameter variations change with frequency (see S2 Appendix). To control for total Ca^2+^ introduced, oscillations were limited to 10 concentration spikes, regardless of frequency. In Tables 1 and 2, we present the results of our 10 Hz sensitivity analysis, listing the parameters that most strongly influence each C_b_.

**Table 1:** Significant PRCCs for initial protein concentration parameters.

Output	[Varied Input Parameter] PRCC Value			
**C**_**AC1**_	[CaM] 0.9509	[AC1] 0.9204	[CaMKII] -0.8177	
**C**_**AC8-Ct**_	[CaM] 0.9498	[AC8-Ct] 0.9209	[Ng] -0.8752	[CaMKII] -0.5186
**C**_**AC8-Nt**_	[CaM] 0.9567	[AC8-Nt] 0.9116	[Ng] -0.7441	
**C**_**CaN**_	[CaM] 0.9564	[CaN] 0.9442	[CaMKII] -0.8999	
**C**_**CaMKII**_	[CaM] 0.9766	[CaMKII] 0.9177	[AC8-Nt] -0.6657	
**C**_**MLCK**_	[CaM] 0.9381	[MLCK] 0.934	[CaMKII] -0.7392	[Ng] -0.6167
**C**_**Ng**_	[CaM] 0.9759	[Ng] 0.8827		
**C**_**NOS**_	[NOS] 0.996	[CaM] 0.8076	[Ng] -0.7238	

Enumeration of Partial Rank Correlation Coefficient (PRCC) values for variations in initial protein concentrations that most strongly affect each average CaM-bound protein concentration, C_b_, for simulations with Ca^2+^ frequency of 10 Hz. Only inputs with absolute PRCC values greater than 0.5 are shown.

**Table 2:** Significant PRCCs for rate parameters.

Output	Varied Input Parameter				
	PRCC Value				
**C**_**AC1**_	k_on_^AC1CaM4^ 0.9243	k_on_^KCaM4^ -0.8964	k_on_^1N^ 0.5449	k_off_^AC1CaM4^ -0.5271	
**C**_**AC8-Ct**_	k_on_^AC8ctCaM4^ 0.8295	k_on_^KCaM4^ -0.7473	k_on_^1N^ 0.6305	k_on_^AC8ctCaM2N^ 0.5914	k_on_^NgCaM2N^ -0.5065
**C**_**AC8-Nt**_	k_on_^AC8ntCaM2C^ 0.8725 k_off_^AC8ntCaM2C^ -0.5663	k_off_^KCaM2C^ 0.7294	k_off_^K2N^ 0.703	k_on_^1N^ -0.6864	k_on_^NgCaM2C^ -0.5816
**C**_**CaN**_	k_on_^PPCaM4^ 0.9649	k_on_^KCaM4^ -0.8123			
**C**_**CaMKII**_	k_off_^KCaM2C^ -0.8187	k_off_^K2N^ -0.7581	k_on_^KCaM4^ 0.733	k_on_^AC8ntCaM4^ -0.5377	k_on_^1N^ 0.5002
**C**_**MLCK**_	k_on_^1N^ 0.642	k_on_^KCaM4^ -0.8983	k_on_^MKCaM4^ 0.9376		
**C**_**Ng**_	k_off_^NgCaM0^ -0.6711 k_on_^2N^ -0.6259	k_on_^Ng2C^ -0.6645 k_off_^NgCaM2C^ -0.6093	k_on_^Ng1C^ -0.6641 k_on_^NgCaM2C^ 0.5302	k_off_^Ng1C^ 0.6444 k_on_^NgCaM0^ 0.5021	k_off_^Ng2C^ 0.6358
**C**_**NOS**_	k_on_^NOSCaM4^ 0.8964	k_off_^NOSCaM0^ -0.6958	_kon_^KCaM4^ -0.5681		

Enumeration of Partial Rank Correlation Coefficient (PRCC) values for variations in rate parameters that most strongly affect each average CaM-bound protein concentration, C_b_, for simulations with Ca^2+^ frequency of 10 Hz. Only inputs with absolute PRCCs greater than 0.5 are shown.

Although their experimental ranges span several orders of magnitude, the kinetic binding constants of CaM_4_ binding to CaN (k_on_^PPCaM4^ and k_off_^PPCaM4^), as well as CaM_4_ binding to NOS (k_on_^NOSCaM4^ and k_off_^NOSCaM4^_)_, only significantly affected the C_b_ of CaN and NOS, respectively. In contrast, the rate constant of CaM_4_ binding to CaMKII (k_on_^KCaM4^), despite having an experimental range that varies only four-fold, significantly impacted almost all outputs of CaM binding partners at each of the three frequencies. Future competitive computational models may benefit from more accurate measurement of k_on_^KCaM4^ than from the more accurate measurement of k_on_^PPCaM4^, k_off_^PPCaM4^, k_on_^NOSCaM4^, or k_off_^NOSCaM4^, despite the clear experimental uncertainty in measurements of the latter four.

### Competition regulates CaM-binding dynamics

**Fig 3:**
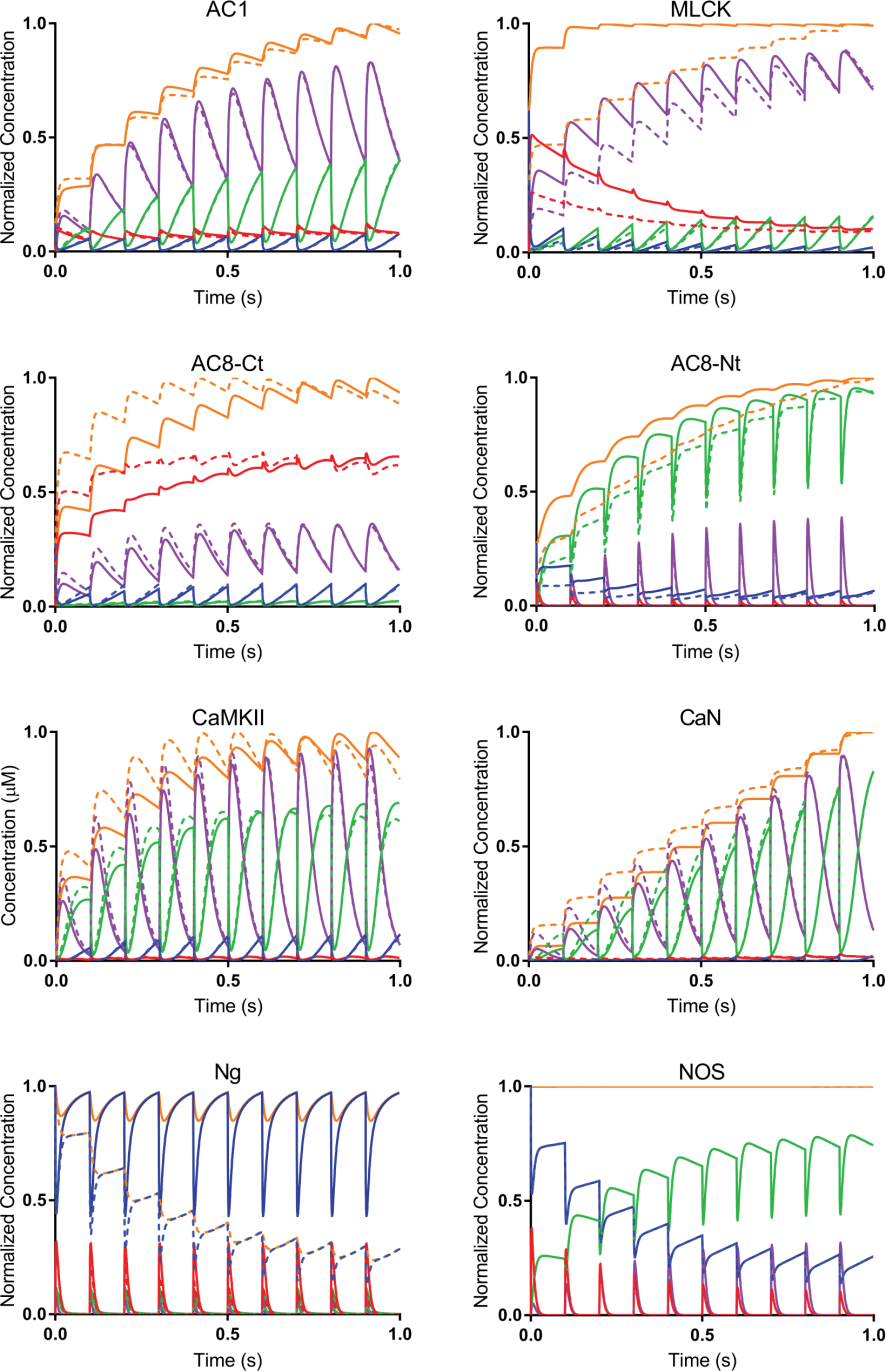
Competition for CaM alters binding dynamics. Time-course of CaM binding partners bound to various states of CaM for 1 second of 10 Hz Ca^2+^ flux: CaM_0_ (blue), CaM_2N_ (red), CaM_2C_ (green), CaM_4_ (purple), and CaM_tot_ (orange). CaM_tot_ is the sum of all CaM-bound states for a given protein. The concentration of each species is normalized against its maximum value of CaM_tot_. Solid lines denote the isolated model. Dotted lines denote the competitive model. The differences between isolated and competitive behavior are more significant for some CaM binding partners than others.

To investigate how competition alters the CaM-binding dynamics of each of the eight binding partners, we plotted the normalized concentrations of individual partners bound to different CaM states: apo-CaM (CaM_0_), CaM bound to two Ca^2+^ ions at its N-terminus (CaM_2N_), CaM bound to two Ca^2+^ ions at its C-terminus (CaM_2C_), and CaM_4_ (Fig 3). In each simulation, 10 Ca^2+^ fluxes (not plotted) were introduced at 10 Hz, corresponding to the logarithmic midpoint of our chosen frequency range. In Fig 3, the different colors of the plotted traces correspond to the concentration of binding partner bound to each of the four CaM states normalized to the total concentration of all CaM-bound binding partner (CaM_tot_). The time-course of CaM binding partners bound to various states of CaM in micromolar for 1 second of 10 Hz Ca^2+^ flux is plotted in Fig S3 in S1 Appendix.

As expected, the presence of competitors decreases the concentration of CaM bound to each binding partner. Because the relative contributions of the various CaM states to each binding partner’s CaM_tot_ in the competitive model were similar to those in the isolated model, competition did not appear to have a disproportionately large effect on the binding of any one CaM state. This suggests that CaM, and not Ca^2+^, is the major limiting factor in the activation of CaM-dependent enzymes in hippocampal dendritic spines. Furthermore, competition appears to change not just the concentration of CaM bound to each partner, but also the CaM-binding dynamics. To paraphrase, concentrations in the competitive model are not simply scaled versions of their counterparts in the isolated model. Instead, competition seems to change how each binding partner responds to rapid Ca^2+^ transients, including how CaM-binding changes with each subsequent Ca^2+^ flux. For example, after just three Ca^2+^ fluxes, the concentration of CaM-bound MLCK no longer changes in the isolated model, while it continues to increase in the competitive model. Conversely, while the CaM-binding of Ng decreases with each subsequent Ca^2+^ spike in the competitive model, it does not change in the isolated model. Therefore, the dynamic behavior of CaM targets in cellular environments cannot necessarily be inferred from computational studies that model them in isolation.

Finally, although competition attenuates the CaM-binding of all binding partners, the magnitude of their attenuation varies considerably in our model. For example, while NOS experiences virtually no change in CaM-binding in the presence of competitors, CaN experiences a more than 20-fold reduction in CaM-binding in the competitive model. Therefore, the binding partners are unequally competitive under the simulated conditions. From these observations, we hypothesize that the competitiveness of each binding partner (i.e., the ability of a binding partner to bind CaM in the presence of other binding partners) might not be absolute and, instead, that the competitiveness of each protein may change across environmental conditions. In this case, competition for CaM is well-positioned to serve as a tuning mechanism, suppressing the CaM-binding of each binding partner for all but a small range of internal conditions and external stimuli and allowing for the tight control of enzyme activation needed for the precise regulation of LTP, LTD, and other neurological processes. Therefore, we investigate how competition may tune the CaM-binding of each neuronal protein to certain Ca^2+^ frequencies.

### Competition tunes CaM binding to certain Ca^2+^ frequencies

**Fig 4:**
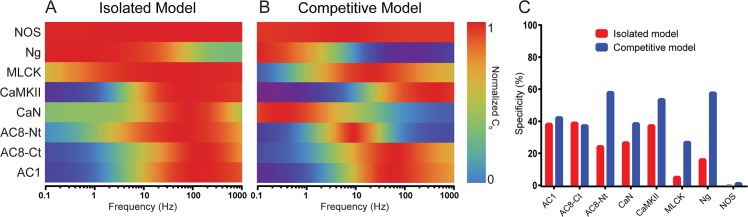
Competition tunes activation frequencies. (A) and (B) show normalized activation of CaM as a function of frequency for the isolated and competitive models, respectively. Red denotes peak activation; blue denotes minimal activation. Frequency windows of peak activation tend to narrow and shift for many of the binding partners in the competitive case. Indeed, (C) indicates a sharpening of activation frequency windows as an increase in specificity in the competitive model, at least for most proteins. Specificity is S_b_ multiplied by 100 percent.

To investigate our hypothesis that competition affects the frequency-dependence of CaM-binding, we construct frequency-dependence curves for all eight CaM binding sites (distinguishing between each AC8 terminus) using both the isolated and competitive models (Fig S2 in S1 Appendix). The frequency dependence of C_b_ is then projected onto heat maps (Fig 4A and 4B). For all simulations, Ca^2+^ oscillations consisted of 100 concentration spikes ranging from 0.1 Hz to 1 kHz.

The introduction of competition shifts the frequency-dependence curves of almost all binding partners. For some, such as AC1, AC8-Ct, CaMKII, and MLCK, this shift is slight, but apparent. For other partners, such as AC8-Nt and CaN, this shift is dramatic. In the competitive model, maximal CaM-binding occurs at frequencies almost one order of magnitude lower for AC8-Nt (10 Hz in the competitive model, as compared to 60 Hz in the isolated model). For CaN, maximal CaM-binding occurs at frequencies over two orders of magnitude lower (0.3 Hz in the competitive model, as compared to 80 Hz in the isolated model). For NOS, a frequency shift is present but not visible in Fig 4B.

Although, as stated earlier, total CaM-binding and enzymatic activation are not the same (particularly for CaN, which is subject to dual regulation by Ca^2+^/CaM and CaNB), it is worth noting that CaN is activated by low, but not high, frequency stimulation *in vivo* [33]. Therefore, it would be expected that maximal CaM-binding of CaN occurs at a similarly low frequency. The fact that this held true in the competitive, but not in the isolated, model suggests that the *in vivo* frequency-dependence of CaN may be reliant upon the presence of cellular competitors. Because of both the established role of CaN in LTD induction [36, 64] and the demonstrated ability of low frequency stimulation to induce LTD [33], our results further suggest that competition for CaM may be essential to normal LTD induction. Furthermore, because activated CaN downregulates LTP induction [63], competitive suppression of CaM-binding to CaN at high frequencies may be equally essential to normal LTP induction.

To investigate the effects of competition on each CaM binding partner’s level of preference for a certain frequency range, we used the frequency-dependence curves to calculate the frequency specificity of each binding partner in both the isolated and competitive models as defined in Eq 2. If a binding partner were only active at one frequency, it would have a frequency specificity of 100 percent.

The introduction of competitors sharpens the frequency-dependence curves of almost all binding partners, as also indicated by increased frequency specificity values in the competitive models relative to the isolated models (Fig 4C); frequency specificities increased for AC1 (42.80%, as compared to 38.73%), AC8-Nt (58.55%, as compared to 24.79%), CaN (39.13%, as compared to 27.19%), CaMKII (54.17%, as compared to 37.89%), MLCK (27.45%, as compared to 5.70%), Ng (58.23%, as compared to 16.64%), and NOS (1.77%, as compared to 0.08%). The sole decrease, AC8-Ct, was small (37.86%, as compared to 39.50%). Therefore, competition for CaM not only regulates CaM-binding by changing the frequencies of maximal CaM binding, but also by narrowing the range over which appreciable CaM binding occurs.

### Competition for CaM mediates Ng/CaMKII crosstalk

Two studies have reported decreased CaMKII autophosphorylation and CaMKII activity in CA1 hippocampal slices harvested from Ng genetic knockout (Ng^-/-^) mice [126, 127]. Although both studies reported about a 30% decrease in CaMKII autophosphorylation and CaMKII activity, they were in disagreement concerning the effect of the genetic knockout (Ng^-/-^) on LTP induction. Pak *et al*. (2000) found that wild type (Ng^+/+^) mice required a single tetanus to achieve potentiation, while Ng^-/-^ mice required multiple tetanic stimulations [126]. In direct contrast, Krucker *et al*. (2002) found that Ng^-/-^ mice required only a single tetanus to induce LTP [127]. Despite these inconsistent results, both sets of authors suggested that this phenomenon may be caused by abnormal regulation of local Ca^2+^ and CaM concentrations, a proposal that has since been supported by several studies.

For example, Huang *et al*. (2004) attributed diminished LTP in Ng^-/-^ mice to lower levels of free Ca^2+^ following high frequency stimulation [73]. And using two sets of Ng mutants which, respectively cannot bind, and constitutively bind, CaM, Zhong *et al*. (2009) provided evidence that abnormal regulation of local CaM concentrations may also be responsible. Using a model of the interactions of Ca^2+^, CaM, CaMKII, CaN, and AMPARs, Zhabotinsky *et al*. (2006) reproduced the effects of Ng knockout on LTP induction reported by Huang *et al*., but did not address the diminished CaMKII activity reported by both Pak *et al*. and Krucker *et al*. To date, no mathematical model has replicated the paradoxical effect of Ng genetic knockout on autonomous CaMKII activity.

**Fig 5:**
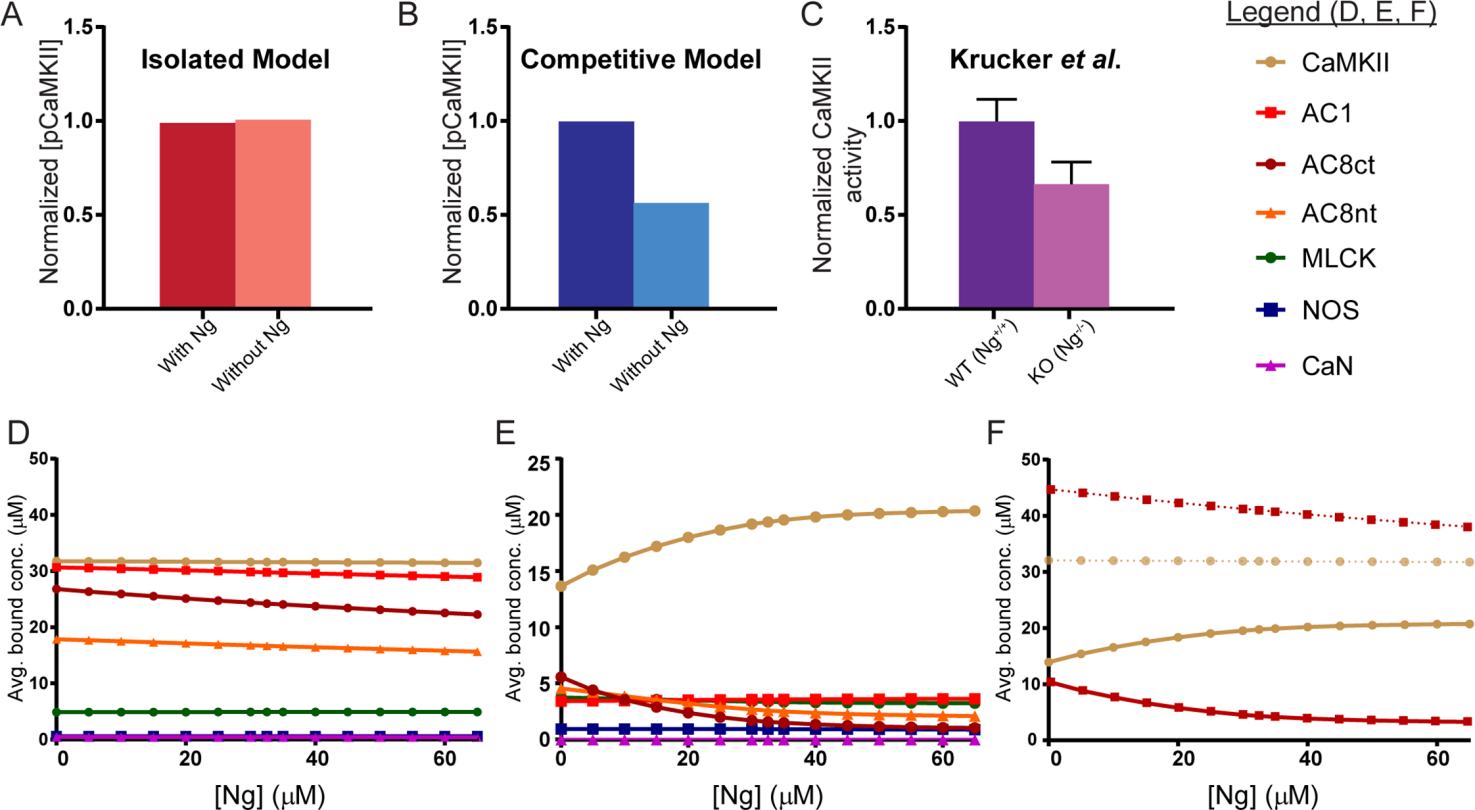
Competitive tuning explains intermolecular crosstalk. (A) Simulations of CaMKII phosphorylation in our isolated model with and without inclusion of Ng. (B) Simulations of CaMKII phosphorylation in our competitive model with and without Ng. (C) CaMKII activity in WT and Ng^-/-^ knockout mice from Krucker *et al*. Simulations were performed to replicate the experimental method of Krucker *et al*. as closely as possible. (D) The average bound concentration (C_b_) of each CaM binding protein in semi-isolated models as a function of Ng concentration. AC8-Ct and AC8-Nt exhibit the greatest relative change in CaM-binding (C_b_, Eq 1) as Ng concentration decreases. (E) The average bound concentration (C_b_) of each CaM binding protein in the competitive model as a function of Ng concentration. For a decreasing Ng concentration, AC8-Ct and AC8-Nt again exhibit the greatest relative change in CaM-binding. (F) Comparing the semi-isolated (dotted traces) to the competitive (solid traces) model shows that only in the competitive model does summed AC8 (AC8-Nt + AC8-Ct, dark red) mirror the loss in CaM-CaMKII binding as Ng concentration decreases.

We hypothesize that these phenomena could be explained by competitive tuning. We simulate autonomous CaMKII activation by extending our model according to a previously-published model of CaMKII autophosphorylation by Pepke *et al*. (see Fig 6 in [39]). In that work, two CaM-bound (active) CaMKII monomers form a complex that enzymatically catalyzes the phosphorylation of one of the monomers. We stimulate this extended model according to an LTP induction protocol followed by Krucker *et al*., in which hippocampal slices were subjected to two tetanic stimuli of 100 pulses at 100 Hz, 20 seconds apart. Using this protocol, we assess our isolated (Fig 5A) and competitive (Fig 5B) models’ responses to simulated Ng knockout at 600 seconds after the last stimulus. Normalized results from the same experimental stimulation protocol by Krucker *et al*. are shown in Fig 5C (see activity data in Fig 1F in [127]).

In the absence of other competitors, the isolated model elicits similar levels of CaMKII autophosphorylation (pCaMKII) whether in the presence or absence of Ng. That is, the complete removal of Ng, which competes with CaMKII for CaM, results in only a slight increase in pCaMKII (Fig 5A). In contrast, in the presence of competitors for CaM, simulated Ng knockout decreases pCaMKII levels by 44% compared to WT (Fig 5C). Notably, this decrease in pCaMKII is quantitatively similar to the roughly 33% loss of Ca^2+^-independent CaMKII activity indicated by Krucker *et al*. [127]. Further, our competitive model results are also consistent with Pak *et al*., who report a 40% decrease in pCaMKII in KO Ng^-/-^ mice compared to WT (Ng^+/+^) mice [126].

Because our model does not allow for either spatial effects or variations in free Ca^2+^ concentration, these results suggest that competition for CaM alone could explain the paradoxical effect of Ng genetic knockout on CaMKII autophosphorylation and activity. pCaMKII levels seem to be regulated, at least in-part, by the competition for CaM established by Ng. With Ng, an abundance of the CaM not bound to Ng preferentially binds CaMKII (at moderate Ca^2+^ levels) because CaMKII can out-compete the other candidate binding partners. Without Ng, as in Ng^-/-^ knockout mice, this competitive advantage of CaMKII to bind CaM is reduced, likely because the CaM that would normally bind Ng instead binds other proteins that do not dissociate as readily when high levels of Ca^2+^ are introduced.

This interpretation predicts that the decreased CaMKII autophosphorylation and activity seen in the Ng^-/-^ knockouts occurs as a result of increased CaM-binding to other partners. To identify which other partners most preferentially bind CaM upon decreasing Ng, we first employ “semi-isolated” models containing only Ng and one of the seven other CaM binding partners (Fig 5D). Semi-isolated models are utilized in Fig 5D to help ensure that shifts in binding partner activation with decreasing Ng are in fact due to decreasing Ng. The partners that experience the greatest relative increase in CaM-binding as Ng concentration is decreased are AC8-Ct and AC8-Nt (calculated according the average CaM bound concentration, C_b_, in Eq 1). A more pronounced increase in the average bound concentration of AC8-Ct and AC8-Nt is seen in full competitive model simulations at decreasing Ng concentrations (Fig 5E). This could indicate that the decrease in CaMKII autophosphorylation and activity in Ng^-/-^ mice is due to the shift in availability of (that is, the competition for) CaM due to its increased binding to AC8 during high frequency stimulation. To investigate this, the average bound concentrations of AC8-Ct and AC8-Nt are summed together into AC8 in Fig 5E and plotted along with the average bound concentration of CaMKII as a function of initial Ng concentration for both isolated and competitive model simulations. CaM-binding to AC8 appears sufficient to explain these changes, with the amount of increase in the average bound AC8 concentration at decreasing Ng concentration closely mirroring the decrease in the average bound CaMKII concentration.

## Discussion

In the present study, we use a system of ordinary differential equations to model the dynamic interactions of Ca^2+^, CaM, and seven CaM target proteins implicated in LTP and LTD of hippocampal synapses. By developing both “isolated” and “competitive” models of this system, we observe competition among these target proteins for CaM-binding and investigate competition’s role in regulating the frequency-dependent activation of downstream CaM binding proteins. The dynamic behavior of our model is largely determined by kinetic rate constants that describe the binding of CaM to Ca^2+^ and CaM binding to downstream binding to CaM binding proteins. Our models are parameterized using published values where available, and are calculated by applying experimentally supported assumptions and the thermodynamic principle of microscopic reversibility. Global sensitivity analyses are performed to determine the impact of these assumptions on our conclusions, and we find that very few of the parameters that significantly impacted our results are derived from these assumptions.

One of the major results of this work is that competitive binding could be among the mechanisms by which protein activation is dynamically tuned and regulated. We find that the presence of competitors affects not only the concentration of all respective CaM-bound proteins, but also the CaM-binding dynamics of these targets. Based on the results of the present work, we recommend at least the inclusion of Ng into models simulating the activation of CaM-dependent proteins in response to low frequency Ca^2+^ transients and the inclusion of CaMKII into models simulating the activation of CaM-dependent proteins in response to high frequency Ca^2+^ transients. Based on the results of our global sensitivity analyses, these two proteins appear to have the most significant impact on the CaM-binding of other CaM targets at these frequency ranges.

Another major result of this work is that competitive tuning may be able to explain the counter-intuitive results from studies of Ng knockouts in mice (Ng^-/-^) in which CaMKII autophosphorylation and activity levels were seen to decrease in the Ng^-/-^ compared to WT. Our results suggest that under tetanic stimulation and normal initial Ng concentration, Ng buffers CaM from AC8 but not CaMKII. At low concentrations or in the absence of Ng, AC and particularly AC-Ct, is able to bind more CaM, while CaMKII binds less CaM (Fig 5F). Although the K_D_ value of CaM_4_ binding to CaMKII and AC-Ct are only within 2-fold of each other (1.7 μM and 0.8 μM, respectively), they exhibit very different binding dynamics based on their binding of sub-saturated CaM (CaM_2C_ and CaM_2N_). This is best seen in Fig 3. For AC-Ct, the dominant species of CaM that binds is CaM_2N_, making up greater than 50% of the total CaM species bound to AC-Ct. In contrast, for CaMKII there is no dominant species of CaM that binds; CaM_2N_ and CaM_4_ are major contributors to the total CaMKII-CaM bound species. The binding dynamics of CaM-CaMKII interactions that are seen in the competitive model suggest, as previous work has suggested [39], that CaMKII binds to CaM_2C_ and this CaM_2C_ is then converted to CaM_4_ while still bound to CaMKII, as noted by the coincident decline in CaMKII-CaM_2_ and increase in CaMKII-CaM_4_ in Fig 3 and S3 Fig in S1 Appendix. The binding dynamics of AC8-Ct seem to indicate that AC8-Ct binds CaM_2N_ and stays bound until the next Ca^2+^ spike. Thus, we hypothesize that AC8-Nt is able to out compete CaMKII for CaM binding in absence of Ng because of its relatively high affinity for CaM_2N_. Since the dynamic behavior that we see in the competitive model is so dependent on the rate parameters it would be ideal if more of them could be experimentally determined in the future. To test the hypothesis that AC8 activity would be increased in a Ng^-/-^ model, we suggest an experiment in which cAMP production is measured in CA1 hippocampal slices from Ng^+/+^ and Ng^-/-^ mice while employing forskolin and specific AC1 blockers to control for cAMP production by AC1 and G protein activation, respectively. If our proposed model is accurate, then increased cAMP production will be observed in Ng^-/-^ mice.

Protein networks for which the initiating ligand is a limiting resource, such as the Ca^2+^/CaM network studied here, are common in biology. As *in vivo* ligand concentrations often approach the dissociation constants of their binding partners, the concentration of bound ligand could exceed that of free ligand, resulting in the phenomenon of ligand depletion [128]. Ligand depletion, as described by Edelstein *et al*., reduces cooperative interactions and broadens the range of signals to which the ligand is most responsive. It may be that we observe ligand depletion phenomena in our isolated models (Fig 4A), given the broad range of Ca^2+^ frequencies at which many binding partners are activated, especially for AC8 and MLCK. However, if ligand depletion really were the predominant regulatory phenomenon, we would expect that by introducing more binding partners (Fig 4B), the broadening effect of ligand depletion would become more conspicuous. Instead, we see a shift and narrowing of the Ca^2+^ frequencies over which the binding partners are activated. Thus, we are confident that it is competition among the CaM-binding proteins that is the mechanism underlying this tuning behavior.

Because competition seems to be important in our neuron-based model, we sought to compare our results to a different biological system with Ca^2+^/CaM-dependent signaling. The 2008 publication by Saucerman and Bers examines activation of CaMKII and CaN in a compartmentalized model of cardiomyocytes, stimulated at Ca^2+^ frequencies ranging from 0–4 Hz [52, 53]. Although this frequency range is much narrower than that used in our competitive model, we can still compare trends of frequency-dependent protein activation. For example, CaMKII activation increases with frequency for both models. Additionally, our isolated model agrees with the Saucerman-Bers model without CaM buffers, in which CaN activation dramatically increases over 0-4Hz. In our competitive model CaN activation is attenuated, in agreement with the Saucerman-Bers model with CaM buffers. This agreement lends further confidence to our model, as the Saucerman-Bers results were subsequently verified experimentally [129]. It appears our model using explicitly-defined CaM buffers (binding proteins) is consistent with the Saucerman-Bers implementation of generalized, unidentified CaM buffers.

The 2008 model by Saucerman and Bers, though not explicitly spatial, highlights how protein localization may affect model output. In Saucerman’s model, Ca^2+^ frequency-dependent activation levels are different for the cytosolic and membrane-localized (dyadic) CaN sub-populations. Our current model excludes spatial effects in order to scrutinize competitive binding in the absence of confounding factors. However, we acknowledge that spatial effects likely alter competition for CaM, especially in the PSD. Future work would investigate the effect of spatial localization on competition for CaM binding; in particular instantiating membrane-localized proteins such as AC1, AC8, NOS and especially Ng at or near the membrane. Sub-populations of CaN may also be localized to the PSD through binding with scaffolding proteins such as AKAP79 [130–132]. Indeed, because we describe Ng as freely diffusing, it is possible our model exaggerates the ability of Ng to compete for CaM relative to other proteins in our model. Therefore, it would be interesting to assess whether a competitive model accounting for membrane localization can still explain the paradoxical effect of Ng^-/-^ on CaMKII autophosphorylation.

Together, our results suggest that the frequency-dependence of CaM targets observed *in vivo* is not an inherent property of these proteins, but rather may be an emergent property of their competitive environment. This competitive tuning may provide a mechanism by which otherwise-independent protein pathways can engage in crosstalk through the limited availability of CaM. We propose that competitive tuning, alongside binding dynamics, feedback loops, and spatial localization, may serve as a major regulator of CaM target protein activation. Furthermore, we have attempted to explain the paradoxical decrease in CaMKII activity seen in Ng^-/-^ mice as a result of the dysregulation of this competitive tuning mechanism. In the absence of spatial effects or aperiodic variations in free Ca^2+^ concentration, competitive tuning is able to offer an explanation for this phenomenon. It is important to note that other proteins, mechanisms, or pathways not included in this model likely lend robustness and further regulatory mechanisms of this phenomenon. Further, it is unlikely that seven CaM-target proteins studied here are the only CaM target proteins that engage in this type of crosstalk through limiting CaM. If competitive tuning facilitates crosstalk among CaM binding proteins, then genetic disorders, neurological diseases, normal aging processes, and therapeutics that disrupt any one CaM target protein may have non-intuitive effects that extend into other signaling pathways. Computational modeling and analysis will continue to play a large role deciphering these oft counter-intuitive regulatory mechanisms that when disrupted, give rise to complex neurological disorders and other important diseases.

## Methods

### Simulation methods

All numerical integration and data manipulation were performed in Mathematica as described in Model Analysis. Reaction equations were implemented using Mathematica [133] with the XCellerator package [134]. XCellerator uses the Law of Mass Action to create ordinary differential equations describing the time rate of change in concentration for each binding partner and their respective CaM-bound states. In Eq 3 we monitor the concentration of a generalized Ca^2+^/CaM state complexed with an arbitrary binding partner, T_b_:

$$\begin{array}{l} \frac{d\left[T_{b}{\mathrm{CaMN}}_{i}C_{j}\right]}{\mathrm{dt}}\\ \;=k_{\mathrm{on}}^{T_{b}{\mathrm{CaMN}}_{i}C_{j}}\left[T_{b}\right]\left[{\mathrm{CaMN}}_{i}C_{j}\right]-k_{\mathrm{off}}^{T_{b}{\mathrm{CaMN}}_{i}C_{j}}\left[T_{b}{\mathrm{CaMN}}_{i}C_{j}\right]\\ \;+k_{\mathrm{on}}^{T_{b}\mathrm{jC}}\left[{\mathrm{Ca}}^{2+}\right]\left[T_{b}{\mathrm{CaMN}}_{i}C_{j-1}\right]+k_{\mathrm{on}}^{T_{b}\mathrm{iN}}\left[{\mathrm{Ca}}^{2+}\right]\left[T_{b}{\mathrm{CaMN}}_{i-1}C_{j}\right]\\ \;+k_{\mathrm{off}}^{T_{b}\left(i+1\right)N}\left[T_{b}{\mathrm{CaMN}}_{i+1}C_{j}\right]+k_{\mathrm{off}}^{T_{b}\left(i+1\right)C}\left[T_{b}{\mathrm{CaMN}}_{i}C_{j+1}\right]\\ \;-\left[T_{b}{\mathrm{CaMN}}_{i}C_{j}\right]\left(k_{\mathrm{off}}^{T_{b}\mathrm{iN}}+k_{\mathrm{off}}^{T_{b}\mathrm{jC}}+k_{\mathrm{on}}^{T_{b}\left(i+1\right)N}\left[{\mathrm{Ca}}^{2+}\right]+k_{\mathrm{on}}^{T_{b}\left(j+1\right)C}\left[{\mathrm{Ca}}^{2+}\right]\right) \end{array}$$ (3)

where i and j = 0, 1, or 2.

For simulations involving autophosphorylation of CaMKII, we extend the system of differential equations generalized in Eq 3 to describe formation of a complex between two active (CaM-bound) CaMKII monomers (Eq 4). Finally, complexes of CaMKII monomers react such that one monomer behaves as an enzyme and the other becomes the phosphorylated substrate (Eq 5). As stated, we refer directly to the previously-published model of CaMKII autophosphorylation by Pepke *et al*. (see Fig 6 in [39]).

$$\begin{array}{l} \frac{d[{{Dimer}_{CaMKII}N}_{1,i}N_{2,m}C_{1,j}C_{2,n}]}{dt}\\ \;=k_{on}^{Dimer}[CaMKIICaMN_{1,i}C_{1,j}][CaMKIICaMN_{2,m}C_{2,n}]\\ \;-k_{off}^{Dimer}[{{Dimer}_{CaMKII}N}_{1,i}N_{2,m}C_{1,j}C_{2,n}] \end{array}$$ (4)

$$\frac{d[{pCaMKIICaMN}_{i}C_{j}]}{dt}=k_{p}^{CaMN_{i}C_{j}}[{{Dimer}_{CaMKII}N}_{1,i}N_{2,m}C_{1,j}C_{2,n}]$$ (5)

Where i, j, m, and n = 0, 1, or 2. Phosphorylated CaMKII monomers may also be one of the two participating species in Eq 4.

All the equations for this model can be found in S3 Appendix. Mathematica files for the complete models can be found on the Purdue PURR database: Romano, D.; Pharris, M. C.; Patel, N.; Kinzer-Ursem, T. L. (2017), "Mathematica Files: Competitive tuning: competition’s role in setting the frequency-dependence of Ca^2+^-dependent proteins." (DOI: 10.4231/R7154F7Q). Our model code is also being uploaded to the BioModels Database [135–137].

### Sensitivity analysis

Despite our best efforts to constrain our models’ parameter values to those that have been experimentally-measured or those which can be calculated by the principle of thermodynamic equilibrium, it was still a valuable exercise to investigate the effects of the previously-described calculations and assumptions on model conclusions. Therefore, a global sensitivity analysis was used to investigate how uncertainty in parameter values impacted model outputs. Latin hypercube sampling (LHS) was used to simultaneously sample input parameter spaces, and partial rank correlation coefficients (PRCC) were calculated to measure the correlation between variation in parameter values and variation in model outputs. These methods have been previously described (see [39, 75]). In short, for each CaM target, a uniform probability distribution of input parameter values was assumed to either span the experimental range specified in S1 Table or, if a range of experimental values is not present, 50–200% of experimental, calculated, or assumed values. A perfect positive correlation gave a PRCC of 1, whereas a perfect negative correlation gives a PRCC of -1. A threshold of 0.5 was used to select for only the parameters that significantly impacted (either positively or negatively) the average bound concentration of each binding partner, and parameters were then ranked by the absolute value of their PRCCs. For the sake of completeness, the sensitivity analysis was done for the nine-state model of Ca^2+^-CaM binding. Further discussion and enumeration of our sensitivity analysis is in S2 Appendix.

## Supporting information

S1 Table Model parameter description and values. Refer to the Model Parameterization sub-section for explanation of parameters and justification of our model assumptions.
(PDF)

S1 Appendix Includes a discussion and comparison of 4-state and 9-state models of CaM-protein binding. Detailed explanation of the differences between these two model types.
(PDF)

S2 Appendix Discussion and results of sensitivity analysis. Partial rank correlation coefficients for all initial concentrations and kinetic parameters and their influence on the average bound concentration of CaM to each of its downstream binding partners included in this study.
(PDF)

S3 Appendix Equations for four-state model, generated by the Mathematica module XCellerator. (PDF)
