# Supplementary material for: Competitive tuning: Competition's role in setting the frequency-dependence of Ca2+-dependent proteins
Source: PLoS Comput Biol. 2017 Nov 6;13(11):e1005820. doi: 10.1371/journal.pcbi.1005820 (PMC5690689; doi:10.1371/journal.pcbi.1005820)
Supplement: S1 Table — Refer to the Model Parameterization sub-section for explanation of parameters and justification of our model assumptions. (PDF) [file pcbi.1005820.s001.pdf]

Parameter	Description	Value Range	Value Used	Reference(s)
Ca^{2+} binding to CaM				
k_{on}^{1N}	1st Ca^{2+} binding to CaM N-terminus	25.0-260.0 $\mu M^{-1}s^{-1}$	100.0 $\mu M^{-1}s^{-1}$	[1]
k_{off}^{1N}	1st Ca^{2+} dissociation from CaM N-terminus	1000.0-4000.0 s^{-1}	2500.0 s^{-1}	[1]
K_{D}^{1N}	Equilibrium binding of 1st Ca^{2+} to CaM N-terminus	15.0-40.0 μM	25.0 μM	[1]
k_{on}^{2N}	2nd Ca^{2+} binding to CaM N-terminus	50.0-300.0 $\mu M^{-1}s^{-1}$	150.0 $\mu M^{-1}s^{-1}$	[1]
k_{off}^{2N}	2nd Ca^{2+} dissociation from CaM N-terminus	500.0-1000.0+ s^{-1}	750.0 s^{-1}	[1]
K_{D}^{2N}	Equilibrium binding of 2nd Ca^{2+} to CaM N-terminus	3.3-9.0 μM	5.0 μM	[1]
k_{on}^{1C}	1st Ca^{2+} binding to CaM C-terminus	1.2-9.6 $\mu M^{-1}s^{-1}$	4.0 $\mu M^{-1}s^{-1}$	[1]
k_{off}^{1C}	1st Ca^{2+} dissociation from CaM C-terminus	10.0-70.0 s^{-1}	40.0 s^{-1}	[1]
K_{D}^{1C}	Equilibrium binding of 1st Ca^{2+} to CaM C-terminus	7.3-12.0 μM	10.0 μM	[1]
k_{on}^{2C}	2nd Ca^{2+} binding to CaM C-terminus	5.0-25.0 $\mu M^{-1}s^{-1}$	10.0 $\mu M^{-1}s^{-1}$	[1]
k_{off}^{2C}	2nd Ca^{2+} dissociation from CaM C-terminus	8.5-10.0 s^{-1}	9.25 s^{-1}	[1]
K_{D}^{2C}	Equilibrium binding of 2nd Ca^{2+} to CaM C-terminus	0.4-1.7 μM	0.925 μM	[1]
CaM binding to AC1				
$k_{on}^{AC1CaM0}$	CaM0 binding to AC1		0.00166 $\mu M^{-1}s^{-1}$	[2]
$k_{off}^{AC1CaM0}$	CaM0 dissociation from AC1		0.9 s^{-1}	[3]
$K_{D}^{AC1CaM0}$	Equilibrium binding of CaM0 to AC1		542.0 μM	[4]
$k_{on}^{AC1CaM1N}$	CaM1N binding to AC1		0.00166 $\mu M^{-1}s^{-1}$	[2]
$k_{off}^{AC1CaM1N}$	CaM1N dissociation from AC1		0.9 s^{-1}	[3]
$K_{D}^{AC1CaM1N}$	Equilibrium binding of CaM1N to AC1		542.0 μM	[4]
$k_{on}^{AC1CaM1C}$	CaM1C binding to AC1		0.00830 $\mu M^{-1}s^{-1}$	[2]
$k_{off}^{AC1CaM1C}$	CaM1C dissociation from AC1		0.9 s^{-1}	[3]
$K_{D}^{AC1CaM1C}$	Equilibrium binding of CaM1C to AC1		108.0 μM	[4]
$k_{on}^{AC1CaM1N1C}$	CaM1N1C binding to AC1		0.00830 $\mu M^{-1}s^{-1}$	[2]
$k_{off}^{AC1CaM1N1C}$	CaM1N1C dissociation from AC1		0.9 s^{-1}	[3]
$K_{D}^{AC1CaM1N1C}$	Equilibrium binding of CaM1N1C to AC1		108.0 μM	[4]
$k_{on}^{AC1CaM2N}$	CaM2N binding to AC1		0.156 $\mu M^{-1}s^{-1}$	[2]
$k_{off}^{AC1CaM2N}$	CaM2N dissociation from AC1		0.9 s^{-1}	[3]
$K_{D}^{AC1CaM2N}$	Equilibrium binding of CaM2N to AC1		5.78 μM	[4]
$k_{on}^{AC1CaM2C}$	CaM2C binding to AC1		0.064 $\mu M^{-1}s^{-1}$	[2]
$k_{off}^{AC1CaM2C}$	CaM2C dissociation from AC1		0.9 s^{-1}	[3]
$K_{D}^{AC1CaM2C}$	Equilibrium binding of CaM2C to AC1		14.1 μM	[4]
$k_{on}^{AC1CaM2N1C}$	CaM2N1C binding to AC1		0.778 $\mu M^{-1}s^{-1}$	[2]
$k_{off}^{AC1CaM2N1C}$	CaM2N1C dissociation from AC1		0.9 s^{-1}	[3]
$K_{D}^{AC1CaM2N1C}$	Equilibrium binding of CaM2N1C to AC1		1.16 μM	[4]
$k_{on}^{AC1CaM1N2C}$	CaM1N2C binding to AC1		0.064 $\mu M^{-1}s^{-1}$	[2]

$k_{off}^{AC1CaM1N2C}$	CaM1N2C dissociation from AC1		0.9 s^{-1}	[3]
$K_{D}^{AC1CaM1N2C}$	Equilibrium binding of CaM1N2C to AC1		14.1 μM	[4]
$k_{on}^{AC1CaM4}$	CaM4 binding to AC1		6.0 $\mu M^{-1}s^{-1}$	[5]
$k_{off}^{AC1CaM4}$	CaM4 dissociation from AC1		0.9 s^{-1}	[5]
$K_{D}^{AC1CaM4}$	Equilibrium binding of CaM4 to AC1		0.15 μM	[2]
Ca^{2+} binding to AC1-CaM				
k_{on}^{AC11N}	1st Ca^{2+} binding to AC1-CaM N-terminus		100.0 $\mu M^{-1}s^{-1}$	[6]
k_{off}^{AC11N}	1st Ca^{2+} dissociation from AC1-CaM N-terminus		2500.0 s^{-1}	[8]
K_{D}^{AC11N}	Equilibrium binding of 1st Ca^{2+} to AC1-CaM N-terminus		25.0 μM	[2]
k_{on}^{AC12N}	2nd Ca^{2+} binding to AC1-CaM N-terminus		150.0 $\mu M^{-1}s^{-1}$	[6]
k_{off}^{AC12N}	2nd Ca^{2+} dissociation from AC1-CaM N-terminus		8.0 s^{-1}	[8]
K_{D}^{AC12N}	Equilibrium binding of 2nd Ca^{2+} to AC1-CaM N-terminus		0.0533 μM	[2]
k_{on}^{AC11C}	1st Ca^{2+} binding to AC1-CaM C-terminus		4.0 $\mu M^{-1}s^{-1}$	[6]
k_{off}^{AC11C}	1st Ca^{2+} dissociation from AC1-CaM C-terminus		8.0 s^{-1}	[8]
K_{D}^{AC11C}	Equilibrium binding of 1st Ca^{2+} to AC1-CaM C-terminus		2.0 μM	[2]
k_{on}^{AC12C}	2nd Ca^{2+} binding to AC1-CaM C-terminus		10.0 $\mu M^{-1}s^{-1}$	[6]
k_{off}^{AC12C}	2nd Ca^{2+} dissociation from AC1-CaM C-terminus		1.2 s^{-1}	[8]
K_{D}^{AC12C}	Equilibrium binding of 2nd Ca^{2+} to AC1-CaM C-terminus		0.12 μM	[2]
CaM binding to AC8 N-terminus				
$k_{on}^{AC8ntCaM0}$	CaM0 binding to AC8 N-terminus		0.00828 $\mu M^{-1}s^{-1}$	[2]
$k_{off}^{AC8ntCaM0}$	CaM0 dissociation from AC8 N-terminus		1.0 s^{-1}	[3]
$K_{D}^{AC8ntCaM0}$	Equilibrium binding of CaM0 to AC8 N-terminus		121.0 μM	[4]
$k_{on}^{AC8ntCaM1N}$	CaM1N binding to AC8 N-terminus		0.00828 $\mu M^{-1}s^{-1}$	[2]
$k_{off}^{AC8ntCaM1N}$	CaM1N dissociation from AC8 N-terminus		1.0 s^{-1}	[3]
$K_{D}^{AC8ntCaM1N}$	Equilibrium binding of CaM1N to AC8 N-terminus		121.0 μM	[4]
$k_{on}^{AC8ntCaM1C}$	CaM1C binding to AC8 N-terminus		0.0676 $\mu M^{-1}s^{-1}$	[2]
$k_{off}^{AC8ntCaM1C}$	CaM1C dissociation from AC8 N-terminus		1.0 s^{-1}	[3]
$K_{D}^{AC8ntCaM1C}$	Equilibrium binding of CaM1C to AC8 N-terminus		14.8 μM	[4]
$k_{on}^{AC8ntCaM1N1C}$	CaM1N1C binding to AC8 N-terminus		0.0676 $\mu M^{-1}s^{-1}$	[2]
$k_{off}^{AC8ntCaM1N1C}$	CaM1N1C dissociation from AC8 N-terminus		1.0 s^{-1}	[3]
$K_{D}^{AC8ntCaM1N1C}$	Equilibrium binding of CaM1N1C to AC8 N-terminus		14.8 μM	[4]

$k_{on}^{AC8ntCaM2N}$	CaM2N binding to AC8 N-terminus		0.00828 $\mu M^{-1}s^{-1}$	[2]
$k_{off}^{AC8ntCaM2N}$	CaM2N dissociation from AC8 N-terminus		1.0 s^{-1}	[3]
$K_{D}^{AC8ntCaM2N}$	Equilibrium binding of CaM2N to AC8 N-terminus		121.0 μM	[4]
$k_{on}^{AC8ntCaM2C}$	CaM2C binding to AC8 N-terminus		1.25 $\mu M^{-1}s^{-1}$	[2]
$k_{off}^{AC8ntCaM2C}$	CaM2C dissociation from AC8 N-terminus		1.0 s^{-1}	[3]
$K_{D}^{AC8ntCaM2C}$	Equilibrium binding of CaM2C to AC8 N-terminus		0.8 μM	[4]
$k_{on}^{AC8ntCaM2N1C}$	CaM2N1C binding to AC8 N-terminus		0.0676 $\mu M^{-1}s^{-1}$	[2]
$k_{off}^{AC8ntCaM2N1C}$	CaM2N1C dissociation from AC8 N-terminus		1.0 s^{-1}	[3]
$K_{D}^{AC8ntCaM2N1C}$	Equilibrium binding of CaM2N1C to AC8 N-terminus		14.8 μM	[4]
$k_{on}^{AC8ntCaM1N2C}$	CaM1N2C binding to AC8 N-terminus		1.25 $\mu M^{-1}s^{-1}$	[2]
$k_{off}^{AC8ntCaM1N2C}$	CaM1N2C dissociation from AC8 N-terminus		1.0 s^{-1}	[3]
$K_{D}^{AC8ntCaM1N2C}$	Equilibrium binding of CaM1N2C to AC8 N-terminus		0.8 μM	[4]
$k_{on}^{AC8ntCaM4}$	CaM4 binding to AC8 N-terminus		1.25 $\mu M^{-1}s^{-1}$	[5]
$k_{off}^{AC8ntCaM4}$	CaM4 dissociation from AC8 N-terminus		1.0 s^{-1}	[5]
$K_{D}^{AC8ntCaM4}$	Equilibrium binding of CaM4 to AC8 N-terminus		0.8 μM	[2]
Ca^{2+} binding to AC8(N-terminus)-CaM				
$k_{on}^{AC8nt1N}$	1st Ca^{2+} binding to AC8(N-terminus)-CaM N-terminus		100.0 $\mu M^{-1}s^{-1}$	[6]
$k_{off}^{AC8nt1N}$	1st Ca^{2+} dissociation from AC8(N-terminus)-CaM N-terminus		2500.0 s^{-1}	[7]
$K_{D}^{AC8nt1N}$	Equilibrium binding of 1st Ca^{2+} to AC8(N-terminus)-CaM N-terminus		25.0 μM	[2]
$k_{on}^{AC8nt2N}$	2nd Ca^{2+} binding to AC8(N-terminus)-CaM N-terminus		150.0 $\mu M^{-1}s^{-1}$	[6]
$k_{off}^{AC8nt2N}$	2nd Ca^{2+} dissociation from AC8(N-terminus)-CaM N-terminus		750.0 s^{-1}	[8]
$K_{D}^{AC8nt2N}$	Equilibrium binding of 2nd Ca^{2+} to AC8(N-terminus)-CaM N-terminus		5.0 μM	[2]
$k_{on}^{AC8nt1C}$	1st Ca^{2+} binding to AC8(N-terminus)-CaM C-terminus		4.0 $\mu M^{-1}s^{-1}$	[6]
$k_{off}^{AC8nt1C}$	1st Ca^{2+} dissociation from AC8(N-terminus)-CaM C-terminus		4.9 s^{-1}	[8]
$K_{D}^{AC8nt1C}$	Equilibrium binding of 1st Ca^{2+} to AC8(N-terminus)-CaM C-terminus		1.23 μM	[2]
$k_{on}^{AC8nt2C}$	2nd Ca^{2+} binding to AC8(N-terminus)-CaM C-terminus		10.0 $\mu M^{-1}s^{-1}$	[6]
$k_{off}^{AC8nt2C}$	2nd Ca^{2+} dissociation from AC8(N-terminus)-CaM C-terminus		0.5 s^{-1}	[8]

$K_D^{AC8nt2C}$	Equilibrium binding of 2nd Ca^{2+} to AC8(N-terminus)-CaM C-terminus		0.05 μM	[2]
CaM binding to AC8 C-terminus				
$k_{on}^{AC8ctCaM0}$	CaM0 binding to AC8 C-terminus		0.00267 $\mu M^{-1}s^{-1}$	[2]
$k_{off}^{AC8ctCaM0}$	CaM0 dissociation from AC8 C-terminus		1.0 s^{-1}	[3]
$K_D^{AC8ctCaM0}$	Equilibrium binding of CaM0 to AC8 C-terminus		375.0 μM	[4]
$k_{on}^{AC8ctCaM1N}$	CaM1N binding to AC8 C-terminus		0.00267 $\mu M^{-1}s^{-1}$	[2]
$k_{off}^{AC8ctCaM1N}$	CaM1N dissociation from AC8 C-terminus		1.0 s^{-1}	[3]
$K_D^{AC8ctCaM1N}$	Equilibrium binding of CaM1N to AC8 C-terminus		375.0 μM	[4]
$k_{on}^{AC8ctCaM1C}$	CaM1C binding to AC8 C-terminus		0.00267 $\mu M^{-1}s^{-1}$	[2]
$k_{off}^{AC8ctCaM1C}$	CaM1C dissociation from AC8 C-terminus		1.0 s^{-1}	[3]
$K_D^{AC8ctCaM1C}$	Equilibrium binding of CaM1C to AC8 C-terminus		375.0 μM	[4]
$k_{on}^{AC8ctCaM1N1C}$	CaM1N1C binding to AC8 C-terminus		0.00267 $\mu M^{-1}s^{-1}$	[2]
$k_{off}^{AC8ctCaM1N1C}$	CaM1N1C dissociation from AC8 C-terminus		1.0 s^{-1}	[3]
$K_D^{AC8ctCaM1N1C}$	Equilibrium binding of CaM1N1C to AC8 C-terminus		375.0 μM	[4]
$k_{on}^{AC8ctCaM2N}$	CaM2N binding to AC8 C-terminus		1.25 $\mu M^{-1}s^{-1}$	[2]
$k_{off}^{AC8ctCaM2N}$	CaM2N dissociation from AC8 C-terminus		1.0 s^{-1}	[3]
$K_D^{AC8ctCaM2N}$	Equilibrium binding of CaM2N to AC8 C-terminus		0.8 μM	[4]
$k_{on}^{AC8ctCaM2C}$	CaM2C binding to AC8 C-terminus		0.00267 $\mu M^{-1}s^{-1}$	[2]
$k_{off}^{AC8ctCaM2C}$	CaM2C dissociation from AC8 C-terminus		1.0 s^{-1}	[3]
$K_D^{AC8ctCaM2C}$	Equilibrium binding of CaM2C to AC8 C-terminus		375.0 μM	[4]
$k_{on}^{AC8ctCaM2N1C}$	CaM2N1C binding to AC8 C-terminus		1.25 $\mu M^{-1}s^{-1}$	[2]
$k_{off}^{AC8ctCaM2N1C}$	CaM2N1C dissociation from AC8 C-terminus		1.0 s^{-1}	[3]
$K_D^{AC8ctCaM2N1C}$	Equilibrium binding of CaM2N1C to AC8 C-terminus		0.8 μM	[4]
$k_{on}^{AC8ctCaM1N2C}$	CaM1N2C binding to AC8 C-terminus		0.00267 $\mu M^{-1}s^{-1}$	[2]
$k_{off}^{AC8ctCaM1N2C}$	CaM1N2C dissociation from AC8 C-terminus		1.0 s^{-1}	[3]
$K_D^{AC8ctCaM1N2C}$	Equilibrium binding of CaM1N2C to AC8 C-terminus		375.0 μM	[4]
$k_{on}^{AC8ctCaM4}$	CaM4 binding to AC8 C-terminus		1.25 $\mu M^{-1}s^{-1}$	[5]
$k_{off}^{AC8ctCaM4}$	CaM4 dissociation from AC8 C-terminus		1.0 s^{-1}	[5]
$K_D^{AC8ctCaM4}$	Equilibrium binding of CaM4 to AC8 C-terminus		0.8 μM	[2]
Ca^{2+} binding to AC8(C-terminus)-CaM				
$k_{on}^{AC8ct1N}$	1st Ca^{2+} binding to AC8(C-terminus)-CaM N-terminus		100.0 $\mu M^{-1}s^{-1}$	[6]

$k_{off}^{AC8ct1N}$	1st Ca^{2+} dissociation from AC8(C-terminus)-CaM N-terminus		2500.0 s^{-1}	[7]
$K_D^{AC8ct1N}$	Equilibrium binding of 1st Ca^{2+} to AC8(C-terminus)-CaM N-terminus		25.0 μM	[2]
$k_{on}^{AC8ct2N}$	2nd Ca^{2+} binding to AC8(C-terminus)-CaM N-terminus		150.0 $\mu M^{-1}s^{-1}$	[6]
$k_{off}^{AC8ct2N}$	2nd Ca^{2+} dissociation from AC8(C-terminus)-CaM N-terminus		1.6 s^{-1}	[8]
$K_D^{AC8ct2N}$	Equilibrium binding of 2nd Ca^{2+} to AC8(C-terminus)-CaM N-terminus		0.0107 μM	[2]
$k_{on}^{AC8ct1C}$	1st Ca^{2+} binding to AC8(C-terminus)-CaM C-terminus		4.0 $\mu M^{-1}s^{-1}$	[6]
$k_{off}^{AC8ct1C}$	1st Ca^{2+} dissociation from AC8(C-terminus)-CaM C-terminus		40.0 s^{-1}	[8]
$K_D^{AC8ct1C}$	Equilibrium binding of 1st Ca^{2+} to AC8(C-terminus)-CaM C-terminus		10.0 μM	[2]
$k_{on}^{AC8ct2C}$	2nd Ca^{2+} binding to AC8(C-terminus)-CaM C-terminus		10.0 $\mu M^{-1}s^{-1}$	[6]
$k_{off}^{AC8ct2C}$	2nd Ca^{2+} dissociation from AC8(C-terminus)-CaM C-terminus		9.25 s^{-1}	[8]
$K_D^{AC8ct2C}$	Equilibrium binding of 2nd Ca^{2+} to AC8(C-terminus)-CaM C-terminus		0.925 μM	[2]
CaM binding to CaN				
k_{on}^{PPCaM0}	CaM0 binding to CaN		0.0000000798 $\mu M^{-1}s^{-1}$	[2]
k_{off}^{PPCaM0}	CaM0 dissociation from CaN		0.000319 s^{-1}	[3]
K_D^{PPCaM0}	Equilibrium binding of CaM0 to CaN		3999.0 μM	[4]
$k_{on}^{PPCaM1N}$	CaM1N binding to CaN		0.00000665 $\mu M^{-1}s^{-1}$	[2]
$k_{off}^{PPCaM1N}$	CaM1N dissociation from CaN		0.000319 s^{-1}	[3]
$K_D^{PPCaM1N}$	Equilibrium binding of CaM1N to CaN		48.0 μM	[4]
$k_{on}^{PPCaM1C}$	CaM1C binding to CaN		0.00001334 $\mu M^{-1}s^{-1}$	[2]
$k_{off}^{PPCaM1C}$	CaM1C dissociation from CaN		0.000319 s^{-1}	[3]
$K_D^{PPCaM1C}$	Equilibrium binding of CaM1C to CaN		23.9 μM	[4]
$k_{on}^{PPCaM1N1C}$	CaM1N1C binding to CaN		0.000665 $\mu M^{-1}s^{-1}$	[2]
$k_{off}^{PPCaM1N1C}$	CaM1N1C dissociation from CaN		0.000319 s^{-1}	[3]
$K_D^{PPCaM1N1C}$	Equilibrium binding of CaM1N1C to CaN		0.480 μM	[4]
$k_{on}^{PPCaM2N}$	CaM2N binding to CaN		0.000416 $\mu M^{-1}s^{-1}$	[2]
$k_{off}^{PPCaM2N}$	CaM2N dissociation from CaN		0.000319 s^{-1}	[3]
$K_D^{PPCaM2N}$	Equilibrium binding of CaM2N to CaN		0.768 μM	[4]
$k_{on}^{PPCaM2C}$	CaM2C binding to CaN		0.000123 $\mu M^{-1}s^{-1}$	[2]
$k_{off}^{PPCaM2C}$	CaM2C dissociation from CaN		0.000319 s^{-1}	[3]
$K_D^{PPCaM2C}$	Equilibrium binding of CaM2C to CaN		2.59 μM	[4]
$k_{on}^{PPCaM2N1C}$	CaM2N1C binding to CaN		0.0416 $\mu M^{-1}s^{-1}$	[2]
$k_{off}^{PPCaM2N1C}$	CaM2N1C dissociation from CaN		0.000319 s^{-1}	[3]

$K_D^{PPCaM2N1C}$	Equilibrium binding of CaM2N1C to CaN		0.00768 μM	[4]
$k_{on}^{PPCaM1N2C}$	CaM1N2C binding to CaN		0.0102 $\mu M^{-1}s^{-1}$	[2]
$k_{off}^{PPCaM1N2C}$	CaM1N2C dissociation from CaN		0.000319 s^{-1}	[3]
$K_D^{PPCaM1N2C}$	Equilibrium binding of CaM1N2C to CaN		0.0311 μM	[4]
k_{on}^{PPCaM4}	CaM4 binding to CaN	0.0089-46.0 $\mu M^{-1}s^{-1}$	0.64 $\mu M^{-1}s^{-1}$	[9-11]
k_{off}^{PPCaM4}	CaM4 dissociation from CaN	0.000085-0.0039 s^{-1}	0.000319 s^{-1}	[9-11]
K_D^{PPCaM4}	Equilibrium binding of CaM4 to CaN	0.000028-0.024 μM	0.000498 μM	[9-11]
Ca^{2+} binding to CaN-CaM				
k_{on}^{PP1N}	1st Ca^{2+} binding to CaN-CaM N-terminus		100.0 $\mu M^{-1}s^{-1}$	[6]
k_{off}^{PP1N}	1st Ca^{2+} dissociation from CaN-CaM N-terminus		30.0 s^{-1}	[2]
K_D^{PP1N}	Equilibrium binding of 1st Ca^{2+} to CaN-CaM N-terminus		0.3 μM	[12]
k_{on}^{PP2N}	2nd Ca^{2+} binding to CaN-CaM N-terminus		150.0 $\mu M^{-1}s^{-1}$	[6]
k_{off}^{PP2N}	2nd Ca^{2+} dissociation from CaN-CaM N-terminus		12.0 s^{-1}	[2]
K_D^{PP2N}	Equilibrium binding of 2nd Ca^{2+} to CaN-CaM N-terminus		0.08 μM	[12]
k_{on}^{PP1C}	1st Ca^{2+} binding to CaN-CaM C-terminus		4.0 $\mu M^{-1}s^{-1}$	[6]
k_{off}^{PP1C}	1st Ca^{2+} dissociation from CaN-CaM C-terminus		0.4 s^{-1}	[2]
K_D^{PP1C}	Equilibrium binding of 1st Ca^{2+} to CaN-CaM C-terminus		0.1 μM	[12]
k_{on}^{PP2C}	2nd Ca^{2+} binding to CaN-CaM C-terminus		10.0 $\mu M^{-1}s^{-1}$	[6]
k_{off}^{PP2C}	2nd Ca^{2+} dissociation from CaN-CaM C-terminus		0.6 s^{-1}	[2]
K_D^{PP2C}	Equilibrium binding of 2nd Ca^{2+} to CaN-CaM C-terminus		0.06 μM	[12]
CaM binding to CaMKII				
k_{on}^{KCaM0}	CaM0 binding to CaMKII		0.0038 $\mu M^{-1}s^{-1}$	[1]
k_{off}^{KCaM0}	CaM0 dissociation from CaMKII		5.5 s^{-1}	[1]
K_D^{KCaM0}	Equilibrium binding of CaM0 to CaMKII		1.45 mM	[2]
k_{on}^{KCaM1N}	CaM1N binding to CaMKII		0.022 $\mu M^{-1}s^{-1}$	[1]
k_{off}^{KCaM1N}	CaM1N dissociation from CaMKII		3.1 s^{-1}	[1]
K_D^{KCaM1N}	Equilibrium binding of CaM1N to CaMKII		141.0 μM	[2]
k_{on}^{KCaM1C}	CaM1C binding to CaMKII		0.059 $\mu M^{-1}s^{-1}$	[1]
k_{off}^{KCaM1C}	CaM1C dissociation from CaMKII		6.1 s^{-1}	[1]
K_D^{KCaM1C}	Equilibrium binding of CaM1C to CaMKII		103.0 μM	[2]
$k_{on}^{KCaM1N1C}$	CaM1N1C binding to CaMKII		0.33 $\mu M^{-1}s^{-1}$	[1]
$k_{off}^{KCaM1N1C}$	CaM1N1C dissociation from CaMKII		3.4 s^{-1}	[1]
$K_D^{KCaM1N1C}$	Equilibrium binding of CaM1N1C to CaMKII		10.3 μM	[2]
k_{on}^{KCaM2N}	CaM2N binding to CaMKII		0.12 $\mu M^{-1}s^{-1}$	[1]

k_{off}^{KCaM2N}	CaM2N dissociation from CaMKII		1.7 s^{-1}	[1]
K_D^{KCaM2N}	Equilibrium binding of CaM2N to CaMKII	16.5-23.5 μM	14.2 μM	[1]
k_{on}^{KCaM2C}	CaM2C binding to CaMKII		0.92 $\mu M^{-1}s^{-1}$	[1]
k_{off}^{KCaM2C}	CaM2C dissociation from CaMKII		6.8 s^{-1}	[1]
K_D^{KCaM2C}	Equilibrium binding of CaM2C to CaMKII	1.6-8.4 μM	7.39 μM	[1]
$k_{on}^{KCaM2N1C}$	CaM2N1C binding to CaMKII		1.9 $\mu M^{-1}s^{-1}$	[1]
$k_{off}^{KCaM2N1C}$	CaM2N1C dissociation from CaMKII		1.9 s^{-1}	[1]
$K_D^{KCaM2N1C}$	Equilibrium binding of CaM2N1C to CaMKII		1.0 μM	[2]
$k_{on}^{KCaM1N2C}$	CaM1N2C binding to CaMKII		5.2 $\mu M^{-1}s^{-1}$	[1]
$k_{off}^{KCaM1N2C}$	CaM1N2C dissociation from CaMKII		3.8 s^{-1}	[1]
$K_D^{KCaM1N2C}$	Equilibrium binding of CaM1N2C to CaMKII		0.731 μM	[2]
k_{on}^{KCaM4}	CaM4 binding to CaMKII	14.0-60.0 $\mu M^{-1}s^{-1}$	30.0 $\mu M^{-1}s^{-1}$	[1]
k_{off}^{KCaM4}	CaM4 dissociation from CaMKII	1.1-2.3 s^{-1}	1.7 s^{-1}	[1]
K_D^{KCaM4}	Equilibrium binding of CaM4 to CaMKII	0.04-0.08 μM	0.0567 μM	[1]
Ca^{2+} binding to CaMKII-CaM				
k_{on}^{K1N}	1st Ca^{2+} binding to CaMKII-CaM N-terminus		76.0 $\mu M^{-1}s^{-1}$	[1]
k_{off}^{K1N}	1st Ca^{2+} dissociation from CaMKII-CaM N-terminus		300.0 s^{-1}	[1]
K_D^{K1N}	Equilibrium binding of 1st Ca^{2+} to CaMKII-CaM N-terminus		3.95 μM	[2]
k_{on}^{K2N}	2nd Ca^{2+} binding to CaMKII-CaM N-terminus		76.0 $\mu M^{-1}s^{-1}$	[1]
k_{off}^{K2N}	2nd Ca^{2+} dissociation from CaMKII-CaM N-terminus	6.0-60.0 s^{-1}	29.6 s^{-1}	[1]
K_D^{K2N}	Equilibrium binding of 2nd Ca^{2+} to CaMKII-CaM N-terminus		0.39 μM	[2]
k_{on}^{K1C}	1st Ca^{2+} binding to CaMKII-CaM C-terminus		44.0 $\mu M^{-1}s^{-1}$	[1]
k_{off}^{K1C}	1st Ca^{2+} dissociation from CaMKII-CaM C-terminus		33.0 s^{-1}	[1]
K_D^{K1C}	Equilibrium binding of 1st Ca^{2+} to CaMKII-CaM C-terminus		0.75 μM	[2]
k_{on}^{K2C}	2nd Ca^{2+} binding to CaMKII-CaM C-terminus		44.0 $\mu M^{-1}s^{-1}$	[1]
k_{off}^{K2C}	2nd Ca^{2+} dissociation from CaMKII-CaM C-terminus	0.49-4.9 s^{-1}	2.7 s^{-1}	[1]
K_D^{K2C}	Equilibrium binding of 2nd Ca^{2+} to CaMKII-CaM C-terminus		0.0614 μM	[2]
CaM binding to MLCK				
k_{on}^{MKCaM0}	CaM0 binding to MLCK		0.00717 $\mu M^{-1}s^{-1}$	[2]
k_{off}^{MKCaM0}	CaM0 dissociation from MLCK		0.132 s^{-1}	[3]
K_D^{MKCaM0}	Equilibrium binding of CaM0 to MLCK		18.4 μM	[4]
$k_{on}^{MKCaM1N}$	CaM1N binding to MLCK		0.00717 $\mu M^{-1}s^{-1}$	[2]
$k_{off}^{MKCaM1N}$	CaM1N dissociation from MLCK		0.132 s^{-1}	[3]
$K_D^{MKCaM1N}$	Equilibrium binding of CaM1N to MLCK		18.4 μM	[4]

$k_{on}^{MKCaM1C}$	CaM1C binding to MLCK		0.00717 $\mu M^{-1}s^{-1}$	[2]
$k_{off}^{MKCaM1C}$	CaM1C dissociation from MLCK		0.132 s^{-1}	[3]
$K_D^{MKCaM1C}$	Equilibrium binding of CaM1C to MLCK		18.4 μM	[4]
$k_{on}^{MKCaM1N1C}$	CaM1N1C binding to MLCK		0.00717 $\mu M^{-1}s^{-1}$	[2]
$k_{off}^{MKCaM1N1C}$	CaM1N1C dissociation from MLCK		0.132 s^{-1}	[3]
$K_D^{MKCaM1N1C}$	Equilibrium binding of CaM1N1C to MLCK		18.4 μM	[4]
$k_{on}^{MKCaM2N}$	CaM2N binding to MLCK		2.34 $\mu M^{-1}s^{-1}$	[2]
$k_{off}^{MKCaM2N}$	CaM2N dissociation from MLCK		0.132 s^{-1}	[3]
$K_D^{MKCaM2N}$	Equilibrium binding of CaM2N to MLCK		0.0564 μM	[4]
$k_{on}^{MKCaM2C}$	CaM2C binding to MLCK		0.170 $\mu M^{-1}s^{-1}$	[2]
$k_{off}^{MKCaM2C}$	CaM2C dissociation from MLCK		0.132 s^{-1}	[3]
$K_D^{MKCaM2C}$	Equilibrium binding of CaM2C to MLCK		0.776 μM	[4]
$k_{on}^{MKCaM2N1C}$	CaM2N1C binding to MLCK		2.34 $\mu M^{-1}s^{-1}$	[2]
$k_{off}^{MKCaM2N1C}$	CaM2N1C dissociation from MLCK		0.132 s^{-1}	[3]
$K_D^{MKCaM2N1C}$	Equilibrium binding of CaM2N1C to MLCK		0.0564 μM	[4]
$k_{on}^{MKCaM1N2C}$	CaM1N2C binding to MLCK		0.170 $\mu M^{-1}s^{-1}$	[2]
$k_{off}^{MKCaM1N2C}$	CaM1N2C dissociation from MLCK		0.132 s^{-1}	[3]
$K_D^{MKCaM1N2C}$	Equilibrium binding of CaM1N2C to MLCK		0.776 μM	[4]
k_{on}^{MKCaM4}	CaM4 binding to MLCK	28.0-110.0 $\mu M^{-1}s^{-1}$	55.5 $\mu M^{-1}s^{-1}$	[13-15]
k_{off}^{MKCaM4}	CaM4 dissociation from MLCK	0.031-0.56 s^{-1}	0.132 s^{-1}	[13-15]
K_D^{MKCaM4}	Equilibrium binding of CaM4 to MLCK	0.0011-0.22 μM	0.00238 μM	[13-15]
Ca^{2+} binding to MLCK-CaM				
k_{on}^{MK1N}	1st Ca^{2+} binding to MLCK-CaM N-terminus		100.0 $\mu M^{-1}s^{-1}$	[6]
k_{off}^{MK1N}	1st Ca^{2+} dissociation from MLCK-CaM N-terminus		2500.0 s^{-1}	[7]
K_D^{MK1N}	Equilibrium binding of 1st Ca^{2+} to MLCK-CaM N-terminus		25.0 μM	[2]
k_{on}^{MK2N}	2nd Ca^{2+} binding to MLCK-CaM N-terminus		150.0 $\mu M^{-1}s^{-1}$	[6]
k_{off}^{MK2N}	2nd Ca^{2+} dissociation from MLCK-CaM N-terminus	2.3-12.5 s^{-1}	2.3 s^{-1}	[15-18]
K_D^{MK2N}	Equilibrium binding of 2nd Ca^{2+} to MLCK-CaM N-terminus		0.015 μM	[2]
k_{on}^{MK1C}	1st Ca^{2+} binding to MLCK-CaM C-terminus		4.0 $\mu M^{-1}s^{-1}$	[6]
k_{off}^{MK1C}	1st Ca^{2+} dissociation from MLCK-CaM C-terminus		40.0 s^{-1}	[7]
K_D^{MK1C}	Equilibrium binding of 1st Ca^{2+} to MLCK-CaM C-terminus		10.0 μM	[2]
k_{on}^{MK2C}	2nd Ca^{2+} binding to MLCK-CaM C-terminus		10.0 $\mu M^{-1}s^{-1}$	[6]
k_{off}^{MK2C}	2nd Ca^{2+} dissociation from MLCK-CaM C-terminus	0.39-0.7 s^{-1}	0.39 s^{-1}	[15-18]
K_D^{MK2C}	Equilibrium binding of 2nd Ca^{2+} to MLCK-CaM C-terminus		0.039 μM	[2]

CaM binding to Ng				
k_{on}^{NgCaM0}	CaM0 binding to Ng		28.0 $\mu M^{-1}s^{-1}$	[19]
k_{off}^{NgCaM0}	CaM0 dissociation from Ng		36.0 s^{-1}	[19]
K_D^{NgCaM0}	Equilibrium binding of CaM0 to Ng		1.29 μM	[19]
$k_{on}^{NgCaM1N}$	CaM1N binding to Ng		28.0 $\mu M^{-1}s^{-1}$	[19]
$k_{off}^{NgCaM1N}$	CaM1N dissociation from Ng		36.0 s^{-1}	[19]
$K_D^{NgCaM1N}$	Equilibrium binding of CaM1N to Ng		1.29 μM	[19]
$k_{on}^{NgCaM1C}$	CaM1C binding to Ng		23.0 $\mu M^{-1}s^{-1}$	[19]
$k_{off}^{NgCaM1C}$	CaM1C dissociation from Ng		35.0 s^{-1}	[19]
$K_D^{NgCaM1C}$	Equilibrium binding of CaM1C to Ng		1.52 μM	[19]
$k_{on}^{NgCaM1N1C}$	CaM1N1C binding to Ng		23.0 $\mu M^{-1}s^{-1}$	[19]
$k_{off}^{NgCaM1N1C}$	CaM1N1C dissociation from Ng		35.0 s^{-1}	[19]
$K_D^{NgCaM1N1C}$	Equilibrium binding of CaM1N1C to Ng		1.52 μM	[19]
$k_{on}^{NgCaM2N}$	CaM2N binding to Ng		28.0 $\mu M^{-1}s^{-1}$	[19]
$k_{off}^{NgCaM2N}$	CaM2N dissociation from Ng		36.0 s^{-1}	[19]
$K_D^{NgCaM2N}$	Equilibrium binding of CaM2N to Ng		1.29 μM	[19]
$k_{on}^{NgCaM2C}$	CaM2C binding to Ng		2.0 $\mu M^{-1}s^{-1}$	[19]
$k_{off}^{NgCaM2C}$	CaM2C dissociation from Ng		136.0 s^{-1}	[19]
$K_D^{NgCaM2C}$	Equilibrium binding of CaM2C to Ng		68.0 μM	[19]
$k_{on}^{NgCaM2N1C}$	CaM2N1C binding to Ng		23.0 $\mu M^{-1}s^{-1}$	[19]
$k_{off}^{NgCaM2N1C}$	CaM2N1C dissociation from Ng		35.0 s^{-1}	[19]
$K_D^{NgCaM2N1C}$	Equilibrium binding of CaM2N1C to Ng		1.52 μM	[19]
$k_{on}^{NgCaM1N2C}$	CaM1N2C binding to Ng		2.0 $\mu M^{-1}s^{-1}$	[19]
$k_{off}^{NgCaM1N2C}$	CaM1N2C dissociation from Ng		136.0 s^{-1}	[19]
$K_D^{NgCaM1N2C}$	Equilibrium binding of CaM1N2C to Ng		68.0 μM	[19]
k_{on}^{NgCaM4}	CaM4 binding to Ng		2.0 $\mu M^{-1}s^{-1}$	[19]
k_{off}^{NgCaM4}	CaM4 dissociation from Ng		136.0 s^{-1}	[19]
K_D^{NgCaM4}	Equilibrium binding of CaM4 to Ng		68.0 μM	[19]
Ca^{2+} binding to Ng-CaM				
k_{on}^{Ng1N}	1st Ca^{2+} binding to Ng-CaM N-terminus		100.0 $\mu M^{-1}s^{-1}$	[19]
k_{off}^{Ng1N}	1st C a^{2+} dissociation from Ng-CaM N-terminus		2500.0 s^{-1}	[19]
K_D^{Ng1N}	Equilibrium binding of 1st Ca^{2+} to Ng-CaM N-terminus		25.0 μM	[19]
k_{on}^{Ng2N}	2nd Ca^{2+} binding to Ng-CaM N-terminus		150.0 $\mu M^{-1}s^{-1}$	[19]
k_{off}^{Ng2N}	2nd Ca^{2+} dissociation from Ng-CaM N-terminus		750.0 s^{-1}	[19]
K_D^{Ng2N}	Equilibrium binding of 2nd Ca^{2+} to Ng-CaM N-terminus		5.0 μM	[19]
k_{on}^{Ng1C}	1st Ca^{2+} binding to Ng-CaM C-terminus		426.0 $\mu M^{-1}s^{-1}$	[19]
k_{off}^{Ng1C}	1st Ca^{2+} dissociation from Ng-CaM C-terminus		5830.0 s^{-1}	[19]

K_D^{Ng1C}	Equilibrium binding of 1st Ca^{2+} to Ng-CaM C-terminus		13.7 μM	[19]
k_{on}^{Ng2C}	2nd Ca^{2+} binding to Ng-CaM C-terminus		21.5 $\mu M^{-1}s^{-1}$	[19]
k_{off}^{Ng2C}	2nd Ca^{2+} dissociation from Ng-CaM C-terminus		418.0 s^{-1}	[19]
K_D^{Ng2C}	Equilibrium binding of 2nd Ca^{2+} to Ng-CaM C-terminus		19.4 μM	[19]
CaM binding to NOS				
$k_{on}^{NOSCaM0}$	CaM0 binding to NOS		0.135 $\mu M^{-1}s^{-1}$	[2]
$k_{off}^{NOSCaM0}$	CaM0 dissociation from NOS		0.01 s^{-1}	[3]
$K_D^{NOSCaM0}$	Equilibrium binding of CaM0 to NOS		0.074 μM	[4]
$k_{on}^{NOSCaM1N}$	CaM1N binding to NOS		0.135 $\mu M^{-1}s^{-1}$	[2]
$k_{off}^{NOSCaM1N}$	CaM1N dissociation from NOS		0.01 s^{-1}	[3]
$K_D^{NOSCaM1N}$	Equilibrium binding of CaM1N to NOS		0.074 μM	[4]
$k_{on}^{NOSCaM1C}$	CaM1C binding to NOS		0.135 $\mu M^{-1}s^{-1}$	[2]
$k_{off}^{NOSCaM1C}$	CaM1C dissociation from NOS		0.01 s^{-1}	[3]
$K_D^{NOSCaM1C}$	Equilibrium binding of CaM1C to NOS		0.074 μM	[4]
$k_{on}^{NOSCaM1N1C}$	CaM1N1C binding to NOS		0.135 $\mu M^{-1}s^{-1}$	[2]
$k_{off}^{NOSCaM1N1C}$	CaM1N1C dissociation from NOS		0.01 s^{-1}	[3]
$K_D^{NOSCaM1N1C}$	Equilibrium binding of CaM1N1C to NOS		0.074 μM	[4]
$k_{on}^{NOSCaM2N}$	CaM2N binding to NOS		0.135 $\mu M^{-1}s^{-1}$	[2]
$k_{off}^{NOSCaM2N}$	CaM2N dissociation from NOS		0.01 s^{-1}	[3]
$K_D^{NOSCaM2N}$	Equilibrium binding of CaM2N to NOS		0.074 μM	[4]
$k_{on}^{NOSCaM2C}$	CaM2C binding to NOS		1.25 $\mu M^{-1}s^{-1}$	[2]
$k_{off}^{NOSCaM2C}$	CaM2C dissociation from NOS		0.01 s^{-1}	[3]
$K_D^{NOSCaM2C}$	Equilibrium binding of CaM2C to NOS		0.008 μM	[4]
$k_{on}^{NOSCaM2N1C}$	CaM2N1C binding to NOS		0.135 $\mu M^{-1}s^{-1}$	[2]
$k_{off}^{NOSCaM2N1C}$	CaM2N1C dissociation from NOS		0.01 s^{-1}	[3]
$K_D^{NOSCaM2N1C}$	Equilibrium binding of CaM2N1C to NOS		0.074 μM	[4]
$k_{on}^{NOSCaM1N2C}$	CaM1N2C binding to NOS		1.25 $\mu M^{-1}s^{-1}$	[2]
$k_{off}^{NOSCaM1N2C}$	CaM1N2C dissociation from NOS		0.01 s^{-1}	[3]
$K_D^{NOSCaM1N2C}$	Equilibrium binding of CaM1N2C to NOS		0.008 μM	[4]
$k_{on}^{NOSCaM4}$	CaM4 binding to NOS	0.11-660.0 $\mu M^{-1}s^{-1}$	1.25 $\mu M^{-1}s^{-1}$	[20-22]
$k_{off}^{NOSCaM4}$	CaM4 dissociation from NOS	0.0023-3.7 s^{-1}	0.01 s^{-1}	[20-22]
$K_D^{NOSCaM4}$	Equilibrium binding of CaM4 to NOS	0.005-5.6 μM	0.008 μM	[20-24]
Ca^{2+} binding to NOS-CaM				
k_{on}^{NOS1N}	1st Ca^{2+} binding to NOS-CaM N-terminus		100.0 $\mu M^{-1}s^{-1}$	[6]
k_{off}^{NOS1N}	1st Ca^{2+} dissociation from NOS-CaM N-terminus		2500.0 s^{-1}	[25]
K_D^{NOS1N}	Equilibrium binding of 1st Ca^{2+} to NOS-CaM N-terminus		25.0 μM	[2]
k_{on}^{NOS2N}	2nd Ca^{2+} binding to NOS-CaM N-terminus		150.0 $\mu M^{-1}s^{-1}$	[6]

k_{off}^{NOS2N}	2nd Ca^{2+} dissociation from NOS-CaM N-terminus		750.0 s^{-1}	[25]
K_D^{NOS2N}	Equilibrium binding of 2nd Ca^{2+} to NOS-CaM N-terminus		5.0 μM	[2]
k_{on}^{NOS1C}	1st Ca^{2+} binding to NOS-CaM C-terminus		4.0 $\mu M^{-1}s^{-1}$	[6]
k_{off}^{NOS1C}	1st Ca^{2+} dissociation from NOS-CaM C-terminus		40.0 s^{-1}	[7]
K_D^{NOS1C}	Equilibrium binding of 1st Ca^{2+} to NOS-CaM C-terminus		10.0 μM	[2]
k_{on}^{NOS2C}	2nd Ca^{2+} binding to NOS-CaM C-terminus		10.0 $\mu M^{-1}s^{-1}$	[6]
k_{off}^{NOS2C}	2nd Ca^{2+} dissociation from NOS-CaM C-terminus		1.0 s^{-1}	[25]
K_D^{NOS2C}	Equilibrium binding of 2nd Ca^{2+} to NOS-CaM C-terminus		0.1 μM	[2]

References:

[1] S. Pepke et al., "A Dynamic Model of Interactions of Ca^{2+}, Calmodulin, and Catalytic Subunits of Ca^{2+}/Calmodulin-Dependent Protein Kinase II," *PLoS Comput. Biol.*, vol. 6, no. 2, p. e1000675, 2010.

[2] Calculated from the relationship $K_D = \frac{k_{off}}{k_{on}}$

[3] Assume that Ca^{2+} binding does not affect the rate of protein dissociation from CaM

[4] Calculated from the thermodynamic principle of microscopic reversibility

[5] M. Kim et al., "Colocalization of Protein Kinase A with Adenylyl Cyclase Enhances Protein Kinase A Activity during Long-Lasting Long-Term-Potentiation," *PLoS Comput. Biol.*, vol. 7, no. 6, p. e1002084, 2011.

[6] Assume that protein binding does not affect the rate of Ca^{2+} association to CaM

[7] Assume that protein binding does not affect the fast dissociation rate(s) of Ca^{2+} from CaM

[8] N. Masada et al., "Distinct Mechanisms of Calmodulin Binding and Regulation of Adenylyl Cyclases 1 and 8," *Biochem.*, vol. 51, no. 40, pp. 7917-7929, 2012.

[9] A.R. Quintana et al., "Kinetics of calmodulin binding to calcineurin," *BBRC*, vol. 334, pp. 674-680, 2005.

[10] M.J. Hubbard and C.B. Klee, "Calmodulin Binding by Calcineurin," *J. Biol. Chem.*, vol. 262, no. 31, pp. 15062-15070, 1987.

[11] E. Takano, M. Hatanaka, and M. Maki, "Real-time analysis of the calcium-dependent interaction between calmodulin and a synthetic oligopeptide of calcineurin by a surface plasmon resonance biosensor," *FEBS Letters*, vol. 352, pp. 247-250, 1994.*

[12] P.M. Stemmer and C.B. Klee, "Dual Calcium Ion Regulation of Calcineurin by Calmodulin and Calcineurin B," *Biochem.*, vol. 33, pp. 6859-6866, 1994.

[13] K. Torok and D.R. Trentham, "Mechanism of 2-Chloro-(ε-amino-Lys_{75})-[6-[4-(N,N-diethylamino)phenyl]-1,3,5-triazin-4-yl]calmodulin Interactions with Smooth Muscle Myosin Light Chain Kinase and Derived Peptides," *Biochem.*, vol. 33, pp. 12807-12820, 1994.

[14] R. Kasturi, C. Vasulka, and J.D. Johnson, “Ca^{2+}, Caldesmon, and Myosin Light Chain Kinase Exchange with Calmodulin,” *J. Biol. Chem.*, vol. 268, pp. 7958-7964, 1993.

[15] M.C. Potier et al., “The Human Myosin Light Chain Kinase (MLCK) from Hippocampus: Cloning, Sequencing, Expression, and Localization to 3qcen-q21,” *Genomics*, vol. 29, no. 3, pp. 562-570, 1995.[†]

[17] J.D. Johnson et al., “Effects of Myosin Light Chain Kinase and Peptides on Ca^{2+} Exchange with the N- and C-terminal Ca^{2+} Binding Sites of Calmodulin,” *J. Biol. Chem.*, pp. 761-767, 1996.

[18] O.B. Peersen, T.S. Madsen, and J.J. Falke, “Intermolecular tuning of calmodulin by target peptides and proteins: Differential effects of Ca^{2+} binding and implications for kinase activation,” *Protein Sci.*, vol. 6, pp. 794-807, 1997.*

[19] Y. Kubota, J. Putkey, and M. Waxham, “Neurogranin Controls the Spatiotemporal Pattern of Postsynaptic Ca^{2+}/CaM signaling,” *Biophys. J.*, vol. 93, pp. 3848-3859, 2007.

[20] J.L. McMurry et al., “Rate, Affinity and Calcium Dependence of Nitric Oxide Synthase Isoform Binding to the Primary Physiological Regulator Calmodulin,” *FEBS J.*, vol. 278, pp. 4943-4954, 2011.

[21] G. Wu, V. Berka, and A. Tsai, “Binding Kinetics of Calmodulin with Target Peptides of Three Nitric Oxide Synthase Isozymes,” *J. Inorg. Biochem.*, vol. 105, pp. 1226-1237, 2011.*

[22] M. Zoche et al., “Distinct Molecular Recognition of Calmodulin-Binding Sites in the Neuronal and Macrophage Nitric Oxide Synthases: A Surface Plasmon Resonance Study,” *Biochem.*, vol. 35, pp. 8742-8747, 1996.*

[23] E.A. Sheta, K. McMillan, and B.S. Siler Masters, “Evidence for a Bidomain Structure of Constitutive Cerebellar Nitric Oxide Synthase,” *J. Biol. Chem.*, vol. 269, no. 21, pp. 15147-15153, 1994.

[24] B.A. Weissman et al., “Activation and Inactivation of Neuronal Nitric Oxide Synthase: Characterization of Ca^{2+}-dependent [^{125}I]Calmodulin Binding,” *Eur. J. Pharm.*, vol. 435, pp. 9-18, 2002.

[25] A. Persechini, H.D. White, and K.J. Gansz, “Different Mechanisms for Ca^{2+} Dissociation from Complexes of Calmodulin with Nitric Oxide Synthase or Myosin Light Chain Kinase,” *J. Biol. Chem.*, vol. 271, no. 1, pp. 62-67, 1996.

*Not used in calculating average value because study used oligopeptide instead of protein

[†]Used to establish equivalence of smooth muscle and hippocampal MLCK

[‡]Used to select rates of MLCK association to and dissociation from $Ca^{2+}{}_4$CaM

[§]Used to select rates of Ca^{2+} dissociation from NOS-CaM
