# Supplementary material for: Competitive tuning: Competition's role in setting the frequency-dependence of Ca2+-dependent proteins
Source: PLoS Comput Biol. 2017 Nov 6;13(11):e1005820. doi: 10.1371/journal.pcbi.1005820 (PMC5690689; doi:10.1371/journal.pcbi.1005820)
Supplement: S1 Appendix — Detailed explanation of the differences between these two model types. (PDF) [file pcbi.1005820.s002.pdf]

Contents:

- Comparison of Four-State and Nine-State Models
- Figure S1. Comparison of 4-state and 9-state Models of CaM-protein Binding.
- Figure S2. Average Bound Concentrations as a Function of Ca^{2+} Frequency
- Figure S3. Time-course of CaM binding partners bound to various states of CaM

Comparison of Four-State and Nine-State Models

The high cooperativity of Ca^{2+} binding at each CaM terminus has led to the development of several models of Ca^{2+} binding to CaM (see [39, 50, 53]). That is, the binding of individual Ca^{2+} ions at each CaM terminus can each be treated as distinct events, resulting in a thermodynamically complete model of all nine possible Ca^{2+}/CaM states (henceforth referred to as the nine-state model). Alternatively, the binding of both Ca^{2+} ions at each CaM terminus can be treated as a single event, resulting in an approximated model (henceforth referred to as the four-state model). The former model is more biophysically accurate, but the latter model is less computationally complex. Therefore, it is important to determine if the nine-state model is truly necessary, or if the four-state model is sufficient.

To test this, isolated and competitive models were each developed as both nine- and four-state models, and the average bound concentrations (C_b) predicted by these models were compared by dividing four-state model's predictions by those of the nine-state model. Red lines correspond to comparisons between isolated models, while blue lines correspond to comparisons between competitive models. All models included 100 Ca^{2+} spikes introduced at frequencies ranging from 0.1 Hz to 1 kHz. All parameters of the four-state model were derived from the parameters of the nine-state model using a steady-state approximation (see [39]).

The level of disagreement between the four-state and nine-state models varied across targets, frequencies, and models (i.e., isolated and competitive). Overall, the four-state model's predictions for the average bound concentrations of CaM targets ranged from 65% to 200% of

those for the nine-state model. Compared to the potential cumulative error of experimental values and previously-described model assumptions, this deviation was deemed negligible. Furthermore, if we are to trust that the implications of this study are robust to *in vivo* extrapolation, then these implications should also be robust to such small deviations in model outputs. Therefore, we chose to use the four-state model for all subsequent simulations.

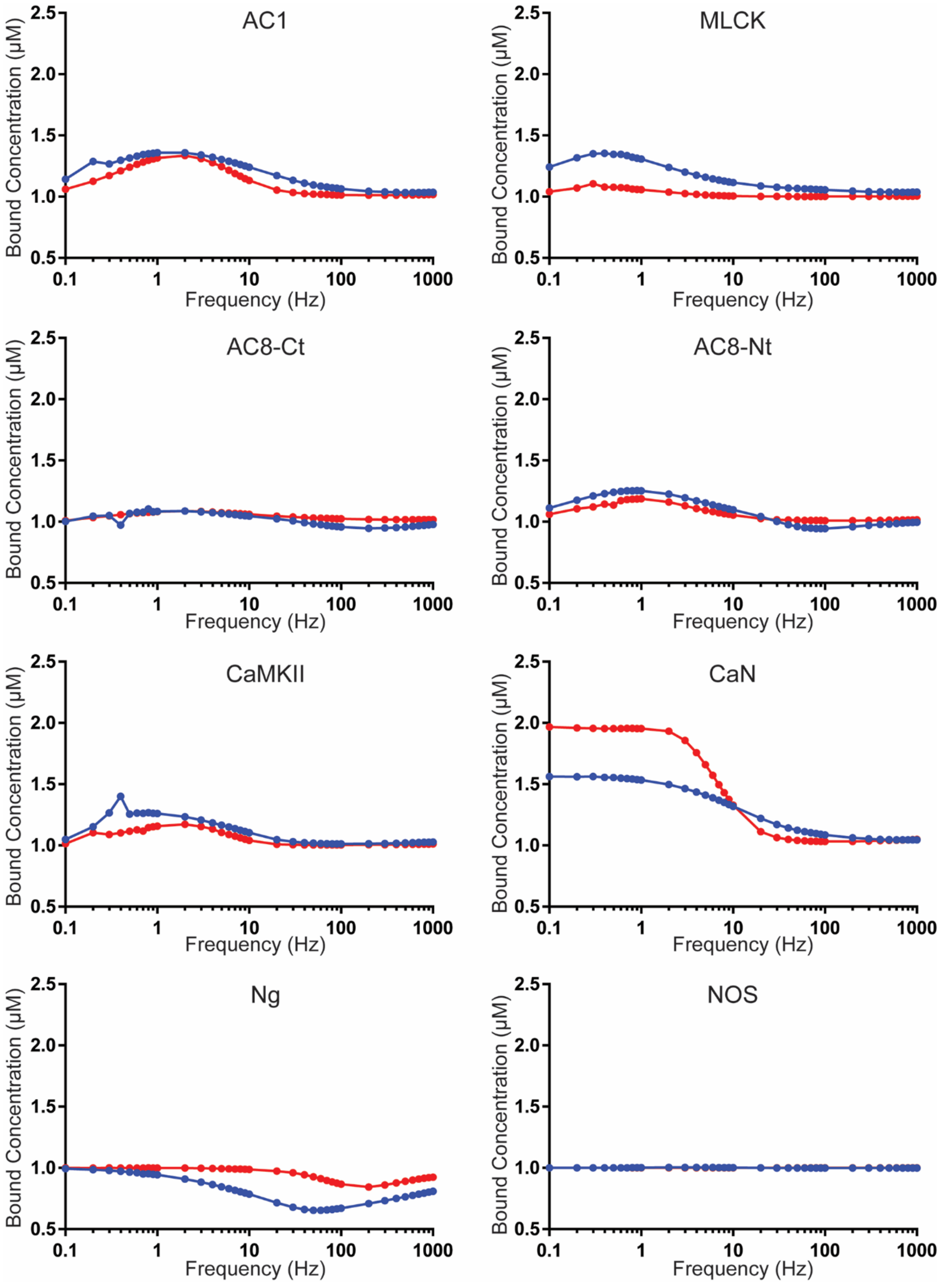

Figure S1. Comparison of 4-state and 9-state models of CaM-protein binding. Each panel corresponds to CaM binding to the titled protein as a function of Ca2+ frequency. Blue traces are the four-state model; red traces are for nine-state descriptions of CaM. For the purposes of this study, the differences between the two model types are negligible and therefore, we use the four-state model of CaM (with the exception of sensitivity analysis).

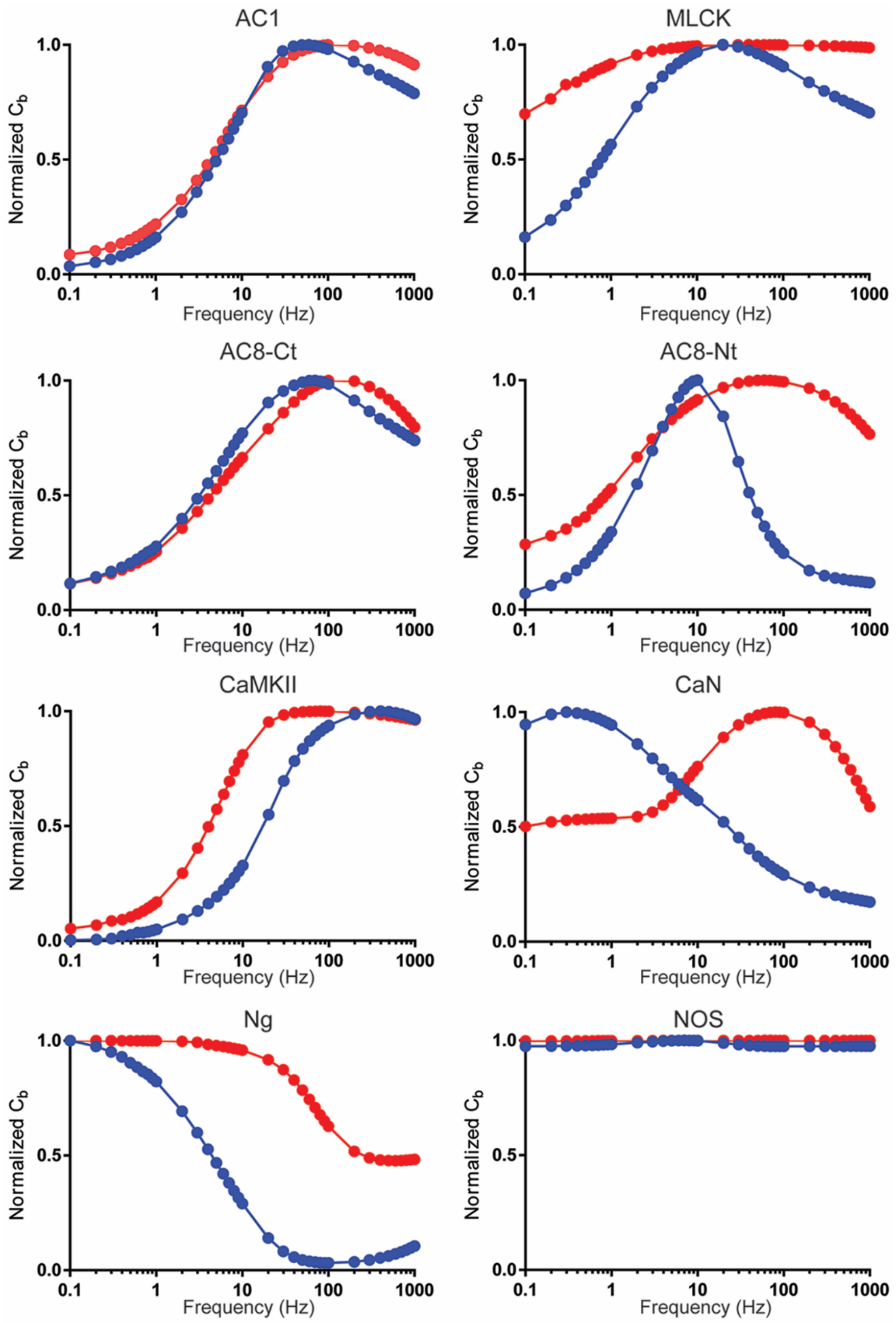

Figure S2. Average Bound Concentrations as a Function of Ca^{2+} Frequency. Data is also shown as heatmaps in Fig 4A and Fig 4B. Here, red traces are the output form the competitive models and blue traces are the output from isolated models.

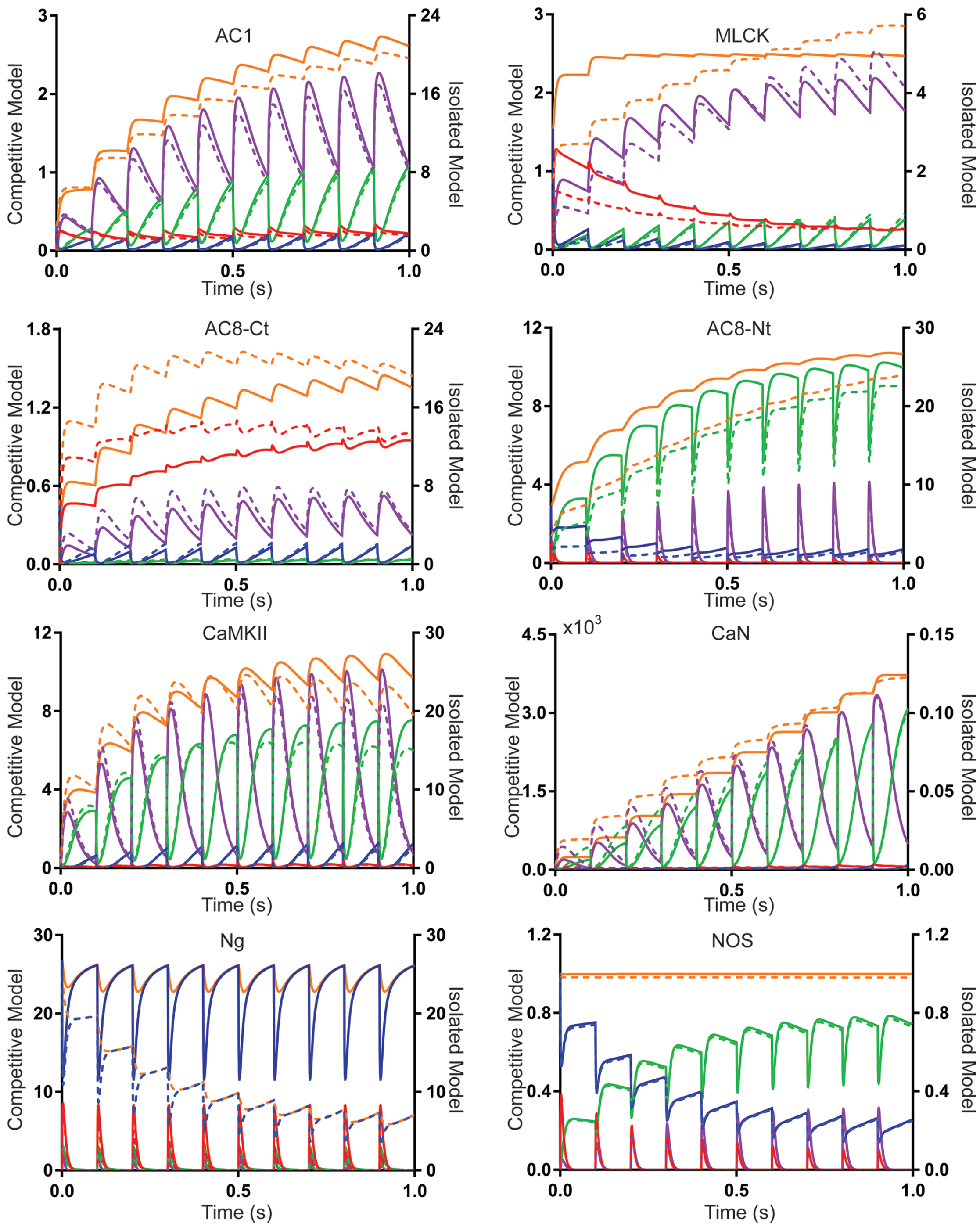

Figure S3. Time-course of CaM binding partners bound to various states of CaM (in micromolar) for 1 second of 10 Hz Ca^{2+} flux. Each plot tracks binding of individual CaM states to the indicated binding partner. **Note differences in scale.** The concentration of indicated binding partner is bound to CaM_0 (blue), CaM_{2N} (red), CaM_{2C} (green), CaM_4 (purple), and CaM_{tot} (orange). Solid lines denote the isolated model plotted against the right axis; dotted lines denote the competitive model plotted against the left axis. The differences between isolated and competitive behavior are more significant for some CaM binding partners than others.
