# Supplementary material for: Competitive tuning: Competition's role in setting the frequency-dependence of Ca2+-dependent proteins
Source: PLoS Comput Biol. 2017 Nov 6;13(11):e1005820. doi: 10.1371/journal.pcbi.1005820 (PMC5690689; doi:10.1371/journal.pcbi.1005820)
Supplement: S2 Appendix — Partial rank correlation coefficients for all initial concentrations and kinetic parameters and their influence on the average bound concentration of CaM to each of its downstream binding partners included in this study. (PDF) [file pcbi.1005820.s003.pdf]

Contents:

- Discussion of Sensitivity Analysis Results
- Table S2. Sensitivity Analysis Results for Varying Initial Concentrations
- Table S3. Sensitivity Analysis Results for Varying Rate Constant Values

Discussion of Sensitivity Analysis Results

Among all initial concentrations, the initial concentration of CaM had the greatest impact on almost all model outputs, regardless of Ca^{2+} frequency. In fact, this held true for all model outputs except the average bound concentration of NOS. This reaffirms one of the observations that motivated this study: that ultimately, the concentration of CaM is the limiting factor of CaM-dependent enzyme activation in dendritic spines. Apart from CaM, Ng, and CaMKII are the only other proteins whose concentration variations significantly impacted (i.e., PRCC greater than 0.5) multiple model outputs. The concentration of Ng had a significant impact on the greatest number of model outputs at 1 Hz and on the least number of model outputs at 100 Hz. Conversely, the concentration of CaMKII had a significant impact on the greatest number of model outputs at 100 Hz and on the least number of model outputs at 1 Hz. This implies that Ng serves as the primary CaM buffer at low Ca^{2+} frequencies, while CaMKII serves as the primary CaM buffer at high Ca^{2+} frequencies. In fact, Ng is such an effective CaM buffer at low frequencies that, at 1 Hz, the average bound concentration of AC8-Ct was actually more affected by variations in the concentration of Ng than by variations in its own concentration.

Among all kinetic rate constants, the association rate of CaMKII to CaM_4 (k_{on}^{KCaM4}) significantly impacted the greatest number of model outputs at all Ca^{2+} frequencies. Its variation had a significant impact on five model outputs at 1 Hz, six at 10 Hz, and seven at 100 Hz. At 1 Hz, k_{off}^{NgCaM0}, k_{on}^{Ng2C}, k_{off}^{Ng2C}, and k_{on}^{2N} also significantly impacted several model outputs (five, four, three, and three model outputs, respectively). And at 10 Hz, k_{on}^{1N} significantly impacted

five model outputs. k_{on}^{KCaM4} was the only parameter that significantly impacted more than two model outputs at 100 Hz. Interestingly, the values of all six of these most impactful parameters were obtained from literature. And in fact, the values of very few of the parameters that significantly impacted model outputs were based on the assumptions of this study. This includes both values that were directly assumed and those that were calculated from directly-assumed values. For example, at 1 Hz, just 12 of the 52 significantly impactful parameters were assumed; at 10 Hz, just 4 of the 37 significantly impactful parameters were assumed; and at 100 Hz, just 3 of the 32 significantly impactful parameters were assumed. The parameters that appear to have the greatest effect on model outputs are also those that have been experimentally characterized.

In general, the full results of this global sensitivity analysis could be used by experimenters to prioritize the measurement of rate constants, as some parameters, despite having either no measured value or a contentious value, may ultimately have little effect on relevant output measures.

Table S2. Sensitivity Analysis Results for Varying Initial Concentrations. PRCC values for varying initial protein concentrations. Columns denote initial concentrations; rows denote CaM-bound output.

Constant Initial Concentrations									
1Hz		C_AC1	C_AC8-Ct	C_AC8-Nt	C_CaN	C_CaMKII	C_MLCK	C_Ng	C_NOS
	concCaM	0.952959	0.954758	0.962876	0.945645	0.976528	0.929931	0.976729	0.794361
	concAC1	0.912338	-0.09408	-0.08001	-0.23616	-0.15396	-0.1548	-0.08498	-0.02735
	concAC8ct	-0.00148	0.897934	-0.01571	-0.05863	-0.0159	-0.07945	-0.06604	-0.06916
	concAC8nt	-0.31393	-0.25592	0.908121	-0.32737	-0.53406	-0.22211	-0.22055	-0.01543
	concCaN	0.036581	0.027835	-0.00437	0.932162	0.018235	0.041947	0.013956	-0.00674
	concCaMKII	-0.73987	-0.30047	-0.28354	-0.87643	0.910497	-0.70863	0.151604	-0.0232
	concMLCK	-0.20331	-0.16462	-0.13536	-0.21737	-0.26232	0.927528	-0.16517	-0.02072
	concNg	-0.72015	-0.91221	-0.88906	-0.18266	-0.84416	-0.66441	0.79466	-0.68886
	concNOS	-0.08369	-0.0758	-0.03364	-0.09518	-0.0954	-0.1172	-0.09874	0.996034
10Hz		C_AC1	C_AC8-Ct	C_AC8-Nt	C_CaN	C_CaMKII	C_MLCK	C_Ng	C_NOS
	concCaM	0.9509	0.949779	0.956668	0.956444	0.97659	0.938134	0.975863	0.807618
	concAC1	0.920364	-0.12375	-0.1412	-0.29288	-0.29127	-0.13659	-0.18439	-0.07299
	concAC8ct	-0.14049	0.902862	-0.04429	-0.10434	-0.11449	-0.05375	-0.16409	-0.04491
	concAC8nt	-0.43869	-0.24723	0.9116	-0.41397	-0.66574	-0.25643	-0.44724	-0.0492
	concCaN	0.01596	0.030503	0.025361	0.944233	0.038689	-0.01906	0.001559	0.048745
	concCaMKII	-0.81769	-0.51861	-0.45092	-0.89995	0.917716	-0.73917	-0.2929	0.003817
	concMLCK	-0.22707	-0.1679	-0.16783	-0.32405	-0.29334	0.934001	-0.17237	-0.03465
	concNg	-0.46226	-0.87519	-0.74411	-0.09377	-0.43526	-0.61667	0.882669	-0.72379
	concNOS	-0.08061	-0.03576	-0.08902	-0.09315	-0.13173	-0.10553	-0.11608	0.99596
100Hz		C_AC1	C_AC8-Ct	C_AC8-Nt	C_CaN	C_CaMKII	C_MLCK	C_Ng	C_NOS
	concCaM	0.962139	0.943993	0.958877	0.93872	0.982692	0.935358	0.974492	0.82285
	concAC1	0.935029	-0.0988	-0.07235	-0.23149	-0.44155	-0.15723	-0.05771	-0.03787
	concAC8ct	-0.11372	0.878528	-0.03797	-0.07548	-0.15584	-0.04341	-0.06905	0.030624
	concAC8nt	-0.2239	-0.14853	0.919727	-0.24417	-0.38543	-0.19203	-0.08357	-0.0152
	concCaN	-0.00346	0.032231	-0.00833	0.921361	0.032549	0.019561	-0.00168	-0.01624
	concCaMKII	-0.9118	-0.64275	-0.63972	-0.89812	0.860292	-0.803	-0.24912	0.024234
	concMLCK	-0.26712	-0.09962	-0.0785	-0.20822	-0.41343	0.935192	-0.09085	-0.01557
	concNg	-0.25592	-0.83771	-0.7505	0.112349	0.288976	-0.49936	0.792419	-0.75266
	concNOS	-0.1405	-0.05433	-0.07082	-0.10349	-0.17101	-0.05583	-0.16126	0.996039

Table S3. Sensitivity Analysis Results for Varying Rate Constant Values. PRCC values for varied rate constant values. Here, rows denote varied input; columns denote the output parameter (as before, concentration of CaM-bound protein).

Constant Kinetic Parameters									
1Hz		C_AC1	C_AC8-Ct	C_AC8-Nt	C_CaN	C_CaMKII	C_MLCK	C_Ng	C_NOS
	kon1N	0.405276	0.539947	-0.37632	0.413439	0.296435	0.554572	-0.24589	0.297035
	koff1N	0.040586	0.030919	0.019915	0.050456	-0.02027	-0.01054	-0.02077	-0.05852
	kon2N	0.466128	-0.31911	0.51252	0.361525	0.521115	0.462081	-0.6016	0.29239
	koff2N	0.028331	0.044832	-0.00901	0.021028	0.003598	0.054893	-0.06758	0.013814
	kon1C	-0.01703	0.021513	-0.00408	0.054363	0.017564	0.015112	-0.052	-0.01609
	koff1C	0.010541	0.038815	-0.00987	0.003785	-0.01435	0.054649	0.01088	-0.01908
	kon2C	-0.02745	0.015128	-0.01053	0.024223	-0.00535	-0.01457	0.004622	-0.05211
	koff2C	-0.0594	0.025935	-0.0563	0.012646	-0.01892	-0.03268	0.037681	0.017721
	konAC11N	0.0107	-0.00831	-0.03057	-0.0214	0.028479	0.001062	-0.01804	-0.01646
	konAC12N	-0.03971	-0.00144	-0.02005	-0.05434	-0.1172	-0.03574	0.067806	-0.0522
	konAC11C	-0.01024	7.68E-05	0.032147	-0.02735	-0.00336	-0.00075	0.015161	0.023515
	konAC12C	-0.01096	-0.01036	0.01905	0.005857	-0.00421	-0.044	0.028471	0.039707
	koffAC11N	0.024214	0.007645	0.001245	0.021947	0.009173	0.017772	0.019073	0.023995
	koffAC12N	-0.05452	-0.01961	-0.06593	-0.06307	-0.00522	-0.01078	0.032592	-0.04653
	koffAC11C	-0.00403	0.005434	-0.08018	-0.02417	-0.03217	0.003171	0.029836	0.046559
	koffAC12C	0.009307	0.023451	-0.06265	-0.04826	-0.04735	-0.00621	0.074663	-0.01382
	konAC1CaM0	0.115696	0.029989	-0.03807	-0.01544	0.000176	0.040415	-0.01707	-0.03825
	konAC1CaM2N	0.157357	0.010749	0.063359	0.077764	0.048142	0.013751	-0.04434	0.084502
	konAC1CaM2C	0.389584	0.044876	-0.06534	-0.03445	-0.01758	-0.00259	-0.03858	-0.07164
	konAC1CaM4	0.872825	-0.12028	-0.13027	-0.16362	-0.19505	-0.24706	-0.1488	-0.10433
	koffAC1CaM0	-0.70848	-0.00092	0.032017	0.065756	0.05172	0.001758	0.173742	0.035986
	koffAC1CaM2N	-0.09447	0.013918	0.041659	-0.00326	-0.00958	-0.06325	-0.00057	-0.01132
	koffAC1CaM2C	-0.66685	0.007967	0.153269	-0.0325	0.031031	-0.01437	0.113529	-0.03586
	koffAC1CaM4	-0.26941	0.046484	-0.08058	0.015604	0.066979	0.033189	0.086984	0.024002
	konAC8ct1N	-0.00507	0.042217	-0.028	0.014798	0.036205	0.032759	0.002565	0.056169
	konAC8ct2N	0.042941	0.003206	0.023011	0.080485	0.025584	0.052485	-0.04838	-0.03339
	konAC8ct1C	0.015675	-0.02135	-0.0346	0.046342	-0.03236	-0.01872	-0.02957	-0.00485
	konAC8ct2C	-0.04712	0.005027	-0.02599	-0.05533	-0.024	-0.06451	0.066572	-0.01199
	koffAC8ct1N	0.014842	-0.04927	0.007033	0.003184	-0.02384	0.037472	-0.03384	0.01869
	koffAC8ct2N	0.036326	-0.03372	0.013843	0.004298	0.03565	0.00954	-0.0065	-0.01084
	koffAC8ct1C	-0.02301	-0.03857	-0.04318	-0.0225	-0.0008	-0.04618	-0.00802	-0.0562
	koffAC8ct2C	-0.01261	0.000312	-0.01975	0.018686	0.019271	-0.0086	-0.01078	-0.02581
	konAC8ctCaM0	-0.02091	0.371302	-0.02101	-0.0097	0.003231	-0.02941	-0.03619	0.031697
	konAC8ctCaM2N	-0.05192	0.555076	-0.03465	-0.03424	0.000934	-0.03097	-0.01199	-0.0235
	konAC8ctCaM2C	-0.00891	0.043146	-0.07508	-0.03975	0.040661	0.002013	0.011036	-0.02842
	konAC8ctCaM4	-0.07809	0.69954	-0.0368	-0.03666	-0.02067	-0.14485	0.000903	-0.02625
	koffAC8ctCaM0	-0.00141	-0.71155	0.024042	0.011463	0.062601	0.041724	0.062892	0.034822
	koffAC8ctCaM2N	-0.03709	-0.63203	0.004501	-0.03595	0.053655	0.015432	0.091839	0.038554
	koffAC8ctCaM2C	0.009589	-0.03528	0.009524	-0.00443	0.028691	-0.03913	0.028208	0.024179
	koffAC8ctCaM4	-0.0063	-0.1227	-0.01673	0.04374	-0.01405	0.019035	0.006563	0.018398
	konAC8nt1N	-0.00409	0.017176	-0.01976	0.029047	0.021581	-0.03127	0.017228	0.027782
	konAC8nt2N	0.041063	-0.01171	-0.00225	0.048509	0.005306	0.041306	-0.02111	0.037377
	konAC8nt1C	0.006292	0.01741	0.011342	0.061221	0.082144	0.002483	-0.03321	-0.01409
	konAC8nt2C	-0.00393	0.003782	0.006484	0.023581	-0.00819	-0.0271	0.009444	-0.04992
	koffAC8nt1N	-0.00555	-0.00438	-0.02063	-0.02273	0.019747	0.035619	0.047292	0.024851
	koffAC8nt2N	0.050141	-0.02255	-0.02728	0.029682	0.019499	0.021141	0.03525	0.018172
	koffAC8nt1C	-0.03553	-0.01207	-0.03752	-0.04445	0.018836	-0.03438	0.0463	-0.04262
	koffAC8nt2C	-0.10326	0.028504	-0.16695	-0.00669	-0.1	-0.10151	0.141484	-0.01486
	konAC8ntCaM0	-0.07684	-0.00571	0.135288	-0.01957	0.004489	-0.05117	-0.0653	0.009904
	konAC8ntCaM2N	0.053912	0.035722	-0.00969	0.020233	0.021266	0.067245	-0.04238	-0.02359
	konAC8ntCaM2C	-0.29944	-0.18164	0.878229	-0.15448	-0.31923	-0.27959	-0.55054	-0.11536
	konAC8ntCaM4	-0.02281	-0.0299	0.156313	0.031169	-0.08117	-0.05479	-0.04275	0.008226
	koffAC8ntCaM0	0.069322	0.133115	-0.56072	0.011924	0.058687	0.06772	0.279336	-0.00812
	koffAC8ntCaM2N	0.012644	0.027236	-0.04254	0.096315	-0.01815	0.060727	0.010667	0.010373
	koffAC8ntCaM2C	0.170355	0.134376	-0.79486	0.091415	0.248765	0.124333	0.474833	0.046358
	koffAC8ntCaM4	0.029609	-0.00028	0.029336	0.000317	-0.00551	-0.0237	0.000346	-0.03191
	konPP1N	0.05735	-0.02485	-0.04297	-0.01631	-0.04325	-0.01255	0.040667	0.03004
	konPP2N	-0.02336	-0.03609	0.022196	-0.01204	0.022636	-0.00153	-0.036	0.010106
	konPP1C	0.005988	-0.00403	0.038532	-0.06299	0.041813	-0.02955	0.000317	-0.02795
	konPP2C	-0.04373	0.034723	-0.02146	0.057705	-0.02335	0.037194	0.008342	-0.0216
	koffPP1N	0.060488	-0.02953	0.013626	0.051018	0.061364	0.009555	-0.03329	0.075053
	koffPP2N	0.027299	0.024592	-0.00654	0.042106	0.010477	0.057322	0.011073	-0.01496
	koffPP1C	-0.00166	-0.05085	-0.00199	0.007314	-0.08536	0.029956	0.04896	-0.01693
	koffPP2C	0.02127	-0.00822	0.043713	0.030536	0.034475	0.047844	-0.02607	-0.03808

	konPPCaM0	-0.02026	-0.05149	0.010605	-0.01107	-0.027	-0.04015	0.047817	-0.06079
	konPPCaM2N	-0.02619	-0.01464	-0.02073	0.034108	-0.06015	0.003974	0.024389	-0.0278
	konPPCaM2C	-0.00346	-0.00158	0.02573	-0.06195	-0.02999	-0.07726	0.034795	-0.01658
	konPPCaM4	-0.01209	0.020493	0.052849	0.951262	0.012881	0.013005	-0.09475	0.015251
	koffPPCaM0	-0.00132	0.031398	0.035456	0.032854	-0.02447	0.013793	-0.0195	0.010806
	koffPPCaM2N	0.062317	-0.01554	-0.06674	0.049456	-0.00656	-0.00388	0.043902	-0.00125
	koffPPCaM2C	-0.03411	0.011482	-0.0092	0.04042	0.013413	0.047731	-0.01895	0.058516
	koffPPCaM4	0.013351	0.024521	-0.03494	0.021947	-0.00313	0.029783	-0.0199	-0.02655
	konK1N	-0.01447	0.026201	0.018013	0.046116	0.062701	-0.0309	0.008122	0.017907
	konK2N	-0.03371	0.012941	-0.04798	-0.05169	0.056718	-0.02476	0.036598	0.020792
	konK1C	-0.02944	0.026621	-0.00191	0.002714	-0.03251	-0.00215	-0.00102	0.007195
	konK2C	0.036284	0.060614	0.101847	0.030145	-0.00492	0.04643	-0.05367	-0.01021
	koffK1N	0.025088	-0.02123	0.044422	0.054603	0.030454	-0.04671	-0.02406	-0.03577
	koffK2N	-0.05277	-0.0868	0.250627	-0.09872	-0.49816	-0.09689	0.138291	-0.00949
	koffK1C	0.051344	0.032221	-0.00318	0.017911	-0.03456	-0.00469	-0.01829	0.037701
	koffK2C	-0.05169	0.125271	-0.58312	0.061845	-0.08436	0.003338	0.416509	-0.01291
	konKCaM0	0.04473	0.013024	0.036108	0.022911	0.06451	0.003252	-0.03518	-0.01896
	konKCaM2N	-0.00786	0.003417	0.023211	-0.00202	0.039346	0.057792	-0.01404	0.018667
	konKCaM2C	-0.15175	-0.09088	-0.21715	-0.09824	0.578853	-0.06919	-0.09271	-0.08067
	konKCaM4	-0.83104	-0.58176	0.13559	-0.766	0.486727	-0.86776	0.434339	-0.61228
	koffKCaM0	-0.00534	0.081704	0.00313	-0.01282	-0.58974	0.029488	0.289683	0.07181
	koffKCaM2N	-0.01583	0.021284	0.032505	-0.07523	0.001694	0.00962	0.003799	-0.04757
	koffKCaM2C	0.169627	0.083277	0.517183	0.03635	-0.81685	0.085726	0.143612	0.100069
	koffKCaM4	0.092507	0.070372	-0.0803	0.103246	-0.03981	0.116734	0.002117	0.068663
	konMK1N	0.03366	0.038007	-0.00283	0.017377	-0.02589	-0.0119	-0.0254	-0.01795
	konMK2N	0.003848	0.035	0.031228	0.01734	-0.00166	0.044187	-0.02482	0.029153
	konMK1C	-0.06378	-0.02253	-0.02247	-0.01448	0.022012	-0.02852	0.015328	-0.03498
	konMK2C	0.001336	-0.00348	-0.00976	-0.01996	0.015582	0.012273	-0.02231	0.031107
	koffMK1N	0.007024	0.039638	0.028122	0.035489	-0.03718	-0.05256	-0.00578	0.030876
	koffMK2N	0.008041	0.015569	-0.02328	-0.04746	-0.04536	0.115628	0.012375	-0.0472
	koffMK1C	0.011475	0.043512	0.093163	0.036296	-0.01149	0.041675	-0.04287	0.014578
	koffMK2C	-0.08457	-0.03164	-0.03736	-0.03309	-0.03032	0.001103	0.023243	-0.02761
	konMKCaM0	-0.00524	-0.00248	-0.04535	-0.01224	-0.089	0.166932	-0.00051	0.005637
	konMKCaM2N	-0.00643	-0.01013	-0.04447	0.024562	-0.04435	0.118247	0.014319	0.011975
	konMKCaM2C	0.012105	0.037322	-0.04026	0.016096	-0.00933	0.151988	-0.03814	-0.00812
	konMKCaM4	-0.09823	-0.12694	-0.07653	-0.09891	-0.11908	0.910886	-0.28787	-0.11377
	koffMKCaM0	0.000679	0.037546	0.031585	-0.00056	0.042445	-0.52616	0.114696	0.03478
	koffMKCaM2N	0.001594	0.016316	-0.07571	0.005197	0.014568	-0.14121	0.052985	-0.03984
	koffMKCaM2C	0.014902	0.022153	-0.00258	-0.07356	0.017261	-0.52512	0.087082	-0.01692
	koffMKCaM4	-0.0042	-0.00647	-0.0851	-0.04181	0.025068	-0.35307	0.06873	0.019973
	konNg1N	0.065792	0.196491	-0.06638	0.07535	0.078478	0.084839	-0.01821	0.061287
	konNg2N	0.038799	0.207903	-0.10885	0.019328	0.035593	0.10844	-0.02279	0.013228
	konNg1C	0.476579	0.007384	0.392687	0.366705	0.519212	0.483909	-0.57689	0.315226
	konNg2C	0.508214	0.019027	0.376711	0.390911	0.56326	0.522818	-0.59988	0.280658
	koffNg1N	-0.09936	-0.15345	-0.00535	-0.1087	-0.07993	-0.16544	0.120333	-0.05493
	koffNg2N	-0.05247	-0.23863	0.081406	-0.09652	-0.07342	-0.11739	0.032777	-0.00332
	koffNg1C	-0.46336	-0.00735	-0.36021	-0.33857	-0.52313	-0.48816	0.585916	-0.29053
	koffNg2C	-0.40944	0.036457	-0.54981	-0.24698	-0.53024	-0.41002	0.639088	-0.21679
	konNgCaM0	-0.3177	-0.46632	-0.41858	-0.14542	-0.32019	-0.39666	0.656767	-0.39956
	konNgCaM2N	-0.22221	-0.49987	-0.10491	-0.12735	-0.14911	-0.28157	0.23596	-0.11954
	konNgCaM2C	-0.17386	0.097385	-0.76658	0.036741	-0.393	-0.0218	0.642005	0.022135
	konNgCaM4	-0.05486	-0.02612	-0.01914	-0.0128	-0.06855	-0.06309	0.078703	-0.08447
	koffNgCaM0	0.472967	0.536511	0.549229	0.290114	0.456339	0.541724	-0.74147	0.500383
	koffNgCaM2N	0.226236	0.319073	0.100447	0.121078	0.205849	0.273781	-0.24638	0.163236
	koffNgCaM2C	0.418633	0.051614	0.499953	0.209323	0.442875	0.346034	-0.5608	0.245559
	koffNgCaM4	0.194322	0.077093	0.089132	0.208566	0.260482	0.204988	-0.23568	0.171614
	konNOS1N	-0.01071	-0.00056	-0.0423	-0.02907	-0.07408	0.037509	0.024456	-0.16646
	konNOS2N	0.024967	-0.0314	0.010344	-0.01773	0.021126	0.040333	-0.03888	-0.17251
	konNOS1C	-0.01494	-0.03632	-0.04812	0.013642	-0.07076	-0.02028	0.030216	-0.1021
	konNOS2C	0.116112	0.085491	-0.01385	0.0768	-0.00869	0.04572	-0.03622	-0.04963
	koffNOS1N	-0.0068	0.048725	0.01009	-0.00718	0.026986	-0.02421	0.015568	0.13155
	koffNOS2N	0.063966	0.001084	0.029116	-0.0159	0.040774	0.025033	-0.02556	0.171331
	koffNOS1C	-0.03992	0.043731	0.006024	0.038188	-0.00906	0.0563	0.04357	0.069074
	koffNOS2C	-0.05225	-0.02962	-0.02556	-0.03894	-0.05575	-0.02588	0.060846	0.107712
	konNOSCaM0	0.053566	-0.03596	0.082284	0.078016	0.068076	0.063963	-0.06328	0.364928
	konNOSCaM2N	-0.01646	-0.00541	-0.00613	-0.00427	0.084132	-0.04129	0.013302	-0.00406
	konNOSCaM2C	-0.0053	-0.02803	0.057218	-0.01924	0.0133	0.023657	-0.03296	0.175473
	konNOSCaM4	-0.04583	-0.02296	-0.00724	-0.01994	0.02472	-0.01294	-0.01286	0.911239
	koffNOSCaM0	0.036577	0.02595	0.016415	0.031623	0.031955	0.038228	-0.00307	-0.72161
	koffNOSCaM2N	-0.01243	0.008408	0.088482	-0.06329	0.006278	-0.00356	-0.01361	0.018908
	koffNOSCaM2C	-0.00462	-0.00155	-0.00883	0.035634	0.024145	0.004375	-0.01084	-0.22521
	koffNOSCaM4	-0.04234	-0.03355	0.013181	-0.00183	-0.00594	-0.00527	0.034039	-0.36007

10Hz		C_AC1	C_AC8-Ct	C_AC8-Nt	C_CaN	C_CaMKII	C_MLCK	C_Ng	C_NOS
	kon1N	0.544922	0.630539	-0.68642	0.443254	0.500212	0.642021	-0.36853	0.194233
	koff1N	0.023581	0.014253	0.010625	0.035087	0.073649	0.047867	-0.06525	-0.05791
	kon2N	0.360651	-0.42599	0.375061	0.283905	0.382987	0.336139	-0.62592	0.110454
	koff2N	0.014798	0.002866	-0.02942	-0.06928	0.010166	-0.04822	-0.01552	-0.03055
	kon1C	-0.00626	-0.00156	-0.02647	-0.0339	-0.01809	-0.02026	0.013498	-0.01588
	koff1C	-0.08124	-0.01442	-0.02725	-0.02963	0.010317	0.003176	-0.01206	-0.07975
	kon2C	0.02826	-0.00116	-0.01619	-0.00981	0.055482	0.025687	-0.02451	0.007156
	koff2C	0.049074	0.058415	-0.01451	0.048158	-0.01449	0.037081	-0.03954	-0.02676
	konAC11N	-0.03488	-0.02182	-0.03818	-0.01518	-0.04503	0.005289	0.024516	0.045895
	konAC12N	0.006104	-0.01789	-0.0175	-0.02564	0.004842	0.001513	-0.00126	-0.07748
	konAC11C	0.031368	0.005655	-0.01309	0.00347	0.020379	0.011703	-0.02247	-0.02804
	konAC12C	0.025268	-0.03349	-0.01986	-0.05041	0.041759	0.028977	-0.08376	0.040549
	koffAC11N	-0.02624	0.014046	0.066387	-0.0253	0.057944	0.025892	0.035093	0.118037
	koffAC12N	-0.04738	-0.01519	0.018884	-0.03297	-0.05757	-0.01725	0.038539	-0.09357
	koffAC11C	-0.06097	0.024797	0.00639	0.021686	0.029268	-0.00723	-0.0085	-0.00132
	koffAC12C	-0.01836	-0.03565	0.000264	0.011547	-0.02085	-0.00999	0.001415	-0.0417
	konAC1CaM0	0.071218	-0.03383	0.001697	-0.00383	0.00874	-0.04525	-0.04022	-0.00357
	konAC1CaM2N	0.011057	0.021899	-0.00741	-0.01899	-0.02845	-0.05447	0.033863	0.011525
	konAC1CaM2C	0.290962	0.005804	-0.00823	0.017191	-0.05767	0.014386	-0.03289	-0.02672
	konAC1CaM4	0.924266	-0.21929	-0.1956	-0.21123	-0.40207	-0.33574	-0.14991	-0.1728
	koffAC1CaM0	-0.11429	-0.02108	0.100199	0.055218	-0.00311	-0.02893	0.039126	-0.01337
	koffAC1CaM2N	-0.11116	-0.00736	-0.0248	-0.01311	0.0093	-0.03714	0.000796	0.026685
	koffAC1CaM2C	-0.25385	0.045785	0.053924	-0.0265	-0.02811	-0.00209	0.096384	-0.02214
	koffAC1CaM4	-0.5271	0.101863	0.014513	0.033191	0.098646	0.028271	0.039198	0.057503
	konAC8ct1N	0.021298	0.023967	-0.00832	0.011683	0.016083	-0.01101	-0.05324	-0.01524
	konAC8ct2N	-0.02396	0.026742	-0.0059	0.031227	-0.02103	-0.04087	0.035595	-0.02408
	konAC8ct1C	-0.02029	0.004186	-0.03766	-0.00307	-0.01189	-0.0029	0.044255	0.018465
	konAC8ct2C	-0.01364	0.043672	-0.01219	-0.00465	0.066492	-0.01476	0.044674	0.01071
	koffAC8ct1N	-0.00499	0.057753	0.014119	-0.01989	-0.03318	0.007997	-0.01303	-0.01714
	koffAC8ct2N	-0.03775	-0.01821	-0.00515	-0.01657	-0.01531	0.026674	-0.01019	-0.06794
	koffAC8ct1C	-0.02943	-0.06596	0.023613	-0.04465	-0.04443	-0.00673	0.041994	0.029993
	koffAC8ct2C	-0.07207	-0.05278	-0.034	-0.05984	-0.01908	-0.08479	0.07256	-0.02288
	konAC8ctCaM0	-0.03473	0.222534	0.017625	0.089971	-0.00415	-0.02558	-0.00993	0.039064
	konAC8ctCaM2N	-0.03513	0.591414	-0.00409	-0.03507	-0.05586	-0.03217	-0.04327	-0.00051
	konAC8ctCaM2C	0.008692	0.047819	0.037737	0.056597	0.011658	-0.00912	0.057884	0.051261
	konAC8ctCaM4	-0.11296	0.829469	-0.04111	-0.01236	-0.09661	-0.09013	0.005755	0.00414
	koffAC8ctCaM0	0.014073	-0.26184	0.017485	0.003996	0.039412	0.002007	0.029522	-0.07204
	koffAC8ctCaM2N	0.003228	-0.49715	0.010912	0.002287	0.005378	0.023235	0.026113	-0.02636
	koffAC8ctCaM2C	0.008008	-0.01832	0.007441	-0.0002	0.018409	-0.03681	0.012517	0.050485
	koffAC8ctCaM4	0.07871	-0.3341	-0.01361	-0.03279	0.04656	0.031036	-0.01471	-0.00259
	konAC8nt1N	-0.04188	-0.02644	-0.02173	0.023614	0.001364	0.003556	0.012382	0.04285
	konAC8nt2N	-0.0148	0.051348	-0.00792	0.047135	0.021876	-0.02569	-0.01173	0.042841
	konAC8nt1C	0.019363	0.01049	0.050917	-0.04656	-0.06031	-0.00551	0.013464	0.013529
	konAC8nt2C	-0.00423	0.0295	0.040705	0.019906	0.010199	-0.00815	-0.02318	0.037485
	koffAC8nt1N	0.004385	0.026383	-0.02451	-0.06567	-0.0459	-0.0509	0.04355	-0.0002
	koffAC8nt2N	-0.00171	0.011886	0.001935	0.019774	-0.02062	-0.01404	0.023831	0.032878
	koffAC8nt1C	0.04515	0.071841	0.039863	0.016977	-0.02577	0.080481	-0.00375	0.004152
	koffAC8nt2C	-0.02287	-0.00549	-0.02646	-0.0093	0.014082	0.031214	0.044537	-0.03758
	konAC8ntCaM0	-0.03145	-0.00807	0.102526	-0.00481	-0.06355	-0.01214	-0.00064	-0.01468
	konAC8ntCaM2N	0.013057	-0.0341	-0.03647	-0.0115	-0.09341	-0.04467	0.06252	-0.00036
	konAC8ntCaM2C	-0.35231	-0.24959	0.872526	-0.1531	-0.53773	-0.30122	-0.2229	-0.09578
	konAC8ntCaM4	-0.02182	-0.00091	0.237509	-0.09037	-0.14961	-0.05213	-0.03043	-0.06698
	koffAC8ntCaM0	0.027426	0.036781	-0.18595	0.058664	0.079192	0.041991	0.02326	-0.0108
	koffAC8ntCaM2N	0.023819	0.064614	-0.03271	-0.01565	0.014663	-0.00323	0.019125	0.012398
	koffAC8ntCaM2C	0.122206	0.131506	-0.56628	0.073909	0.206633	0.061692	0.148871	0.037963
	koffAC8ntCaM4	0.049367	0.045801	0.013165	0.01972	-0.04575	0.01988	0.021255	0.009972
	konPP1N	0.042968	-0.00288	-0.02633	0.049743	0.015333	-0.02534	0.004048	0.007851
	konPP2N	-0.00603	-0.03702	0.057197	-0.06113	-0.01512	0.025811	-0.06564	0.053674
	konPP1C	0.005699	0.063927	-0.05853	0.044525	-0.04311	-0.02964	0.035308	-0.04282
	konPP2C	0.041814	-0.01623	-0.01243	0.029077	0.021309	0.004531	0.006206	0.051478
	koffPP1N	0.032621	-0.0027	-0.02612	-0.00331	-0.01289	-0.01076	0.050574	-0.00854
	koffPP2N	0.004474	0.005292	-0.0105	-0.03426	0.03638	-0.07059	0.016431	-0.04911
	koffPP1C	-0.00335	0.011387	0.011329	-0.01587	0.01551	0.024097	-0.04756	0.084171
	koffPP2C	0.002039	0.02187	-0.03775	-0.04383	0.063906	0.010692	0.012056	-8.6E-05

	[illegible]	[illegible]	[illegible]	[illegible]	[illegible]	[illegible]	[illegible]	[illegible]	[illegible]
	koffPP1N	0.032621	-0.0027	-0.02612	-0.00331	-0.01289	-0.01076	0.050574	-0.00854
	koffPP2N	0.004474	0.005292	-0.0105	-0.03426	0.03638	-0.07059	0.016431	-0.04911
	koffPP1C	-0.00335	0.011387	0.011329	-0.01587	0.01551	0.024097	-0.04756	0.084171
	koffPP2C	0.002039	0.02187	-0.03775	-0.04383	0.063906	0.010692	0.012056	-8.6E-05
	konPPCaM0	-0.07652	-0.02603	0.056024	-0.00952	0.004431	-0.04217	0.001859	-0.03531
	konPPCaM2N	-0.0011	-0.03159	0.021221	-0.07922	-0.02736	-0.04943	0.046939	-0.07864
	konPPCaM2C	0.024603	0.051998	-0.0112	0.001628	-0.07819	-0.05401	0.055979	-0.00023
	konPPCaM4	-0.01666	0.074802	0.04998	0.964909	-0.09008	-0.04366	-0.04532	-0.02219
	koffPPCaM0	0.011787	0.022625	0.01468	0.025101	-0.03326	0.016913	0.041721	0.042518
	koffPPCaM2N	-0.02901	-0.02228	0.014898	-0.0129	-0.04448	-0.01629	-0.02853	-0.02723
	koffPPCaM2C	0.03751	0.052076	-0.00023	0.002939	-0.02108	-0.01268	-0.04125	-0.0179
	koffPPCaM4	0.029548	0.010649	0.01263	0.006861	0.029588	0.033326	-0.01485	0.006562
	konK1N	-0.02493	-0.08429	-0.05854	-0.12038	0.169795	-0.00738	-0.09324	0.051854
	konK2N	-0.01449	-0.03275	-0.16445	0.008262	0.172842	-0.00444	-0.09519	0.038366
	konK1C	-0.01202	-0.02407	0.0077	0.015507	0.055329	0.006938	-0.02982	0.022303
	konK2C	-0.01279	0.006783	-0.01526	-0.01725	0.023068	-0.00168	-0.00644	0.011751
	koffK1N	-7.6E-05	0.081729	0.149567	0.002396	-0.1777	0.075027	0.072135	0.063598
	koffK2N	0.041616	0.076174	0.702958	-0.03742	-0.75812	-0.00257	0.472605	0.008362
	koffK1C	-0.03231	0.071424	-0.06794	0.016722	-0.02006	-0.02873	-0.01416	-0.01026
	koffK2C	-0.03579	0.008244	-0.14512	0.035619	-0.01386	0.005354	0.162134	0.014272
	konKCaM0	0.02097	-0.04129	-0.04333	-0.03792	0.030661	0.023012	-0.0314	0.015951
	konKCaM2N	-0.0124	-0.01282	-0.03736	-0.04328	-0.01754	-0.01861	0.011073	-0.02081
	konKCaM2C	-0.13072	-0.11871	-0.36279	-0.07063	0.492016	-0.10805	-0.16618	-0.03155
	konKCaM4	-0.89641	-0.74734	0.10305	-0.81229	0.733015	-0.8983	0.09903	-0.56815
	koffKCaM0	0.057864	0.04953	0.03121	0.022951	-0.06032	0.053477	0.087532	-0.01798
	koffKCaM2N	-0.0249	0.026606	-0.01556	-0.04722	0.02285	-0.01055	0.033208	0.029366
	koffKCaM2C	0.343385	0.186874	0.729379	0.166328	-0.81872	0.236652	0.413691	0.114319
	koffKCaM4	0.246342	0.133446	0.025964	0.18085	-0.14087	0.19928	0.021306	0.089926
	konMK1N	0.006014	-0.00728	0.016089	-0.0461	-0.00114	-0.01577	0.004227	-0.01471
	konMK2N	0.014599	-0.07361	-0.01732	0.019919	0.014762	-0.01657	-0.02542	0.019787
	konMK1C	-0.00723	-0.00607	-0.004	-0.03934	0.02197	-0.00828	-0.04981	-0.00572
	konMK2C	-0.01968	0.000424	0.026162	0.047859	0.059273	0.028692	-0.01357	0.041372
	koffMK1N	0.040837	0.009976	0.02208	-0.02181	-0.02408	0.015292	0.025373	-0.02776
	koffMK2N	-0.00975	0.025674	0.004867	0.028779	0.054486	0.078207	-0.01355	0.008727
	koffMK1C	0.046586	0.048413	-0.03079	0.023255	-0.05487	-0.00813	0.061144	0.001234
	koffMK2C	-0.0587	-0.08723	0.031107	-0.04075	0.064964	-0.02121	-0.04516	0.038277
	konMKCaM0	0.039173	-0.01089	-0.04849	-0.03706	-0.05459	0.263214	-0.02043	0.021496
	konMKCaM2N	0.000921	-0.02448	-0.01381	0.041332	0.020259	0.0841	-0.05624	0.002111
	konMKCaM2C	0.034599	-0.02598	0.012271	-0.0392	-0.01213	0.10332	0.008962	-0.04406
	konMKCaM4	-0.23283	-0.11453	-0.20061	-0.10247	-0.20244	0.93756	-0.04148	-0.05591
	koffMKCaM0	-0.05342	-0.04261	0.024023	-0.05307	0.022094	-0.29099	0.003676	0.013431
	koffMKCaM2N	0.017292	0.058707	-0.01751	0.053138	0.031668	-0.04069	-0.00855	0.053246
	koffMKCaM2C	-0.03693	-0.00473	-0.00773	0.010559	-0.03986	-0.02485	-0.03362	-0.0216
	koffMKCaM4	0.004792	0.040456	0.057444	0.041047	0.010537	-0.38765	0.028722	0.054498
	konNg1N	0.047649	0.217789	-0.09627	0.081189	0.076052	0.140401	-0.04753	0.032874
	konNg2N	0.07526	0.200898	-0.07809	0.084499	-0.01122	0.112309	-0.03077	-0.01438
	konNg1C	0.345947	-0.1145	0.191296	0.253395	0.460173	0.420186	-0.66411	0.225161
	konNg2C	0.370302	-0.12197	0.29923	0.301359	0.387743	0.402083	-0.66448	0.162255
	koffNg1N	-0.10194	-0.21182	0.171408	-0.06378	-0.0365	-0.09775	0.052489	0.00464
	koffNg2N	-0.11303	-0.24554	0.107896	-0.03803	-0.01243	-0.12557	0.026271	-0.00498
	koffNg1C	-0.37953	0.137229	-0.17109	-0.28126	-0.41229	-0.3965	0.644365	-0.12118
	koffNg2C	-0.30304	0.15822	-0.38206	-0.24785	-0.36344	-0.25699	0.635805	-0.08624
	konNgCaM0	-0.19891	-0.33681	-0.28894	-0.07875	-0.17277	-0.34843	0.50209	-0.32786
	konNgCaM2N	-0.17275	-0.50649	0.034355	-0.12623	-0.13049	-0.20754	0.259829	-0.09958
	konNgCaM2C	-0.01867	0.200128	-0.58155	0.095181	-0.14375	0.060959	0.530153	0.087915
	konNgCaM4	-0.09851	-0.02489	-0.04394	-0.08211	-0.06386	-0.06916	0.070109	-0.07642
	koffNgCaM0	0.295726	0.382399	0.363579	0.134383	0.292795	0.430192	-0.67112	0.36234
	koffNgCaM2N	0.156774	0.361352	-0.08026	0.088706	0.171589	0.148485	-0.23818	-0.00317
	koffNgCaM2C	0.224768	-0.08436	0.377685	0.14005	0.274118	0.242065	-0.60932	0.093399
	koffNgCaM4	0.213178	0.050318	0.036832	0.217835	0.224886	0.249856	-0.32076	0.018888
	konNOS1N	-0.03251	-0.05816	0.010101	0.004925	0.025371	-0.04326	0.061237	-0.35839
	konNOS2N	0.019734	-0.01769	-0.00473	-0.01175	-0.05131	0.016631	-0.00879	-0.36344
	konNOS1C	0.063143	0.037564	-0.00538	0.032673	-0.02798	0.056511	0.017219	-0.14346
	konNOS2C	0.002681	-0.02073	0.016273	0.047621	0.036747	0.011016	0.022743	-0.08479
	koffNOS1N	0.060538	0.008222	0.052152	0.057716	0.006634	0.023059	0.010732	0.317381
	koffNOS2N	-0.06321	0.019518	-0.00973	-0.02379	-0.0246	-0.00248	0.009302	0.390239
	koffNOS1C	0.069779	0.033547	-0.02712	-0.0319	-0.02779	-0.03134	0.044669	0.075015
	koffNOS2C	-0.03818	0.061098	0.007763	0.041416	-0.02238	0.005611	-0.00083	0.053778
	konNOSCaM0	0.010969	0.005491	0.039573	-0.02399	0.034914	0.036248	-0.05416	0.371913
	konNOSCaM2N	-0.03035	-0.04935	-0.02155	-0.00071	-0.04224	0.020875	0.025942	-0.04614
	konNOSCaM2C	0.054362	-0.04467	0.079354	0.007519	-0.05001	0.027847	0.006835	0.090478
	konNOSCaM4	-0.06963	-0.04128	-0.02228	-0.06843	0.014857	-0.05768	0.011025	0.896384
	koffNOSCaM0	-0.01592	0.077323	-0.04647	0.037956	0.046247	0.007421	-0.00684	-0.36231
	koffNOSCaM2N	-0.04791	-0.03998	-0.04996	0.031037	0.035418	-0.06014	0.010255	-0.05545
	koffNOSCaM2C	0.015025	0.035334	0.043407	-0.02206	-0.00756	0.003805	0.027613	-0.0328
	koffNOSCaM4	0.004647	0.024864	-0.00934	0.033797	0.014488	-0.0051	-0.01648	-0.69583

100Hz		C_AC1	C_AC8-Ct	C_AC8-Nt	C_CaN	C_CaMKII	C_MLCK	C_Ng	C_NOS
	kon1N	0.261129	0.329881	-0.73569	0.194185	0.337441	0.336218	-0.12389	0.043178
	koff1N	0.037696	0.033897	0.007272	0.001485	-0.04269	0.006729	-0.00029	-0.02132
	kon2N	0.199058	-0.39511	0.162926	0.134012	0.34778	0.161478	-0.44507	0.036031
	koff2N	-0.05436	0.015333	-0.00856	-0.03145	-0.017	-0.01822	0.045868	-0.03928
	kon1C	0.018934	-0.02771	0.043543	-0.00339	-0.03122	0.015434	0.013112	-0.00162
	koff1C	-0.04234	0.008806	0.01197	0.046789	-0.02578	-0.05924	0.022526	0.03538
	kon2C	-0.03571	0.087779	0.049981	0.038551	-0.00662	-0.0085	0.026505	-0.0155
	koff2C	0.024453	0.045036	-0.03175	-0.01634	-0.00057	-0.03792	-0.0128	0.005828
	konAC11N	-0.04326	0.02663	0.04538	-0.01921	-0.04795	-0.05736	0.030674	-0.02892
	konAC12N	0.003678	0.035379	0.010445	-0.06607	-0.04852	0.011019	0.017074	-0.03331
	konAC11C	0.019889	0.072027	0.051872	0.022039	-0.06469	0.020366	-0.00212	0.043866
	konAC12C	-0.05362	0.016905	-0.02473	-0.00874	0.041506	-0.03285	0.006844	0.021487
	koffAC11N	0.003824	-0.02822	-0.0288	-0.00381	-0.02443	0.005797	-0.00965	0.009467
	koffAC12N	-0.05471	-0.02281	0.004082	-0.02404	0.062303	-0.01832	-0.02995	0.024786
	koffAC11C	-0.00834	0.013513	-0.04485	-0.04716	0.06194	-0.0085	-0.01534	-0.01825
	koffAC12C	-0.0177	0.034846	-0.00125	-0.01186	0.054443	0.007342	-0.01073	-0.00685
	konAC1Ca	0.088305	-0.00145	-0.00616	-0.06035	-0.06354	-0.02205	0.008626	-0.00298
	konAC1Ca	0.070092	0.022375	-0.03675	0.054321	-0.02856	0.060017	0.004285	-0.07507
	konAC1Ca	0.051779	0.034312	-0.04417	0.034491	0.053092	-0.00255	-0.04786	-0.00731
	konAC1Ca	0.960128	-0.15946	-0.1963	-0.18626	-0.62742	-0.32477	-0.06451	-0.10625
	koffAC1Ca	-0.17816	-0.03766	0.033384	-0.02528	-0.01307	0.051165	-0.0006	-0.021
	koffAC1Ca	-0.01183	-0.0063	-0.02162	-0.05951	0.062545	-0.00364	-0.04146	-0.00175
	koffAC1Ca	-0.0276	-0.02694	-0.01636	-0.03538	0.007015	-0.00599	-0.02982	-0.03598
	koffAC1Ca	-0.2078	-0.01625	-0.03084	0.002338	-0.00971	0.040968	0.058562	-0.02979
	konAC8ct1	-0.04583	-0.07681	-0.0177	0.001128	0.020743	-0.04037	-0.01171	0.02489
	konAC8ct2	0.012429	-0.007	-0.04065	0.007967	0.022762	-0.02557	0.026674	-0.04068
	konAC8ct1	0.015237	-0.03735	0.034167	0.046794	0.011984	-0.03804	0.03011	0.063986
	konAC8ct2	0.020854	0.063082	0.02727	0.037471	-0.03293	0.001767	-0.04386	0.031855
	koffAC8ct1	0.033368	0.006765	-0.07446	-0.01486	0.013787	0.030217	-0.04859	-0.11031
	koffAC8ct2	0.029258	0.032811	-0.03615	0.043075	0.001856	-0.03072	-0.02879	-0.02214
	koffAC8ct1	0.080766	-0.02769	-0.00446	0.028288	0.038473	0.008088	-0.07054	0.019512
	koffAC8ct2	0.0294	0.020076	0.003561	0.027168	0.008474	-0.02366	-0.07831	-0.01928
	konAC8ct0	0.015254	0.410203	0.009964	-0.00983	-0.10838	-0.06787	0.008836	-0.04995
	konAC8ct0	-0.03204	0.505821	-0.01556	-0.0302	-0.10135	-0.03522	0.014292	0.002152
	konAC8ct0	0.012458	-0.04245	0.009261	-0.02087	-0.0582	0.020836	0.030179	0.015431
	konAC8ct0	-0.10761	0.864256	-0.03144	-0.14782	-0.16263	-0.11506	-0.04676	-0.03946
	koffAC8ct0	0.013319	-0.47339	0.007467	0.053429	0.060489	0.012608	0.00106	-0.01744
	koffAC8ct0	-0.02501	-0.03577	0.01368	0.067602	-0.02677	0.034706	0.063059	0.079226
	koffAC8ct0	0.033929	0.042527	0.038481	0.020758	0.005203	0.001636	-0.01582	-0.00796
	koffAC8ct0	-0.05711	-0.16715	0.038784	0.028463	0.022348	-0.01147	-0.0106	0.005107
	konAC8nt1	0.021718	-0.06481	0.001843	-0.00013	-0.00473	0.014155	0.001149	-0.02631
	konAC8nt2	0.002344	-0.03728	-0.02699	-0.00386	-0.01721	-0.00324	0.042096	-0.03245
	konAC8nt1	-0.04592	0.003185	-0.01741	-0.0208	-0.01465	0.016615	-0.03207	-0.03043
	konAC8nt2	0.01868	-0.0257	-0.02544	0.0307	0.034386	0.083355	-0.03923	0.046555
	koffAC8nt	0.052377	-0.02653	0.052137	0.015691	-0.022	-0.01854	0.018183	0.026807
	koffAC8nt	0.027234	-0.01451	0.037834	-0.05271	-0.04909	-0.06109	0.047494	0.030455
	koffAC8nt	-0.03261	0.011069	-0.01955	-0.03133	-0.00113	0.008041	0.063614	-0.01758
	koffAC8nt	0.019861	-0.00026	0.051806	0.010539	-0.05673	-0.02509	0.053873	-0.03656
	konAC8nt0	-0.09089	-0.05878	0.48868	-0.07119	-0.12543	-0.04948	-0.03797	-0.02218
	konAC8nt0	0.006053	0.014913	0.029317	-0.01344	-0.02216	-0.03396	-0.01523	0.001417
	konAC8nt0	-0.04817	0.013335	0.617608	-0.03904	-0.19926	-0.03869	0.007059	0.024102
	konAC8nt0	-0.11431	-0.04629	0.655022	-0.04355	-0.19343	-0.10175	0.000488	-0.00538
	koffAC8nt	0.074341	0.036689	-0.55287	0.047351	0.132289	0.079464	0.115113	0.024839
	koffAC8nt	0.046538	-0.00481	-0.00948	-0.01228	-0.02414	0.023648	0.036094	-0.03557
	koffAC8nt	0.066265	-0.02003	-0.0092	0.022777	0.018059	-0.01956	0.019018	-0.00124
	koffAC8nt	0.019955	0.024982	-0.0659	0.040237	0.001864	0.016191	0.00244	0.05495
	konPP1N	0.027163	0.002131	-0.00245	-0.04019	-0.0146	-0.00074	0.008457	-0.06161
	konPP2N	0.029006	0.010867	0.00069	0.008638	-0.0366	0.017198	0.045346	0.001726
	konPP1C	0.018342	-0.03417	0.026203	0.008227	-0.01855	0.018802	0.004207	-0.04144
	konPP2C	0.077098	0.004612	0.030553	0.026994	-0.05716	0.018915	0.021911	0.004124

	konPPCaM	0.047614	-0.01861	0.022578	-0.04967	-0.03168	0.01719	0.015966	0.006274
	konPPCaM	-0.02742	0.011815	-0.01858	-0.03203	-0.02436	-0.04344	0.054373	0.012543
	konPPCaM	0.076613	0.014848	0.021937	0.069158	0.014274	0.048386	-0.02337	-0.07065
	konPPCaM	-0.09682	-0.01541	-0.02021	0.969776	-0.05852	0.004644	-0.02329	0.009385
	koffPPCaM	-0.03558	0.009595	0.055205	0.075392	0.006657	0.023349	0.002355	0.020902
	koffPPCaM	0.009441	-0.05845	0.031725	-0.00686	-0.00114	-0.01479	-0.0025	-0.05892
	koffPPCaM	-0.02614	0.025474	0.044154	-0.05172	0.043212	0.001912	-0.03926	0.014211
	koffPPCaM	0.011795	0.007328	0.024373	-0.05495	-0.03104	-0.01292	-0.04744	-0.01285
	konK1N	0.035987	-0.00078	0.01269	-0.01241	0.056199	-0.01894	-0.04858	0.050182
	konK2N	0.009036	-0.04887	0.001557	0.020624	0.033946	0.016321	0.012091	-0.04856
	konK1C	-0.01933	0.016879	0.009737	0.029462	0.005127	0.014512	-0.0385	0.001543
	konK2C	0.02243	0.066516	-0.00718	0.032339	-0.01466	0.026238	0.027046	-0.03889
	koffK1N	-0.06265	-0.02905	-0.01456	-0.03651	-0.03724	-0.03446	0.047878	0.015007
	koffK2N	-0.06275	0.014661	0.004341	0.044237	-0.02196	-0.01692	0.005433	-0.00026
	koffK1C	-0.0713	-0.06173	-0.05891	-0.02725	0.017157	-0.05918	0.007125	-0.02233
	koffK2C	-0.00022	0.011229	0.054391	0.021771	-0.00943	0.063586	-0.03933	0.002081
	konKCaM0	-0.04985	0.011691	-0.02869	-0.00119	-0.04394	0.00986	0.039219	0.001111
	konKCaM2	0.027619	-0.01486	0.033247	-0.06235	-0.01521	-0.01346	-0.04083	-0.02977
	konKCaM2	-0.08997	-0.07556	-0.03353	0.006048	0.108565	-0.04515	-0.05639	0.008489
	konKCaM4	-0.94408	-0.81633	-0.59752	-0.85183	0.907054	-0.93125	-0.09472	-0.52881
	koffKCaM0	0.030907	0.039309	0.004389	-0.00862	0.015506	0.007912	-0.01125	-0.00523
	koffKCaM2	0.008085	-0.01241	0.02972	0.067403	0.039857	0.025955	-0.02289	0.010572
	koffKCaM2	0.00777	0.006279	-0.02738	0.008176	-0.00779	-0.01989	0.036784	-0.03114
	koffKCaM4	0.168376	0.074558	0.002238	0.051703	-0.09954	0.163349	-0.03154	0.063152
	konMK1N	-0.03396	-0.06458	-0.00261	-0.00111	0.042451	0.002373	-0.01165	0.001183
	konMK2N	-0.05746	0.022364	-0.02164	0.025955	0.02801	-0.00849	-0.04619	-2.4E-05
	konMK1C	-0.06347	0.018633	-0.06828	0.021698	0.05405	-0.01316	-0.01562	-0.02323
	konMK2C	0.015295	-0.03966	0.007397	0.000666	-0.013	-0.00621	-0.02774	-0.03837
	koffMK1N	-0.01616	-0.00994	-0.04087	-0.01181	0.07244	-0.02247	-0.02747	-0.04192
	koffMK2N	0.003931	-0.04132	-0.0699	-0.03274	-0.0144	-0.00313	0.02119	0.005936
	koffMK1C	-0.04739	0.039868	-0.0222	-0.00257	-0.03688	0.044226	-0.02167	0.007793
	koffMK2C	0.000214	-0.0161	-0.00283	-0.00448	0.020151	-0.00733	-0.05307	-0.0413
	konMKCaM	-0.0268	0.042202	0.015893	-0.01361	-0.04655	0.366082	-0.06871	0.010413
	konMKCaM	-0.04957	-0.03066	0.015503	0.020196	0.040914	-0.00167	0.038668	-0.01419
	konMKCaM	0.005853	0.008014	0.01161	0.002373	0.047731	0.040086	-0.02887	0.054742
	konMKCaM	-0.24995	-0.08236	-0.03991	-0.14474	-0.45268	0.949018	-0.00742	-0.00358
	koffMKCaM	-0.01028	0.013293	0.027351	0.005507	0.030017	-0.3762	0.007834	0.010525
	koffMKCaM	0.037609	-0.0571	-0.0312	-0.02906	0.020759	-0.06352	0.016121	0.024198
	koffMKCaM	-0.06959	-0.05478	0.029622	-0.03272	-0.02375	0.01386	0.00285	0.024191
	koffMKCaM	-0.00652	-0.01549	0.000253	-0.0116	0.027204	-0.1093	-0.0391	0.002682
	konNg1N	0.069411	0.201223	-0.27195	0.046531	0.104689	0.056883	-0.06326	-0.00845
	konNg2N	0.135163	0.145656	-0.28471	0.072611	0.048406	0.114158	-0.06029	-0.04315
	konNg1C	0.302242	-0.10153	0.237237	0.210596	0.598975	0.273499	-0.7834	-0.02548
	konNg2C	0.339421	-0.09528	0.260811	0.22476	0.577692	0.306325	-0.76964	-0.04334
	koffNg1N	-0.09886	-0.17578	0.294238	0.000861	-0.05747	-0.08617	0.044603	-0.07065
	koffNg2N	-0.08045	-0.13719	0.300095	-0.07796	-0.03003	-0.10435	0.001028	0.012703
	koffNg1C	-0.32333	0.096612	-0.24567	-0.18474	-0.573	-0.30121	0.781943	-0.02716
	koffNg2C	-0.20265	0.10154	-0.26835	-0.08665	-0.49473	-0.23663	0.695071	-0.0581
	konNgCaM	-0.03374	-0.39186	-0.51989	0.054263	0.105971	-0.31765	0.314532	-0.33289
	konNgCaM	-0.03844	-0.24237	0.103389	-0.01924	-0.19603	-0.08106	0.26915	0.01292
	konNgCaM	-0.13812	-0.01312	-0.07858	-0.06925	-0.00531	-0.00725	0.046109	-0.00121
	konNgCaM	-0.01538	0.019222	0.005606	-0.09114	-0.10779	-0.07841	0.119906	0.034923
	koffNgCaM	0.146065	0.447671	0.546346	0.019125	0.136612	0.355095	-0.55906	0.35737
	koffNgCaM	0.202537	0.385092	-0.02159	0.077525	0.169383	0.167673	-0.36969	0.042999
	koffNgCaM	0.160406	-0.01354	0.378504	0.044072	0.44247	0.148059	-0.68009	0.034361
	koffNgCaM	0.30244	0.056461	-0.08568	0.189932	0.530223	0.283193	-0.69178	-0.02311
	konNOS1N	-0.01714	0.026733	0.02723	0.007005	0.030999	-0.0099	0.029906	-0.26687
	konNOS2N	0.043685	-0.01779	0.013891	-0.03309	0.060798	0.024061	-0.02535	-0.26352
	konNOS1C	0.026746	-0.03523	-0.03095	-0.02152	0.048818	0.038617	-0.05402	-0.22244
	konNOS2C	-0.01124	-0.04291	0.026796	-0.01284	0.020541	-0.03445	-0.00723	-0.0015
	koffNOS1N	-0.06097	-0.04104	-0.03947	-0.0105	0.032619	-0.02444	0.020886	0.264352
	koffNOS2N	0.046475	0.028401	0.040077	0.009111	-0.05617	0.056137	-0.01701	0.284485
	koffNOS1C	-0.02774	-0.03031	0.036608	0.043924	-0.03671	-0.04923	0.03373	-0.00014
	koffNOS2C	0.019461	0.034945	-0.00448	0.022079	-0.03079	-0.02341	0.010883	-0.06633
	konNOSCa	-0.02866	-0.00852	0.001689	0.027285	0.025244	0.019695	-0.01348	0.429411
	konNOSCa	-0.01059	0.014154	0.053346	-0.05239	-0.0192	0.054794	-0.0475	-0.02489
	konNOSCa	-0.02086	-0.02908	-0.01239	-0.01767	0.026674	0.042591	-0.02133	0.00208
	konNOSCa	-0.06977	0.005601	0.011559	-0.02224	-0.02438	-0.00968	0.025749	0.84582
	koffNOSCa	-0.02337	0.038657	0.044854	0.014239	0.062211	-0.00584	-0.0383	-0.37773
	koffNOSCa	-0.01436	-0.01814	0.019705	-0.02438	-0.04742	-0.00406	0.018851	0.022564
	koffNOSCa	-0.02848	-0.04805	-0.0215	-0.03752	0.026887	0.010099	-0.00631	-0.05962
	koffNOSCa	-0.05811	-0.05152	-0.00099	-0.04384	-0.04385	0.019773	0.015241	-0.82549
