# Supplementary material for: Competitive tuning: Competition's role in setting the frequency-dependence of Ca2+-dependent proteins
Source: PLoS Comput Biol. 2017 Nov 6;13(11):e1005820. doi: 10.1371/journal.pcbi.1005820 (PMC5690689; doi:10.1371/journal.pcbi.1005820)
Supplement: S3 Appendix — (PDF) [file pcbi.1005820.s004.pdf]

Equations for four-state model, generated by the Mathematica module XCellerator. See also our original code at the digital repository listed elsewhere in our paper.

[Ca]'[t] == -2 * konAC1C * ((Ca[t]^2)) * AC1CaM[0, 0][t] - 2 * konAC1N * (Ca[t]^2) * AC1CaM[0, 0][t] + 2 koffAC1C AC1CaM[0, 1][t] - 2 * konAC1N * (Ca[t]^2) * AC1CaM[0, 1][t] + 2 koffAC1N AC1CaM[1, 0][t] -2 * konAC1C * (Ca[t]^2) * AC1CaM[1, 0][t] + 2 koffAC1C AC1CaM[1, 1][t] + 2 * koffAC1N * AC1CaM[1, 1][t] - 2 * konAC8ctC * (Ca[t]^2) * AC8ctCaM[0, 0][t] - 2 * konAC8ctN * (Ca[t]^2) * AC8ctCaM[0, 0][t] + 2 * koffAC8ctC * AC8ctCaM[0, 1][t] - 2 * konAC8ctN * (Ca[t]^2) * AC8ctCaM[0, 1][t] + 2 * koffAC8ctN * AC8ctCaM[1, 0][t] - 2 * konAC8ctC * (Ca[t]^2) * AC8ctCaM[1, 0][t] + 2 * koffAC8ctC * AC8ctCaM[1, 1][t] + 2 * koffAC8ctN * AC8ctCaM[1, 1][t] - 2 * konAC8ntC * (Ca[t]^2) * AC8ntCaM[0, 0][t] - 2 * konAC8ntN * (Ca[t]^2) * AC8ntCaM[0, 0][t] + 2 * koffAC8ntC * AC8ntCaM[0, 1][t] - 2 * konAC8ntN * (Ca[t]^2) * AC8ntCaM[0, 1][t] + 2 * koffAC8ntN * AC8ntCaM[1, 0][t] - 2 * konAC8ntC * (Ca[t]^2) * AC8ntCaM[1, 0][t] + 2 * koffAC8ntC * AC8ntCaM[1, 1][t] + 2 * koffAC8ntN * AC8ntCaM[1, 1][t] - 2 * konC * (Ca[t]^2) * CaM[0, 0][t] - 2 * konN * (Ca[t]^2) * CaM[0, 0][t] + 2 * koffC * CaM[0, 1][t] - 2 * konN * (Ca[t]^2) * CaM[0, 1][t] + 2 * koffN * CaM[1, 0][t] - 2 * konC * (Ca[t]^2) * CaM[1, 0][t] + 2 * koffC * CaM[1, 1][t] + 2 * koffN * CaM[1, 1][t] - 2 * konKC * (Ca[t]^2) * CaMKIICaM[0, 0][t] - 2 * konKN * (Ca[t]^2) * CaMKIICaM[0, 0][t] + 2 * koffKC * CaMKIICaM[0, 1][t] - 2 * konKN * (Ca[t]^2) * CaMKIICaM[0, 1][t] + 2 * koffKN * CaMKIICaM[1, 0][t] - 2 * konKC * (Ca[t]^2) * CaMKIICaM[1, 0][t] + 2 * koffKC * CaMKIICaM[1, 1][t] + 2 * koffKN * CaMKIICaM[1, 1][t] - 2 * konPPC * (Ca[t]^2) * CaNCaM[0, 0][t] - 2 * konPPN * (Ca[t]^2) * CaNCaM[0, 0][t] + 2 * koffPPC * CaNCaM[0, 1][t] - 2 * konPPN * (Ca[t]^2) * CaNCaM[0, 1][t] + 2 * koffPPN * CaNCaM[1, 0][t] - 2 * konPPC * (Ca[t]^2) * CaNCaM[1, 0][t] + 2 * koffPPC * CaNCaM[1, 1][t] + 2 * koffPPN * CaNCaM[1, 1][t] - 2 * konMKC * (Ca[t]^2) * MLCKCaM[0, 0][t] - 2 * konMKN * (Ca[t]^2) * MLCKCaM[0, 0][t] + 2 * koffMKC * MLCKCaM[0, 1][t] - 2 * konMKN * (Ca[t]^2) * MLCKCaM[0, 1][t] + 2 * koffMKN * MLCKCaM[1, 0][t] - 2 * konMKC * (Ca[t]^2) * MLCKCaM[1, 0][t] + 2 * koffMKC * MLCKCaM[1, 1][t] + 2 * koffMKN * MLCKCaM[1, 1][t] - 2 * konNgC * (Ca[t]^2) * NgCaM[0, 0][t] - 2 * konNgN * (Ca[t]^2) * NgCaM[0, 0][t] + 2 * koffNgC * NgCaM[0, 1][t] - 2 * konNgN * (Ca[t]^2) * NgCaM[0, 1][t] + 2 * koffNgN * NgCaM[1, 0][t] - 2 * konNgC * (Ca[t]^2) * NgCaM[1, 0][t] + 2 * koffNgC * NgCaM[1, 1][t] + 2 * koffNgN * NgCaM[1, 1][t] - 2 * konNOSC * (Ca[t]^2) * NOSCaM[0, 0][t] - 2 * konNOSN * (Ca[t]^2) * NOSCaM[0, 0][t] + 2 * koffNOSC * NOSCaM[0, 1][t] - 2 * konNOSN * (Ca[t]^2) * NOSCaM[0, 1][t] + 2 * koffNOSN * NOSCaM[1, 0][t] - 2 * konNOSC * (Ca[t]^2) * NOSCaM[1, 0][t] + 2 * koffNOSC * NOSCaM[1, 1][t] + 2 * koffNOSN NOSCaM[1, 1][t]

[CaM[0, 0]]'[t] == koffAC1CaM0 * AC1CaM[0, 0][t] + koffAC8ctCaM0 * AC8ctCaM[0, 0][t] + offAC8ntCaM0 * AC8ntCaM[0, 0][t] - konAC1CaM0 * AC1[t] * CaM[0, 0][t] - konAC8ctCaM0 * AC8ct[t] * CaM[0, 0][t] - konAC8ntCaM0 * AC8nt[t] * CaM[0, 0][t] - konC * (Ca[t]^2) * CaM[0, 0][t] - konN * (Ca[t]^2) * CaM[0, 0][t] - konKCaM0 * CaMKII[t] * CaM[0, 0][t] - konPPCaM0 * CaN[t] * CaM[0, 0][t] - konMKCaM0 * MLCK[t] * CaM[0, 0][t] - konNgCaM0 * Ng[t] * CaM[0, 0][t] - konNOSCaM0 * NOS[t] *CaM[0, 0][t] + koffC * CaM[0, 1][t] + koffN * CaM[1, 0][t] + koffKCaM0 * CaMKIICaM[0, 0][t] + koffPPCaM0 * CaNCaM[0, 0][t] + koffMKCaM0 * MLCKCaM[0, 0][t] + koffNgCaM0 * NgCaM[0, 0][t] + koffNOSCaM0 * NOSCaM[0, 0][t]

[CaM[0, 1]]'[t] == koffAC1CaM2C * AC1CaM[0, 1][t] + koffAC8ctCaM2C * AC8ctCaM[0, 1][t] + koffAC8ntCaM2C * AC8ntCaM[0, 1][t] + konC * (Ca[t]^2) * CaM[0, 0][t] - koffC * CaM[0, 1][t] -

konAC1CaM2C * AC1[t] * CaM[0, 1][t] - konAC8ctCaM2C * AC8ct[t] * CaM[0, 1][t] - konAC8ntCaM2C * AC8nt[t] * CaM[0, 1][t] - konN * (Ca[t]^2) * CaM[0, 1][t] - konKCaM2C * CaMKII[t] * CaM[0, 1][t] - konPPCaM2C * CaN[t] * CaM[0, 1][t] - konMKCaM2C * MLCK[t] * CaM[0, 1][t] - konNgCaM2C * Ng[t] * CaM[0, 1][t] - konNOSCaM2C * NOS[t] * CaM[0, 1][t] + koffN * CaM[1, 1][t] + koffKCaM2C * CaMKIICaM[0, 1][t] + koffPPCaM2C * CaNCaM[0, 1][t] + koffMKCaM2C * MLCKCaM[0, 1][t] + koffNgCaM2C * NgCaM[0, 1][t] + koffNOSCaM2C * NOSCaM[0, 1][t]

[CaM[1, 0]]'[t] == koffAC1CaM2N * AC1CaM[1, 0][t] + koffAC8ctCaM2N * AC8ctCaM[1, 0][t] + koffAC8ntCaM2N * AC8ntCaM[1, 0][t] + konN * (Ca[t]^2) * CaM[0, 0][t] - koffN * CaM[1, 0][t] - konAC1CaM2N * AC1[t] * CaM[1, 0][t] - konAC8ctCaM2N * AC8ct[t] * CaM[1, 0][t] - konAC8ntCaM2N * AC8nt[t] * CaM[1, 0][t] - konC * (Ca[t]^2) * CaM[1, 0][t] - konKCaM2N * CaMKII[t] * CaM[1, 0][t] - konPPCaM2N * CaN[t] * CaM[1, 0][t] - konMKCaM2N * MLCK[t] * CaM[1, 0][t] - konNgCaM2N * Ng[t] * CaM[1, 0][t] - konNOSCaM2N * NOS[t] * CaM[1, 0][t] + koffC * CaM[1, 1][t] + koffKCaM2N * CaMKIICaM[1, 0][t] + koffPPCaM2N * CaNCaM[1, 0][t] + koffMKCaM2N * MLCKCaM[1, 0][t] + koffNgCaM2N * NgCaM[1, 0][t] + koffNOSCaM2N * NOSCaM[1, 0][t]

[CaM[1, 1]]'[t] == koffAC1CaM4 * AC1CaM[1, 1][t] + koffAC8ctCaM4 * AC8ctCaM[1, 1][t] + koffAC8ntCaM4 * AC8ntCaM[1, 1][t] + konN * (Ca[t]^2) * CaM[0, 1][t] + konC * (Ca[t]^2) * CaM[1, 0][t] - koffC * CaM[1, 1][t] - koffN * CaM[1, 1][t] - konAC1CaM4 * AC1[t] * CaM[1, 1][t] - konAC8ctCaM4 * AC8ct[t] * CaM[1, 1][t] - konAC8ntCaM4 * AC8nt[t] * CaM[1, 1][t] - konKCaM4 * CaMKII[t] * CaM[1, 1][t] - konPPCaM4 * CaN[t] * CaM[1, 1][t] - konMKCaM4 * MLCK[t] * CaM[1, 1][t] - konNgCaM4 * Ng[t] * CaM[1, 1][t] - konNOSCaM4 * NOS[t] * CaM[1, 1][t] + koffKCaM4 * CaMKIICaM[1, 1][t] + koffPPCaM4 * CaNCaM[1, 1][t] + koffMKCaM4 * MLCKCaM[1, 1][t] + koffNgCaM4 * NgCaM[1, 1][t] + koffNOSCaM4 * NOSCaM[1, 1][t]

[AC1]'[t] == koffAC1CaM0 * AC1CaM[0, 0][t] + koffAC1CaM2C * AC1CaM[0, 1][t] + koffAC1CaM2N * AC1CaM[1, 0][t] + koffAC1CaM4 * AC1CaM[1, 1][t] - konAC1CaM0 * AC1[t] * CaM[0, 0][t] - konAC1CaM2C * AC1[t] * CaM[0, 1][t] - konAC1CaM2N * AC1[t] * CaM[1, 0][t] - konAC1CaM4 * AC1[t] * CaM[1, 1][t]

[AC8ct]'[t] == koffAC8ctCaM0 * AC8ctCaM[0, 0][t] + koffAC8ctCaM2C * AC8ctCaM[0, 1][t] + koffAC8ctCaM2N * AC8ctCaM[1, 0][t] + koffAC8ctCaM4 * AC8ctCaM[1, 1][t] - konAC8ctCaM0 * AC8ct[t] * CaM[0, 0][t] - konAC8ctCaM2C * AC8ct[t] * CaM[0, 1][t] - konAC8ctCaM2N * AC8ct[t] * CaM[1, 0][t] - konAC8ctCaM4 * AC8ct[t] * CaM[1, 1][t]

[AC8nt]'[t] == koffAC8ntCaM0 * AC8ntCaM[0, 0][t] + koffAC8ntCaM2C * AC8ntCaM[0, 1][t] + koffAC8ntCaM2N * AC8ntCaM[1, 0][t] + koffAC8ntCaM4 * AC8ntCaM[1, 1][t] - konAC8ntCaM0 * AC8nt[t] * CaM[0, 0][t] - konAC8ntCaM2C * AC8nt[t] * CaM[0, 1][t] - konAC8ntCaM2N * AC8nt[t] * CaM[1, 0][t] - konAC8ntCaM4 * AC8nt[t] * CaM[1, 1][t]

[CaMKII]'[t] == -konKCaM0 * CaMKII[t] * CaM[0, 0][t] - konKCaM2C * CaMKII[t] * CaM[0, 1][t] - konKCaM2N * CaMKII[t] * CaM[1, 0][t] - konKCaM4 * CaMKII[t] * CaM[1, 1][t] + koffKCaM0 * CaMKIICaM[0, 0][t] + koffKCaM2C * CaMKIICaM[0, 1][t] + koffKCaM2N * CaMKIICaM[1, 0][t] + koffKCaM4 * CaMKIICaM[1, 1][t]

[CaN]'[t] == -konPPCaM0 * CaN[t] * CaM[0, 0][t] - konPPCaM2C * CaN[t] * CaM[0, 1][t] - konPPCaM2N * CaN[t] * CaM[1, 0][t] - konPPCaM4 * CaN[t] * CaM[1, 1][t] + koffPPCaM0 * CaNCaM[0, 0][t] + koffPPCaM2C * CaNCaM[0, 1][t] + koffPPCaM2N * CaNCaM[1, 0][t] + koffPPCaM4 * CaNCaM[1, 1][t]

[MLCK]'[t] == -konMKCaM0 * MLCK[t] * CaM[0, 0][t] - konMKCaM2C * MLCK[t] * CaM[0, 1][t] - konMKCaM2N * MLCK[t] * CaM[1, 0][t] - konMKCaM4 * MLCK[t] * CaM[1, 1][t] + koffMKCaM0 * MLCKCaM[0, 0][t] + koffMKCaM2C * MLCKCaM[0, 1][t] + koffMKCaM2N * MLCKCaM[1, 0][t] + koffMKCaM4 * MLCKCaM[1, 1][t]

[Ng]'[t] == -konNgCaM0 * Ng[t] * CaM[0, 0][t] - konNgCaM2C * Ng[t] * CaM[0, 1][t] - konNgCaM2N * Ng[t] * CaM[1, 0][t] - konNgCaM4 * Ng[t] * CaM[1, 1][t] + koffNgCaM0 * NgCaM[0, 0][t] + koffNgCaM2C * NgCaM[0, 1][t] + koffNgCaM2N * NgCaM[1, 0][t] + koffNgCaM4 * NgCaM[1, 1][t]

[NOS]'[t] == -konNOSCaM0 * NOS[t] * CaM[0, 0][t] - konNOSCaM2C * NOS[t] * CaM[0, 1][t] - konNOSCaM2N * NOS[t] * CaM[1, 0][t] - konNOSCaM4 * NOS[t] * CaM[1, 1][t] + koffNOSCaM0 * NOSCaM[0, 0][t] + koffNOSCaM2C * NOSCaM[0, 1][t] + koffNOSCaM2N * NOSCaM[1, 0][t] + koffNOSCaM4 * NOSCaM[1, 1][t]

[AC1CaM[0, 0]]'[t] == -koffAC1CaM0 * AC1CaM[0, 0][t] - konAC1C * (Ca[t]^2) * AC1CaM[0, 0][t] - konAC1N * (Ca[t]^2) * AC1CaM[0, 0][t] + koffAC1C * AC1CaM[0, 1][t] + koffAC1N * AC1CaM[1, 0][t] + konAC1CaM0 * AC1[t] * CaM[0, 0][t]

[AC1CaM[0, 1]]'[t] == konAC1C * (Ca[t]^2) * AC1CaM[0, 0][t] - koffAC1C * AC1CaM[0, 1][t] - koffAC1CaM2C * AC1CaM[0, 1][t] - konAC1N * (Ca[t]^2) * AC1CaM[0, 1][t] + koffAC1N * AC1CaM[1, 1][t] + konAC1CaM2C * AC1[t] * CaM[0, 1][t]

[AC1CaM[1, 0]]'[t] == konAC1NkoffMKCaM2C * MLCKCaM[0, 1][t] + (Ca[t]^2) * AC1CaM[0, 0][t] - koffAC1CaM2N * AC1CaM[1, 0][t] - koffAC1N * AC1CaM[1, 0][t] - konAC1C * (Ca[t]^2) * AC1CaM[1, 0][t] + koffAC1C * AC1CaM[1, 1][t] + konAC1CaM2N * AC1[t] * CaM[1, 0][t]

[AC1CaM[1, 1]]'[t] == konAC1N * (Ca[t]^2) * AC1CaM[0, 1][t] + konAC1C * (Ca[t]^2) * AC1CaM[1, 0][t] - koffAC1C * AC1CaM[1, 1][t] - koffAC1CaM4 * AC1CaM[1, 1][t] - koffAC1N * AC1CaM[1, 1][t] + konAC1CaM4 * AC1[t] * CaM[1, 1][t]

[AC8ctCaM[0, 0]]'[t] == -koffAC8ctCaM0 * AC8ctCaM[0, 0][t] - konAC8ctC * (Ca[t]^2) * AC8ctCaM[0, 0][t] - konAC8ctN * (Ca[t]^2) * AC8ctCaM[0, 0][t] + koffAC8ctC * AC8ctCaM[0, 1][t] + koffAC8ctN * AC8ctCaM[1, 0][t] + konAC8ctCaM0 * AC8ct[t] * CaM[0, 0][t]

[AC8ctCaM[0, 1]]'[t] == konAC8ctC * (Ca[t]^2) * AC8ctCaM[0, 0][t] - koffAC8ctC * AC8ctCaM[0, 1][t] - koffAC8ctCaM2C * AC8ctCaM[0, 1][t] - konAC8ctN * (Ca[t]^2) * AC8ctCaM[0, 1][t] + koffAC8ctN * AC8ctCaM[1, 1][t] + konAC8ctCaM2C * AC8ct[t] * CaM[0, 1][t]

[AC8ctCaM[1, 0]]'[t] == konAC8ctN * (Ca[t]^2) * AC8ctCaM[0, 0][t] - koffAC8ctCaM2N * AC8ctCaM[1, 0][t] - koffAC8ctN * AC8ctCaM[1, 0][t] - konAC8ctC * (Ca[t]^2) * AC8ctCaM[1, 0][t] + koffAC8ctC * AC8ctCaM[1, 1][t] + konAC8ctCaM2N * AC8ct[t] * CaM[1, 0][t]

[AC8ctCaM[1, 1]]'[t] == konAC8ctN * (Ca[t]^2) * AC8ctCaM[0, 1][t] + konAC8ctC * (Ca[t]^2) * AC8ctCaM[1, 0][t] - koffAC8ctC * AC8ctCaM[1, 1][t] - koffAC8ctCaM4 * AC8ctCaM[1, 1][t] - koffAC8ctN * AC8ctCaM[1, 1][t] + konAC8ctCaM4 * AC8ct[t] * CaM[1, 1][t]

[AC8ntCaM[0, 0]]'[t] == -koffAC8ntCaM0 * AC8ntCaM[0, 0][t] - konAC8ntC * (Ca[t]^2) * AC8ntCaM[0, 0][t] - konAC8ntN * (Ca[t]^2) * AC8ntCaM[0, 0][t] + koffAC8ntC * AC8ntCaM[0, 1][t] + koffAC8ntN * AC8ntCaM[1, 0][t] + konAC8ntCaM0 * AC8nt[t] * CaM[0, 0][t]

[AC8ntCaM[0, 1]]'[t] == konAC8ntC * (Ca[t]^2) * AC8ntCaM[0, 0][t] - koffAC8ntC * AC8ntCaM[0, 1][t] - koffAC8ntCaM2C * AC8ntCaM[0, 1][t] - konAC8ntN * (Ca[t]^2) * AC8ntCaM[0, 1][t] + koffAC8ntN * AC8ntCaM[1, 1][t] + konAC8ntCaM2C * AC8nt[t] * CaM[0, 1][t]

[AC8ntCaM[1, 0]]'[t] == konAC8ntN * (Ca[t]^2) * AC8ntCaM[0, 0][t] - koffAC8ntCaM2N * AC8ntCaM[1, 0][t] - koffAC8ntN * AC8ntCaM[1, 0][t] - konAC8ntC * (Ca[t]^2) * AC8ntCaM[1, 0][t] + koffAC8ntC * AC8ntCaM[1, 1][t] + konAC8ntCaM2N * AC8nt[t] * CaM[1, 0][t]

[AC8ntCaM[1, 1]]'[t] == konAC8ntN * (Ca[t]^2) * AC8ntCaM[0, 1][t] + konAC8ntC * (Ca[t]^2) * AC8ntCaM[1, 0][t] - koffAC8ntC * AC8ntCaM[1, 1][t] - koffAC8ntCaM4 * AC8ntCaM[1, 1][t] - koffAC8ntN * AC8ntCaM[1, 1][t] + konAC8ntCaM4 * AC8nt[t] * CaM[1, 1][t]

[CaMKIICaM[0, 0]]'[t] == konKCaM0 * CaMKII[t] * CaM[0, 0][t] - koffKCaM0 * CaMKIICaM[0, 0][t] - konKC * (Ca[t]^2) * CaMKIICaM[0, 0][t] - konKN * (Ca[t]^2) * CaMKIICaM[0, 0][t] + koffKC * CaMKIICaM[0, 1][t] + koffKN * CaMKIICaM[1, 0][t]

[CaMKIICaM[0, 1]]'[t] == konKCaM2C * CaMKII[t] * CaM[0, 1][t] + konKC * (Ca[t]^2) * CaMKIICaM[0, 0][t] - koffKC * CaMKIICaM[0, 1][t] - koffKCaM2C * CaMKIICaM[0, 1][t] - konKN (Ca[t]^2) * CaMKIICaM[0, 1][t] + koffKN * CaMKIICaM[1, 1][t]

[CaMKIICaM[1, 0]]'[t] == konKCaM2N * CaMKII[t] * CaM[1, 0][t] + konKN * (Ca[t]^2) * CaMKIICaM[0, 0][t] - koffKCaM2N * CaMKIICaM[1, 0][t] - koffKN * CaMKIICaM[1, 0][t] - konKC * (Ca[t]^2) * CaMKIICaM[1, 0][t] + koffKC * CaMKIICaM[1, 1][t]

[CaMKIICaM[1, 1]]'[t] == konKCaM4 * CaMKII[t] * CaM[1, 1][t] + konKN * (Ca[t]^2) * CaMKIICaM[0, 1][t] + konKC * (Ca[t]^2) * CaMKIICaM[1, 0][t] - koffKC * CaMKIICaM[1, 1][t] - koffKCaM4 * CaMKIICaM[1, 1][t] - koffKN * CaMKIICaM[1, 1][t]

[CaNCaM[0, 0]]'[t] == konPPCaM0 * CaN[t] * CaM[0, 0][t] - koffPPCaM0 * CaNCaM[0, 0][t] - konPPC * (Ca[t]^2) * CaNCaM[0, 0][t] - konPPN * (Ca[t]^2) * CaNCaM[0, 0][t] + koffPPC * CaNCaM[0, 1][t] + koffPPN * CaNCaM[1, 0][t]

[CaNCaM[0, 1]]'[t] == konPPCaM2C * CaN[t] * CaM[0, 1][t] + konPPC * (Ca[t]^2) * CaNCaM[0, 0][t] - koffPPC * CaNCaM[0, 1][t] - koffPPCaM2C * CaNCaM[0, 1][t] - konPPN * (Ca[t]^2) * CaNCaM[0, 1][t] + koffPPN * CaNCaM[1, 1][t]

[CaNCaM[1, 0]]'[t] == konPPCaM2N * CaN[t] * CaM[1, 0][t] + konPPN * (Ca[t]^2) * CaNCaM[0, 0][t] - koffPPCaM2N * CaNCaM[1, 0][t] - koffPPN * CaNCaM[1, 0][t] - konPPC * (Ca[t]^2) * CaNCaM[1, 0][t] + koffPPC * CaNCaM[1, 1][t]

[CaNCaM[1, 1]]'[t] == konPPCaM4 * CaN[t] * CaM[1, 1][t] + konPPN * (Ca[t]^2) * CaNCaM[0, 1][t] + konPPC * (Ca[t]^2) * CaNCaM[1, 0][t] - koffPPC * CaNCaM[1, 1][t] - koffPPCaM4 * CaNCaM[1, 1][t] - koffPPN * CaNCaM[1, 1][t]

[MLCKCaM[0, 0]]'[t] == konMKCaM0 * MLCK[t] * CaM[0, 0][t] - koffMKCaM0 * MLCKCaM[0, 0][t] - konMKC * (Ca[t]^2) * MLCKCaM[0, 0][t] - konMKN * (Ca[t]^2) * MLCKCaM[0, 0][t] + koffMKC * MLCKCaM[0, 1][t] + koffMKN * MLCKCaM[1, 0][t]

[MLCKCaM[0, 1]]'[t] == konMKCaM2C * MLCK[t] * CaM[0, 1][t] + konMKC * (Ca[t]^2) * MLCKCaM[0, 0][t] - koffMKC * MLCKCaM[0, 1][t] - koffMKCaM2C * MLCKCaM[0, 1][t] - konMKN * (Ca[t]^2) * MLCKCaM[0, 1][t] + koffMKN * MLCKCaM[1, 1][t]

[MLCKCaM[1, 0]]'[t] == konMKCaM2N * MLCK[t] * CaM[1, 0][t] + konMKN * (Ca[t]^2) * MLCKCaM[0, 0][t] - koffMKCaM2N * MLCKCaM[1, 0][t] - koffMKN * MLCKCaM[1, 0][t] - konMKC * (Ca[t]^2) * MLCKCaM[1, 0][t] + koffMKC * MLCKCaM[1, 1][t]

[MLCKCaM[1, 1]]'[t] == konMKCaM4 * MLCK[t] * CaM[1, 1][t] + konMKN * (Ca[t]^2) * MLCKCaM[0, 1][t] + konMKC * (Ca[t]^2) * MLCKCaM[1, 0][t] - koffMKC * MLCKCaM[1, 1][t] - koffMKCaM4 * MLCKCaM[1, 1][t] - koffMKN * MLCKCaM[1, 1][t]

[NgCaM[0, 0]]'[t] == konNgCaM0 * Ng[t] * CaM[0, 0][t] - koffNgCaM0 * NgCaM[0, 0][t] - konNgC * (Ca[t]^2) * NgCaM[0, 0][t] - konNgN * (Ca[t]^2) * NgCaM[0, 0][t] + koffNgC * NgCaM[0, 1][t] + koffNgN * NgCaM[1, 0][t]

[NgCaM[0, 1]]'[t] == konNgCaM2C * Ng[t] * CaM[0, 1][t] + konNgC * (Ca[t]^2) * NgCaM[0, 0][t] - koffNgC * NgCaM[0, 1][t] - koffNgCaM2C * NgCaM[0, 1][t] - konNgN * (Ca[t]^2) NgCaM[0, 1][t] + koffNgN * NgCaM[1, 1][t]

[NgCaM[1, 0]]'[t] == konNgCaM2N * Ng[t] * CaM[1, 0][t] + konNgN * (Ca[t]^2) * NgCaM[0, 0][t] - koffNgCaM2N * NgCaM[1, 0][t] - koffNgN * NgCaM[1, 0][t] - konNgC * (Ca[t]^2) * NgCaM[1, 0][t] + koffNgC * NgCaM[1, 1][t]

[NgCaM[1, 1]]'[t] == konNgCaM4 * Ng[t] * CaM[1, 1][t] + konNgN * (Ca[t]^2) * NgCaM[0, 1][t] + konNgC * (Ca[t]^2) * NgCaM[1, 0][t] - koffNgC * NgCaM[1, 1][t] - koffNgCaM4 * NgCaM[1, 1][t] - koffNgN * NgCaM[1, 1][t]

[NOSCaM[0, 0]]'[t] == konNOSCaM0 * NOS[t] * CaM[0, 0][t] - koffNOSCaM0 * NOSCaM[0, 0][t] - konNOSC * (Ca[t]^2) * NOSCaM[0, 0][t] - konNOSN * (Ca[t]^2) * NOSCaM[0, 0][t] + koffNOSC * NOSCaM[0, 1][t] + koffNOSN * NOSCaM[1, 0][t]

[NOSCaM[0, 1]]'[t] == konNOSCaM2C * NOS[t] * CaM[0, 1][t] + konNOSC * (Ca[t]^2) * NOSCaM[0, 0][t] - koffNOSC * NOSCaM[0, 1][t] - koffNOSCaM2C * NOSCaM[0, 1][t] - konNOSN * (Ca[t]^2) * NOSCaM[0, 1][t] + koffNOSN * NOSCaM[1, 1][t]

[NOSCaM[1, 0]]'[t] == konNOSCaM2N * NOS[t] * CaM[1, 0][t] + konNOSN * (Ca[t]^2) * NOSCaM[0, 0][t] - koffNOSCaM2N * NOSCaM[1, 0][t] - koffNOSN * NOSCaM[1, 0][t] - konNOSC * (Ca[t]^2) * NOSCaM[1, 0][t] + koffNOSC * NOSCaM[1, 1][t]

[NOSCaM[1, 1]]'[t] == konNOSCaM4 * NOS[t] * CaM[1, 1][t] + konNOSN * (Ca[t]^2) * NOSCaM[0, 1][t] + konNOSC * (Ca[t]^2) * NOSCaM[1, 0][t] - koffNOSC * NOSCaM[1, 1][t] - koffNOSCaM4 * NOSCaM[1, 1][t] - koffNOSN * NOSCaM[1, 1][t]
